# Investigating the potential of 6-substituted 3-formyl chromone derivatives as anti-diabetic agents using in silico methods

**DOI:** 10.1038/s41598-024-63237-y

**Published:** 2024-06-08

**Authors:** Minhaz Zabin Saif, Nusrat Jahan Ikbal Esha, Syeda Tasnim Quayum, Shofiur Rahman, Mahmoud A. Al-Gawati, Ghadah Alsowygh, Hamad Albrithen, Abdullah N. Alodhayb, Raymond A. Poirier, Kabir M. Uddin

**Affiliations:** 1 Department of Biochemistry and Microbiology, North South University, Bashundhara,, Dhaka, 1217 Bangladesh; 2 Biological and Environmental Sensing Research Unit, King Abdullah Institute for Nanotechnology, King Saud University, 11451 Riyadh, Saudi Arabia; 3 Research Chair for Tribology, Surface, and Interface Sciences, Department of Physics and Astronomy, College of Science, King Saud University, 11451 Riyadh, Saudi Arabia; 4 Department of Chemistry, Memorial University, St. John’s, Newfoundland A1B 3X7 Canada

**Keywords:** Computational biology and bioinformatics, Drug discovery, Chemistry

## Abstract

In exploring nature's potential in addressing diabetes-related conditions, this study investigates the therapeutic capabilities of 3-formyl chromone derivatives. Utilizing in silico methodologies, we focus on 6-substituted 3-formyl chromone derivatives (1–16) to assess their therapeutic potential in treating diabetes. The research examined the formyl group at the chromone’s C-3 position. ADMET, biological activities, were conducted along with B3LYP calculations using 3 different basis sets. The analogues were analyzed based on their parent structure obtained from PubChem. The HOMO–LUMO gap confirmed the bioactive nature of the derivatives, NBO analysis was performed to understand the charge transfer. PASS prediction revealed that 3-formyl chromone derivatives are potent aldehyde oxidase inhibitors, insulin inhibitors, HIF1A expression inhibitors, and histidine kinase. Molecular docking studies indicated that the compounds had a strong binding affinity with proteins, including CAD, BHK, IDE, HIF-α, p53, COX, and Mpro of SARS-CoV2. 6-isopropyl-3-formyl chromone (4) displayed the highest affinity for IDE, with a binding energy of − 8.5 kcal mol^−1^. This result outperformed the affinity of the reference standard dapagliflozin (− 7.9 kcal mol^−1^) as well as two other compounds that target human IDE, namely vitexin (− 8.3 kcal mol^−1^) and myricetin (− 8.4 kcal mol^−1^). MD simulations were revealed RMSD value between 0.2 and 0.5 nm, indicating the strength of the protein–ligand complex at the active site.

## Introduction

Diabetes mellitus (DM) is a chronic metabolic disorder characterized by elevated postprandial and fasting glucose levels resulting from decreased insulin sensitivity or deficiency^1^. It is projected that by 2045, DM will affect approximately 800 million individuals globally; in 2021 alone, this pandemic disease caused 6.7 million deaths^2,3^. Prolonged hyperglycemia can lead to several complications, including nephropathy, retinopathy, neuropathy, and vascular injury^4^. Inflammation, oxidative stress, and obesity are related symptoms that play a crucial role in the pathogenesis of DM^5–7^. Researchers are continually exploring safe and cost-effective medications to manage DM-related issues, especially in reducing associated complications. Chromone and its derivatives are emerging as potential anti-diabetic agents, making them a promising option for safe anti-diabetic drugs^8^.

Flavonoids are phytochemicals that give plants their characteristic colors and occur naturally in plants' seeds, flowers, fruits, leaves, and bark. They represent the most common phenolics in plants and constitute a broad class of compounds divided into various subclasses, such as anthocyanins, flavanones, isoflavones, flavones, flavanols (catechins), chalcones, and flavonols based on variations in their fundamental structure^9–11^. Flavonoids possess biological and oxidative properties that contribute to their anti-allergic, cardioprotective, anti-diabetic, anti-inflammatory, anti-oxidative, and free radical scavenging actions^9^. Many flavonoids have a chromone structure, which is widely distributed in nature, particularly in the plant kingdom, and exhibit pharmacological activity against influenza, hepatitis B, human rhinovirus (HRV), and neurodegenerative diseases^10^. Chromones have a benzene ring's 5- and 6-positions fused with the c-pyrone nucleus. The chromone derivative, 4-oxo-4H-1-benzopyran-3-carboxaldehyde (chromone 3-carboxaldehyde, C_10_H_6_O_3_), is primarily found in plants^11^.

Chromone is a natural substance found in human and animal diets that is not toxic to mammalian cells^12^. Chromone, both natural and synthetic, have demonstrated interesting biological activities^13^, including medicinal properties such as antimicrobial, fungicidal, antimalarial, anticancer, antiviral effects, antioxidant, anti-HIV, anti-inflammatory, psychotropic, antiviral effects and insecticidal activities, making it a desirable material for drug development^14–17^. Chromones are considered versatile in that they can interact with various receptor classes, and their structure can be modified by attaching different substituents to the benzene or pyrone ring^18^. The Vilsmeir-Haack reaction produces 3-formyl chromone, a frequently used precursor for synthesizing chromone derivatives^19^. Research has shown that 3-formyl chromone and its derivatives have tumor cell-specific cytotoxicity and potent cytotoxic actions against various tumor cells^20^. The electron density can be increased or decreased by the presence of an electron-donating group (EDG) or electron-withdrawing group (EWG) on the carbon atom located at the 6-position^21^. Chromones have anti-inflammatory effects on the COX protein and have been studied for their potential as anti-inflammatory agents. The properties of new compounds of 6-substituted-3-formyl chromone have been assessed using various proteins, such as BHK, p53, CAD of H. pylori, IDE, HIF-α, COX, and Main protease of SARS-CoV-2. Recent research has demonstrated that IDE dysregulation contributes to the development of Type 2 Diabetes (T2D) and Alzheimer's disease (AD). IDE is responsible for regulating the amount of circulating insulin in various organs through a degradation-dependent clearance mechanism. IDE is a significant zinc-metalloprotease degrading several extracellular substrates with pathophysiological significance, including insulin and amyloid-beta protein (A). Given its unique allosteric activation and the use of an oligomer structure to mediate it^21,22^, IDE is a promising therapeutic target for T2D.

Commercially available 4-oxo-4H-1-benzopyran-3-carboxaldehyde (3-formyl chromone) can be dissolved in many solvents, including water, dichloromethane, acetonitrile, DMSO, DMF, ethanol, 2-propanol, and chloroform^23^. It is commonly used as a starting material to synthesize several heterocycles. Benzopyrone derivative 3-formyl chromone exhibits chemically strong reactivity, especially in the presence of nitrogen nucleophilic groups. Its structure includes an unsaturated keto function, a conjugated second carbonyl group at C3, and a highly reactive electrophilic center at C2, along with three electron-deficient sites (C-2, C-4, and -CHO), which can be attacked by strong nucleophilic agents, resulting in the production of various compounds^23,24^.

Recent studies have demonstrated the potent activity of 3-formyl chromone derivatives against Ehrlich Ascites carcinoma cells, indicating their potential as topoisomerase inhibitors^25^. Furthermore, these derivatives have shown promise in blocking the main protease enzyme of coronaviruses, suggesting their potential for coronavirus treatments^26^. In a recent Rao et al.^27^ study, a new series of chromone compounds derived from 7-hydroxy-8-formyl chromone exhibited significant α-glucosidase inhibitory activity compared to the reference molecule Acarbose. The substances were characterized using FTIR, ^1^H, and ^13^C NMR, mass, and UV spectral analyses^27^. Alpha-glucosidase inhibitors (AGIs), a class of anti-diabetic medications, are commonly used to treat type 2 diabetes mellitus^28^.

Previous studies have predominantly focused on using in silico analysis to evaluate the potential of chromones as drug candidates^29–31^. Pharmacokinetic evaluations, drug-likeness experiments, quantum chemical computation, and DFT descriptor values have further supported the promising results and significant interactions of selected natural flavonoids within the binding site of the protein, offering new avenues for developing innovative medications^29^. Quantum chemistry is important in drug discovery as it helps in predicting molecular properties and interactions, aiding in the design of effective and safe drugs^30^.

Apart from the compounds (**1**–**16**) under investigation, additional flavones such as vitexin and myricetin, known for their diverse biological properties, including anti-diabetic, anti-inflammatory, and antioxidant effects, were also studied^30,31^. Recent research^30,31^ has highlighted the anti-diabetic activity of vitexin and myricetin. Their inclusion in our study allowed us to explore the biological characteristics of these compounds and compare them with the compounds being tested.

**Figure 1:**
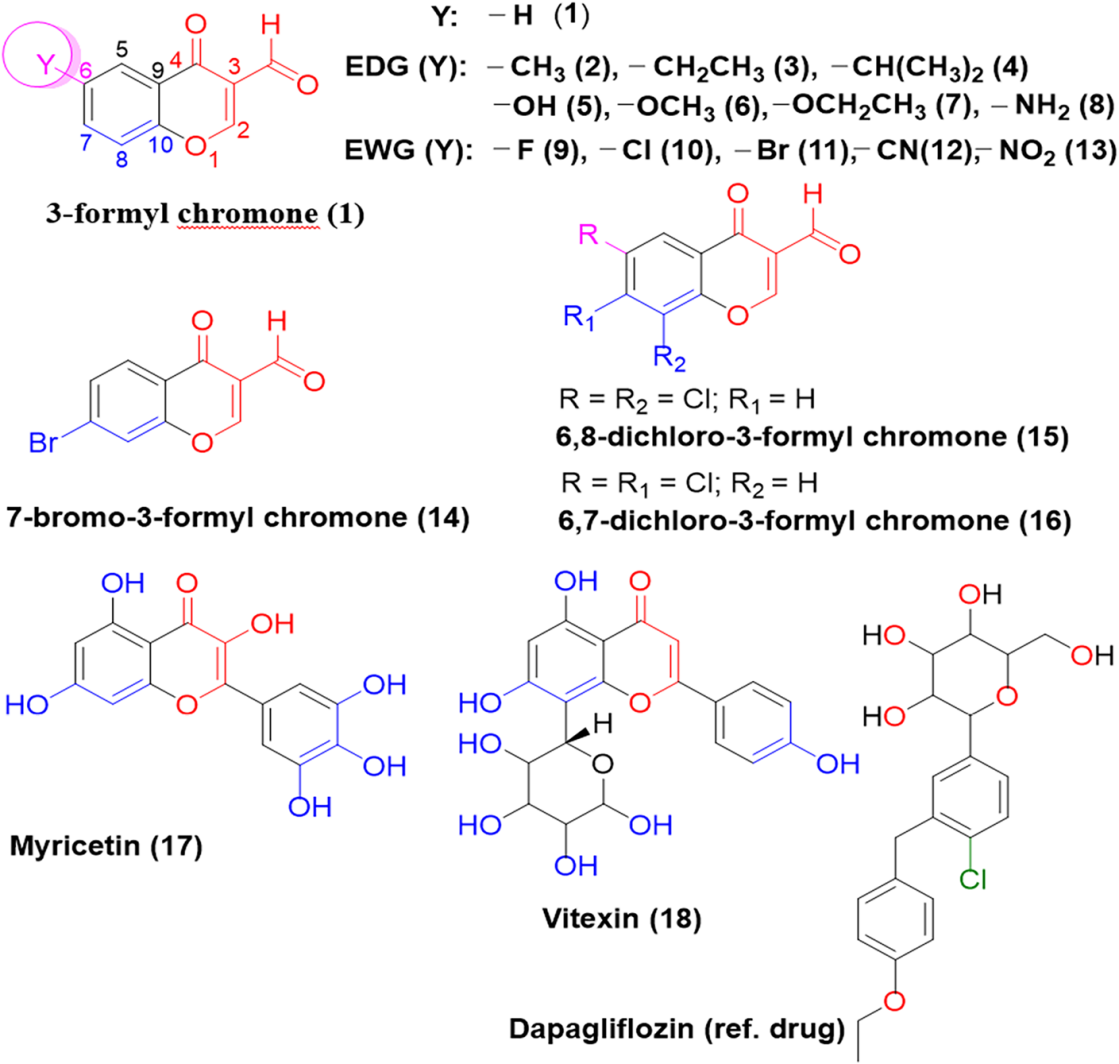
Chemical structures of C_6_ substituted 3-formyl chromone analogues (Y = EDG or EWG) (**1–16**).

Our primary objective was to investigate how EWGs and EDGs affect the biological properties of 6-substituted 3-formyl chromone, as depicted in Fig. 1. Additionally, we examined compounds **14**, **15**, and **16**, which underwent further structural modifications, and compared their activity to compounds **1** to **13**. Several computational methods were employed to gain insights into the compounds' reactivity and stability, including analyzing the HOMO–LUMO gap, chemical hardness (η), chemical potential (μ), and conducting NBO analysis. Furthermore, we conducted an ADMET (Absorption, Distribution, Metabolism, Excretion, and Toxicity) study to evaluate the compounds' toxicity. Molecular docking simulations were performed to assess their potential as anti-diabetic agents. MD simulation analysis was carried out on the protein–ligand interaction site to investigate the stability of the protein–ligand complex. Our study aimed to leverage computational methods to identify novel candidate molecules that could target IDE and potentially offer new avenues for developing anti-diabetic drugs.

The Flow Chart of the study has been shown below.
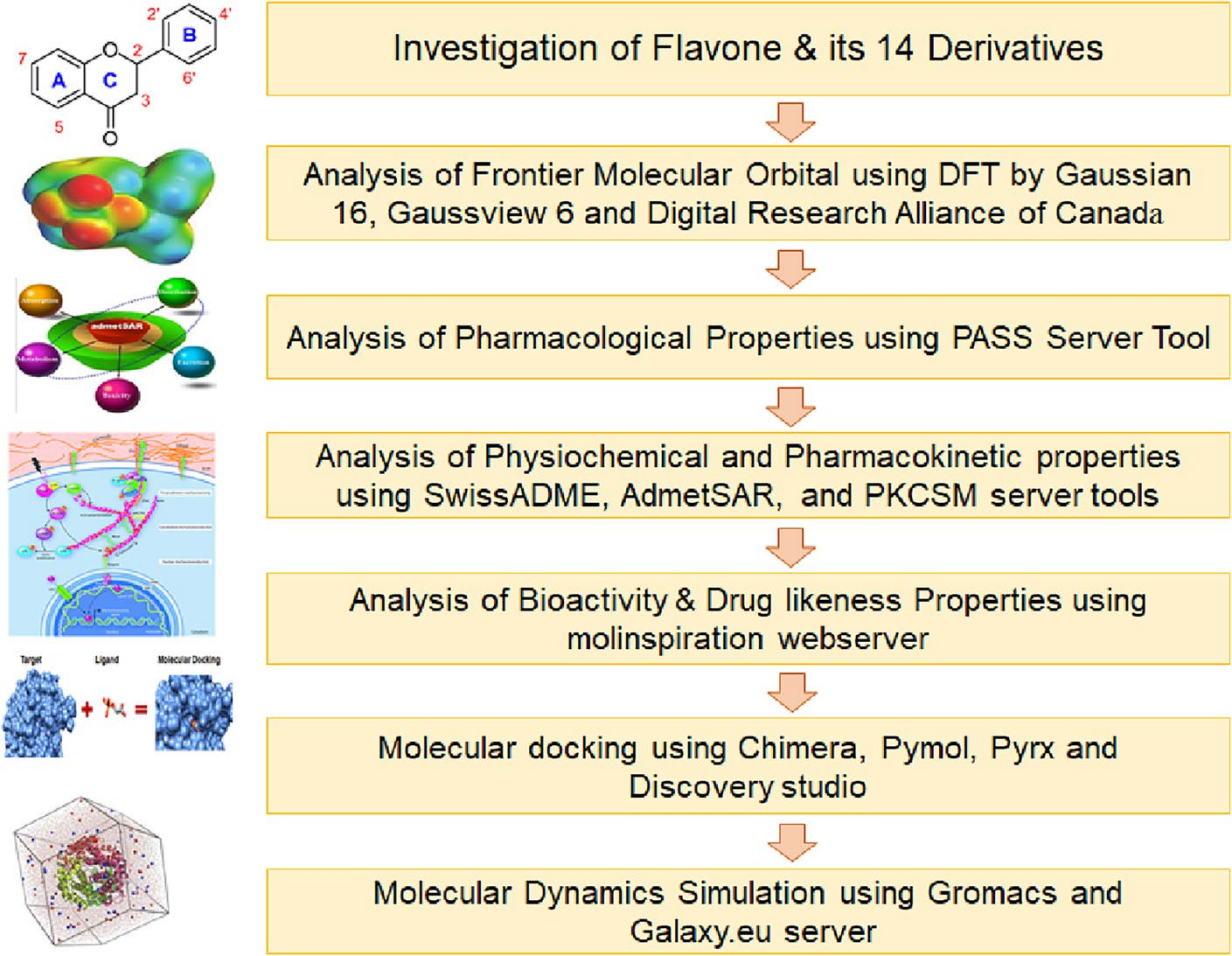


## Methods and computational studies

### Computational analysis

Electronic structure calculations were performed using *Gaussian 16, Revision C.01*^32^. The geometries of 3-formyl chromone derivatives (**1–16**) were optimized using the B3LYP/6-31G(d,p), B3LYP/6-311G(d,p), and B3LYP/6–311 +  + G(d,p) levels of theory. Frequencies were obtained to confirm that the minima had no imaginary frequencies. Experimental data was used to compare the theoretical values and identify the most reliable methods for calculating structural data. Previous research demonstrated^33^ that B3LYP/6-31G(d,p) provided acceptable and computationally efficient results, while G3MP2, G3MP2B3, G4MP2, G3B3, and CBS-QB3 agree within 10 kJ mol^−1^ with each other, and within 18 kJ mol^−1^ with the results of B3LYP/6-31G(d,p). Energy difference from highest occupied molecular orbital (HOMO) to lowest unoccupied molecular orbital (LUMO) and the molecular electrostatic potential (MEP) map were calculated using GaussView 6. The HOMO, LUMO, and MEP maps can be found in Tables S1 to S19 and Figs. S1 to S25 in the supporting information (SI). Unless otherwise specified, all values in the text are for B3LYP/6-31G (d,p). The importance of the Frontier Molecular Orbital (FMO) in maintaining structural stability was demonstrated through the analysis of the HOMO and LUMO^34^. In addition, several chemical descriptors, including Energy Gap (E_Gap_), Ionization Potential (IP), Electron Affinity (EA), Electronegativity (χ), Chemical Potential (μ), Hardness (η), Softness (σ), and Electrophilicity (ω), were calculated for compounds (**1**–**16**) using the equations below^35,36^:

$${\text{E}}_{{{\text{Gap}}}} \;\left( {{\text{eV}}} \right) = \left( {{\text{ELUMO}} - {\text{EHOMO}}} \right);\;{\text{IP}}\;\left( {{\text{eV}}} \right) = - {\text{EHOMO}};\;{\text{EA}}\;\left( {{\text{eV}}} \right) = - {\text{ELUMO}};$$

$$\chi \;\left( {{\text{eV}}} \right) = \left( {{\text{IP}} + {\text{EA}}} \right)/2;\;\mu = - \chi ; \;\eta = \left( {{\text{IP}} - {\text{EA}}} \right)/2;\;\sigma = 1/\eta ;\;\omega = \mu^{2} /2\eta .$$

where μ and η were used to derive the electrophilicity indexes. Mullikan population analysis was employed to calculate the total atomic charges, while NBO analysis was utilized to determine the charges of each individual atom. To obtain atomic charges, single point energy and NBO calculations were conducted^37^.

### Evaluation of physicochemical and pharmacokinetic properties

The physiochemical properties and effectiveness of the 3-formyl chromone derivatives (**1**–**16**) were evaluated using different tools. The swissADME server (www.swissadme.ch) was used to obtain physicochemical properties, medicinal chemistry, and drug-likeness data compiled from the PubChem database (https://pubchem.ncbi.nlm.nih.gov/search/search.cgi)^38^. In contrast, the AdmetSAR tool (http://lmmd.ecust.edu.cn/admetsar2/) and ADMET Predictor software version 9.5 were used to determine and analyze the ADMET properties. Canonical SMILES of each compound were used to obtain several toxicity values, CYP inhibitors, and hERG pIC50 scores. AdmetSAR provided both unregulated and downregulated values for each ADMET property. The Molinspiration online open-access server (https://www.molinspiration.com/cgi-bin/properties)^39^ was used to evaluate the relationship between the compounds' physicochemical properties and molecular activity. The drug-likeness of the compounds was investigated as G-protein coupled receptor (GPCR) ligands, ion channel modulators (ICM), kinase inhibitors (KI), nuclear receptor ligands (NRL), protease inhibitors (PI), and enzyme inhibitors (EI). The structures (**1** − **16**) were drawn in ChemBioDraw Ultra 14.0 to collect MDL Molfile format and then optimized structures converted to the canonical simplified molecular-input system (SMILES) before analysis.

### Pharmacological activities

To determine the potential pharmacological activities of the compounds, the researchers employed the Prediction of Activity Spectra for Substances (PASS) tool, which can provide information on toxicity, mutagenicity, receptor regulation, mechanism, carcinogenicity, teratogenicity, and other pharmacological effects of a compound^40^. Specifically, they used the Pass Online server tool (http://way2drug.com/passonline), which offers access to biochemical information on compounds approved for medicinal use by the USA and the Russian Federation^41^.

### Molecular docking

#### Preparation of ligands

Their SDF files were retrieved from the PubChem database to obtain 3D structures for compounds **1**–**16**, vitexin, myricetin, and the reference drug dapagliflozin. The GaussView 6 was employed to draw all the flavonoid ligands, and their structures were optimized at the B3LYP/6-31G (d,p) level of theory using *Gaussian 16*. Following that, the chemical structures of the selected ligands underwent energy minimization. Lastly, the structures were converted into the PDBQT format using the OpenBabel plugin of PyRx 0.8 software, which can be accessed at https://pyrx.sourceforge.io/^42^.

#### Preparation of target protein

To perform molecular docking, molecular docking software selected appropriate proteins with potential binding sites to the ligands. The crystal structures of the required proteins were retrieved from the RSCB Protein Data Bank tool (https://www.rcsb.org/)^43^, the largest archive of over 505,000,000 atomic coordinate and experimental data files^44^. For this study, a total of seven proteins were selected, including Bacterial Histidine Kinase (PDB ID: 3DGE), Insulin Degrading Enzyme (PDB ID: 6BF8), p53 protein (PDB ID: 7EAX), Alcohol Dehydrogenase of H.pylori (PDB ID: 3TWO), Hypoxia Inducible Factor 1 subunit alpha (PDB ID: 2WA4), Cyclooxygenase (PDB ID: 6Y3C), and SARS-CoV main protease (PDB ID: 6LU7). We were chosen the PDB IDs of our targeted proteins from different literatures. The protein structures were optimized using *Chimera v1.16*^45^, which involved removing water molecules and ligands and energy minimization. The default AMBER ff14SB was used for all the ligands and proteins.

#### Protein–ligand docking

AutoDock Vina^46^ was used to determine the protein–ligand binding regions and identify the amino acid interactions, which served as the target protein for the ligands (**1**–**16**) during molecular docking. A grid box was selected to cover the entire protein, and the docking experiment was conducted with stable proteins and ligands. The protein–ligand binding was validated by re-docking, and the binding modes of the receptor-ligand interactions were visualized using version 1.16 of UCSF Chimera^44^, PyMol version 2.5^47^, and BIOVIA Discovery Studio visualizer^48^. UCSF Chimera identified the amino acids interacting with the ligands^44^, while PyMol generated 3D structures for the molecular docking images. BIOVIA Discovery Studio was used to identify the 2D protein–ligand interactions and determine the hydrogen density around the interacting residues with the protein.

### Molecular dynamics simulations

To investigate the behavior of complex systems and gain more insight than traditional experimental methods, we conducted MD simulations using the *GROMACS version 2021.6*^48^ with the AMBER99SB force field^49^, which provides a detailed description of atom interactions^50^. MD simulations were used to explore the docked complexes of PDB: 6BF8 and 6Y3C with compound **4**. The topology parameters for the proteins were generated using the Galaxy European Server^51^, a popular platform for molecular modeling and simulation. To create a standard salt concentration and neutralize the system, the system was solvated with SPC water molecules in a triclinic box, and Na^+^ ions were added^52^.

The equilibration of the system was achieved through a position-restrained dynamic simulation (NVT) using the leap-frog algorithm at 300 K for 3000ps^53^. This ensured that the pressure and temperature remained constant and that the system reached a stable state. Subsequently, a production run was carried out for another 3000 ps with the same temperature and pressure. Through MD simulations, the current study provided insights into the behavior of the docked protein–ligand complexes. We analyzed MD trajectories using VMD^54^, PyMol^47^, and GROMACS programs. RMSD, Rg, and RMSF were calculated using GROMACS utility gmx rmsd, gmx gyrate, and gmx rmsf for all MD simulations performed at 298 K for 20 ns. Hydrogen bond analysis was performed using the GROMACS utility ‘gmx hbond,’ and temperature and potential energy were also determined.

### PCA analysis

The acquired MD trajectories were subjected to Principal Component Analysis (PCA) using the Bio3D program on the Galaxy European server^55–57^.

## Results and discussion

### Structural parameter variations with method

Single crystals of commercially available compounds, including **2**, **9**, **10**, **11**, **14**, **15**, and **16**, were obtained at room temperature for X-ray diffraction. The crystal data, data collection, and structure refinement details are described in published structure reports^50–54,58–63^. Structural parameters, such as bond lengths and angles, for these compounds, were calculated at the B3LYP level of theory using different basis sets (6-31G(d,p), 6-311G(d,p), and 6–311 +  + G(d,p)). A comparison was made between the computational results and the experimental data to assess the method's precision^54,58–64^. This analysis evaluated any discrepancies or variations between the calculated and experimental data^50–54,58–64^. The structures of compounds **2**, **9**, **10**, **11**, **14**, **15**, and **16**, along with their respective bond lengths and angles obtained from the selected methods, are depicted in Fig. 1 (refer to Figs. S2 to S19 and Tables S1 to S20 in the SI). The summary of the computed and experimental values is presented in Table 1 (See Table S22 in the SI).

**Table 1:** Selected bond distances (Å) and angles (deg) for 3-formyl chromone derivatives.

Bond type	B3LYP/6-31G(d,p)	B3LYP/6-311G(d)	B3LYP/6–311 + + G(d)	Exptl^c^	B3LYP/6-31G(d,p)	B3LYP/6-311G(d,p)	B3LYP/6–311 + + G(d,p)	Exptl^a^
**2**	Bond Distances (Å)				**9** Bond Distance (Å)			
C_6_–CH_3_/F	1.509(0.004)	1.509 (0.004)	1.509 (0.004)	1.505	1.345 (0.016)	1.350 (0.011)	1.347 (0.014)	1.361
C_3_–CHO	1.479(0.004)	1.481(0.006)	1.481(0.006)	1.475	1.481 (0.001)	1.482 (0.002)	1.482 (0.002)	1.480
C_2_–O_1_	1.337 (0.002)	1.335 (0.000)	1.335 (0.000)	1.335	1.338 (0.005)	1.334 (0.009)	1.335 (0.008)	1.343
**2**	Bond Angles (deg)				**9**	Bond Angles (deg)		
C_5_-C_6_-CH_3_/F	121.5(0.3)	121.5(0.3)	121.5(0.3)	111.9	119.5(0.9)	119.3(0.7)	119.3(0.9)	118.6
C_7_-C_6_-CH_3_/F	120.3(0.3)	120.3(0.3)	120.3(0.3)	120.6	118.3(0.3)	118.3(0.1)	118.3(0.3)	118.0
C_4_-C_3_-CHO	124.1(3.7)	124.1(3.8)	124.1(3.8)	120.4	124.0(4.5)	124.5(5.0)	124.0(4.5)	119.5
**10**	Bond Distances (Å)				**11**	Bond Distances (Å)		
C_6_–Cl/Br	1.754(0.016)	1.753(0.015)	1.755(0.017)	1.738	1.908(0.020)	1.913(0.025)	1.914(0.026)	1.888
C_3_–CHO	1.481(0.000)	1.482(0.001)	1.483(0.002)	1.481	1.481(0.003)	1.482(0.004)	1.483(0.005)	1.478
C_2_–O1	1.339(0.002)	1.335(0.006)	1.337(0.004)	1.341	1.339(0.001)	1.336(0.002)	1.337(0.001)	1.338
**10**	Bond Angles (deg)				**11**	Bond Angles (deg)		
C_5_-C_6_-Cl/Br	119.8(0.2)	119.8(0.2)	119.8(0.2)	119.6	119.9(0.1)	119.8(0.0)	119.8(0.0)	119.8
C_7_-C_6_-Cl/Br	118.9(0.0)	118.9(0.0)	118.9(0.0)	118.9	118.9(0.2)	119.0(0.3)	119.0(0.3)	118.7
C_4_-C_3_-CHO	124.0(3.6)	124.5(4.1)	124.0(3.6)	120.4	124.0(3.7)	124.5(4.2)	124.0(3.7)	120.3
MD (Å)	0.677	0.728	0.686		0.812	0.863	0.813	

The values in parenthesis represent the difference between the experimental and calculated values.

MD is the mean deviation for the parenthesis value.

^a^Reference^1–7^.

Upon comparing the optimized and crystal structures of the compounds, we observed that the position of the carbon six (C6) group attached to the chromone ring differed, resulting in calculated bond lengths in the chromone ring that deviated from the crystal data^58–63^. To investigate the impact of EDG and EWG at the C6 position of the chromone ring, we conducted ab initio calculations. Our findings demonstrated that the bond lengths and angles of the EWG series (Y = F, Cl, or Br) at the C6 position increased from fluorine to bromine, with the bromine atom stabilizing the chromone ring due to its high polarizability. In the gas phase, compounds **2** and **10** exhibited mean deviations (MDs) in bond distances ranging from 0.677 Å to 0.728 Å, while compounds** 9** and **11** displayed MDs ranging from 0.812 Å to 0.863 Å. All tested basis sets at the B3LYP level of theory exhibited similar performance in predicting the bond lengths and angles of compounds **2**, **9**, **10**, and **11**. It was observed that the inclusion of diffuse functions in the basis set had minimal impact on the geometries of these compounds at the B3LYP/6–311 +  + G(d,p) level of theory. Specifically, the B3LYP/6-31G(d,p) method provided good agreement with experimental results^64–70^ in estimating the bond lengths and angles of compounds **2**, **9**, **10**, **11**, **14**, **15**, and **16** (Tables 1 and S20 in the SI). Hence, B3LYP/6-31G(d,p) can yield acceptable results while reducing computational time.

### Vibrational spectra

The FTIR spectra of 3-formylchromone (**1**) showed that the characteristic IR bands calculated at B3LYP/6-31G(d,p) and corresponding assignment are presented in Fig. S1 and Table S21 in the SI, which is in good agreement with experimental results^71^. In general, the bands around 1665–1458 cm^−1^ at B3LYP/6-31G(d,p) are closer to experimental values^71^ (around ~ 1652 to ~ 1464 cm^−1^ in the benzene ring are assigned to the skeletal C–C stretching modes. The calculated splitting was observed in the C‒C stretches from C‒CHO for 3-formylchromone has strong and medium bands, which were exhibited as ~ 1380 to ~ 1329 cm^−1^, which is closer to experimental values (~ 1360 to ~ 1340 cm^−1^. In the spectrum of ligand **1**, the strong C=O stretching band in chromone appeared in the range 1690–1630 cm^−1^, and calculated splitting was observed (Fig. S1 and Table S21 in the SI). Similarly, the experimental splitting was observed in the C = O stretches in the same regions. The **1** ligand by both antisymmetric and symmetric vibrations for the C‒H stretching modes was observed at ~ 3005 to ~ 2905 cm^−1^, which is closer to experimental values (~ 2972 to ~ 2900 cm^−1^). There is good agreement between the experimental X-ray data^71^ and theoretical data (Fig. S1 of the SI).

### Analysis of Frontier molecular orbitals

**Figure 2:**
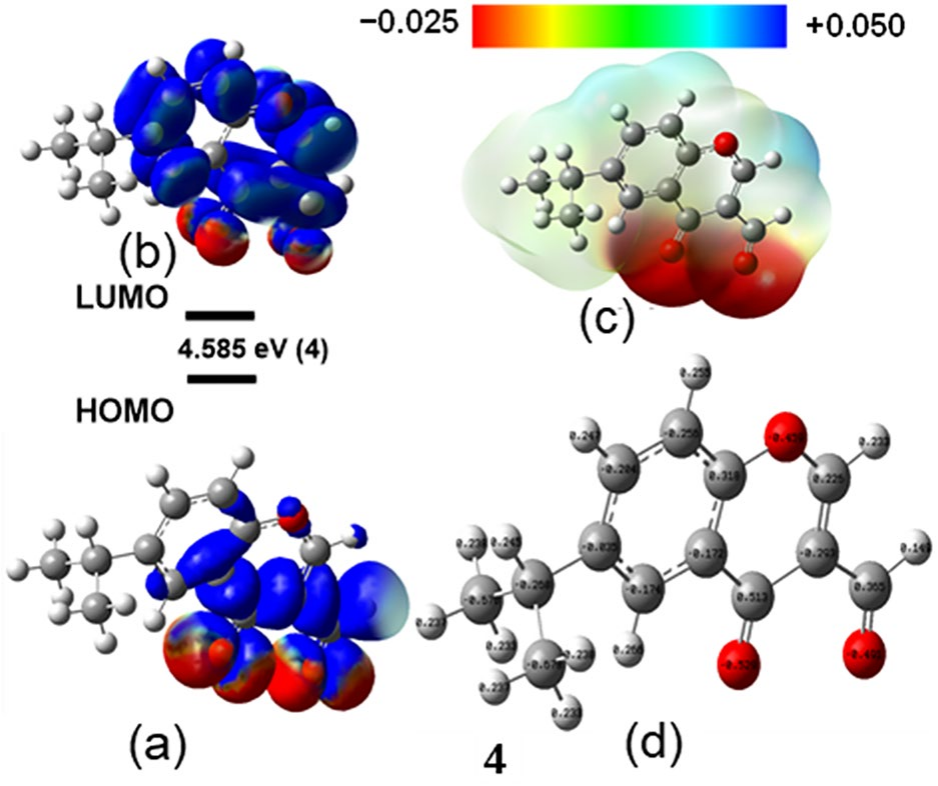
(**a**) HOMO and (**b**) LUMO with Egap; (**c**) Maps of electrostatic potential (0.02 electrons bohr^−3^ surface) (red = electron-rich, blue = electron-deficient) for compound **4**. Regions of respective colours indicating electrophilic (blue) and nucleophilic (red) sites and partial nucleophilic sites (yellow regions); (**d**) NBO charges for compound **4**.

Investigating the FMO in the 3-formyl chromone derivatives (**1**–**16**) offers vital insights into their stability and reactivity^64^. The energy gap (E_gap_) between the HOMO and LUMO known as the HOMO–LUMO energy gap, is a critical factor that determines their chemical reactivity, hardness, softness, chemical potential, and electrophilic index. A narrow E_gap_ indicates softness, signifying high reactivity but low stability, while a wide Egap indicates high stability and low reactivity. The energy levels of the HOMO and LUMO orbitals help in understanding the compounds electron-donating and accepting properties, respectively. Our study presents the findings of FMO analysis, which are summarized in Table 2 and illustrated in Fig. 2 (see Figs. S2 to S19 in the Supplementary Information), providing a comprehensive understanding of the FMOs of the compounds. We have calculated the DFT calculations at 298.15 K. This temperature is close to room temperature and represents conditions relevant to biological systems. Many biological processes, including those targeted by potential drugs, occur at or near body temperature. By performing calculations at 298.15 K, the results become more relevant to understanding how the molecules might behave within a living organism.

**Table 2:** The HOMO and LUMO energies, electronegativity (χ), chemical potential (μ), softness (σ), electrophilicity (ω), and dipole moment (Debye) of all compounds (**1**–**16**) at 298.15 K calculated using B3LYP/6-31G(d,p).

Ligand	E_HOMO_ (eV)	E_LUMO_ (eV)	E_Gap_ (eV)	χ (eV)	μ (eV)	σ (eV)	ω (eV)	Dipole (D)
1	− 5.926	− 2.053	3.874	3.989	− 3.989	0.516	4.108	3.425
2	− 6.424	− 1.822	4.601	4.122	− 4.122	0.434	3.694	6.457
3	− 6.422	− 1.829	4.592	4.126	− 4.126	0.435	3.707	6.401
4	− 6.421	− 1.835	4.585	4.128	− 4.128	0.436	3.716	6.313
5	− 6.430	− 1.856	4.573	4.143	− 4.143	0.437	3.752	7.784
6	− 6.335	− 1.814	4.521	4.075	− 4.075	0.442	3.672	7.763
7	− 6.295	− 1.789	4.505	4.042	− 4.042	0.443	3.626	7.730
8	− 5.925	− 1.692	4.233	3.809	− 3.809	0.472	3.428	6.944
9	− 6.619	− 2.062	4.557	4.340	− 4.340	0.438	4.134	6.410
10	− 6.681	− 2.137	4.543	4.409	− 4.409	0.440	4.279	6.409
11	− 6.672	− 2.129	4.542	4.401	− 4.401	0.440	4.263	6.172
12	− 6.899	− 2.467	4.432	4.683	− 4.683	0.451	4.949	7.531
13	− 6.941	− 2.927	4.014	4.934	− 4.934	0.498	6.067	7.482
14	− 6.655	− 2.117	4.538	4.386	− 4.386	0.441	4.239	4.986
15	− 6.843	− 2.342	4.502	4.592	− 4.592	0.444	4.685	4.822
16	− 6.802	− 2.308	4.493	4.555	− 4.555	0.445	4.618	5.823
Vitexin	− 5.920	− 1.746	4.175	3.833	− 3.833	0.479	3.519	4.274
Myricetin	− 5.679	− 1.722	3.957	3.700	− 3.700	0.505	3.460	6.758
D	− 5.879	− 0.551	5.328	3.215	− 3.215	0.375	1.939	1.999

Calculated using equations in the method section.

D (Reference drug: Dapagliflozin).

The FMO analysis of 3-formyl chromone derivatives (**1**–**16**) reveals essential insights into their stability and reactivity. Among the 6-substituted derivatives (**9**, **10**, and **11**), the HOMO–LUMO gap decreases in the order F > Cl > Br, ranging from 4.557 eV to 4.542 eV (Table 2). Soft ligands (Y = Br) exhibit a smaller HOMO–LUMO gap due to the greater polarizability of their valence electrons compared to fluorine (Table 2). This enhanced polarizability contributes to the electron cloud distortion in C6-halide substituted 3-formyl chromone derivatives. The soft base (Br), characterized by high polarizability and low electronegativity, and further decreases the HOMO–LUMO gap in C6-X substituted chromone rings. Conversely, the hard base (F) with higher electron density exhibits a larger HOMO–LUMO gap. A smaller gap indicates softness, associated with high reactivity and low stability, while a wider gap implies high stability and low reactivity. The energy levels of the HOMO and LUMO orbitals provide insights into the compounds' electron acceptor and donor properties, respectively.

For the 6-hydroxy substituted chromone derivatives, the HOMO–LUMO gap of compound **5** (6-hydroxy) is 4.573 eV, lower than that of compound **4** (6-OCH_2_CH_3_, 4.505 eV) and compound **3** (6-NH_2_, 4.233 eV), due to the electronic effects of the electron-rich chromone ring in 3-formyl chromone derivatives. Additionally, the presence of C6 alkyl substituents (–CH_3_, –CH_2_CH_3_, –CH (CH_3_)_2_) leads to a decreasing trend in the HOMO–LUMO gap, likely due to steric effects (Table 2). Compounds **2**–**16** exhibit higher energy gaps than the parent compound **1**, indicating changes caused by EDG and EWG at the 6-substituted position. Vitexin and myricetin display lower energy gaps than the other compounds (**2**–**16**). The reference drug, dapagliflozin (D), has a higher energy gap than the other compounds. The energy gap values in this series (compounds **1** to **16**) decrease in the following order: **D** (5.328 eV) > **2** (4.601 eV) > **3** (4.592 eV) > **4** (4.585 eV) > **5** (4.573 eV) > **9** (4.557 eV) > **10** (4.543 eV) > **11** (4.542 eV) > **14** (4.538 eV) > **15** (4.502 eV) > **16** (4.493 eV) > **12** (4.432 eV) > **8** (4.233 eV) > vitexin (4.175 eV) > **13** (4.014 eV) > myricetin (3.957 eV) > **1** (3.874 eV).

The results indicate that compounds (**2**–**16**) have higher reactivity than the reference drug D, as evidenced by their higher electronegativity (χ), chemical potential (μ), global hardness (η), and electrophilicity index (ω), as well as their lower softness (σ). The electrophilicity index^65^ is a commonly used parameter for predicting biological activity and identifying reactive sites by measuring the energy lowering resulting from electron transfer between the HOMO and LUMO. Meanwhile, the lower electrophilicity index of compound **8** (3.428 eV) and reference drug **D** (1.939 eV) provides a basis for further analysis of its potential biological activity through molecular docking with a suitable protein. The higher dipole moment of compounds (**2**–**16**) than the reference drug suggested that they may have a better binding affinity. These findings make our compounds promising candidates for further investigation of their biological activity through molecular docking studies.

The DFT results revealed that compound 4 exhibited a strong binding affinity for insulin-degrading enzyme (IDE) with a binding energy of − 8.5 kcal mol^−1^. Moreover, all docking values obtained for the ligands were superior to the reference drug dapagliflozin (IDE: − 7.9 kcal mol^−1^), indicating the potential of the ligands for diabetes treatment. Compound **4** also possesses a smaller HOMO–LUMO gap (4.585 eV) compared to Dapagliflozin (5.328 eV). A smaller HOMO–LUMO gap can be associated with greater ease of electron transfer, potentially contributing to stronger binding interactions with the target protein. For instance, compound **4** demonstrated a Homo–Lumo gap of 4.585, electronegativity (χ) of 4.128, and electrophilicity (ω) of 3.716, which are indicative of its favorable molecular characteristics. These properties align well with its strong binding affinity observed in the DFT calculations. Similarly, while compounds like Vitexin and the parent compound exhibited slightly lower binding affinities compared to compound **4**, their respective molecular properties, such as Homo–Lumo gap and electronegativity, might provide insights into their interaction mechanisms with the receptor.

Furthermore, comparing the molecular properties of compound **4** with other compounds, such as myricetin and dapagliflozin, offers additional insights. Myricetin, with a binding affinity similar to compound 4, demonstrated comparable molecular characteristics, suggesting potential similarities in their interaction mechanisms with the receptor. Conversely, dapagliflozin, despite its lower binding affinity, exhibited distinctive molecular properties that may contribute to its interaction behavior with IDE.

By correlating the DFT results with the docking outcomes, we aim to provide a more holistic understanding of the ligand-receptor interactions and their underlying molecular mechanisms. This integration will enhance the interpretation of our findings and guide future studies aimed at optimizing ligand design for diabetes treatment.

NBO analysis can provide insights into the bonding and stability of molecules by examining the interactions between atoms and the distribution of atomic charges. The stabilization energies of the studied molecules are affected by intramolecular charge transfer, as shown in Figs. S2 to S19 in the SI. MEP maps in Fig. 2 (Figs. S2 to S19 in the SI) visually represent the electrostatic potential of the molecules and may be used to define areas of electrophilicity and nucleophilicity. The colors used in the MEP maps (blue for electrophilic regions and red for nucleophilic regions) indicate the strength of the electrical potential.

Electron-donating groups (EDGs) and electron-withdrawing groups (EWGs) can significantly influence the biological properties of a compound, particularly for small molecules that interact with biological targets like enzymes or receptors. EDGs tend to increase the electron density around a specific atom, making it more willing to accept protons (H +) and enhance the basicity of the compound, potentially affecting its interaction with acidic residues in biological targets. Depending on the functional group, EDGs can improve the water solubility of a compound by creating hydrophilic interactions with water molecules. This can be crucial for drug delivery and absorption in the body. In some cases, EDGs can hinder the metabolism of a compound by enzymes, potentially extending its duration of action in the body. EWGs pull electron density away from a specific atom, making it more acidic (prone to releasing protons). So that EWGs can influence the interaction with basic residues in biological targets. Depending on the functional group, EWGs can sometimes enhance the ability of a compound to pass through cell membranes, which is important for drug delivery^57,72–74^.

The compound **4** has isopropyl group can slightly increase the electron density around the chromone core, potentially influencing its interaction with biological targets. In some antibiotics, the presence of an EDG like an amine group can enhance its hydrogen bonding with bacterial targets, leading to stronger binding and antibacterial activity^75,76^. The position of the EDG or EWG relative to the functional group involved in biological activity can significantly impact the effect. The overall balance of EDGs and EWGs within a molecule determines the net effect on its biological properties. Understanding these effects is crucial for medicinal chemistry, where researchers aim to design drugs with optimal potency, selectivity, and pharmacokinetic properties.

### Analysis of physicochemical and pharmacokinetic properties

To determine compliance with Lipinski and Veber's criteria, it is essential to evaluate the physicochemical characteristics of 3-formyl chromone derivatives (**1**–**16**). Before a chemical can be administered orally, it must meet the five criteria as per Lipinski's recommendations: (a) molecular weight (MW) of 500 g/mol or less; (b) octanol–water partition coefficient (log P ≤ 5); (c) no more than five H-bond donors (HBD); no more than ten H-bond acceptors (HBA); and (e) no more than 140 Å^2^ of topological polar surface area (TPSA)^66,67^. Additionally, Veber has proposed two more requirements for drug bioavailability: (a) TPSA must be less than or equal to 140 Å^2^ (as per Lipinski's standards), and (b) the number of rotatable bonds (nrotb) in the molecules should be less than 10. The compounds (**1**–**16**) with the most promising biological activity were analyzed using SwissADME to assess their compliance with the Lipinski and Veber criteria. All compounds (**1**–**16**) were found to meet the prescribed limit ranges specified by the Lipinski and Veber criteria (Table 3). Additionally, there was a strong agreement (less than 6) for bioactive compounds regarding molecular weight (< 500 g/mol), MLOGP (4.15), and Log S (ESOL) within the specified limit ranges. Moreover, all compounds (**1**–**16**) that obtained a drug-like (bioavailability) score of **1** demonstrated their adherence to the criteria, providing robust theoretical support for developing innovative novel drugs.

**Table 3:** In silico prediction of physicochemical parameters for the derivatives of 3-formyl chromone (**1** − **16**).

Ligand	MW	LogP	HBA	HBD	nroth	TPSA (Å^2^)
Lipinski*	≤ 500	≤ 5	≤ 5	≤ 10	≤ 5	-
Veber**	-	-	-	-	≤ 10	≤ 140
1	174.15	1.46	3	0	1	47.28
2	188.18	1.79	3	0	1	47.28
3	202.21	2.09	3	0	2	47.28
4	216.23	2.4	3	0	2	47.28
5	190.15	1.03	4	1	1	67.51
6	204.18	1.44	4	0	2	56.51
7	218.21	1.78	4	0	3	56.51
8	199.16	0.89	4	0	1	71.07
9	192.14	1.76	4	0	1	47.28
10	208.60	1.98	3	0	1	47.28
11	253.05	2.08	3	0	1	47.28
12	189.17	1.2	3	1	1	73.30
13	219.15	0.65	5	0	2	93.10
14	253.05	2.08	3	0	1	47.28
15	243.04	2.5	3	0	1	47.28
16	245.06	2.23	3	0	1	43.37
vitexin	432.38	− 0.02	10	7	3	181.05
myricetin	318.24	0.79	8	6	1	151.59
D	408.87	2.17	6	4	6	99.38

*Lipinski reference values; **Veber reference values; MW, molecular weight; LogP, lipophilicity (O/W); HBD, number of hydrogen bond donors; HBA, number of hydrogen bond acceptors; nroth, Number of rotatable bonds;TPSA, topological polar surface area (Å^2^). D (Reference drug: Dapagliflozin).

The drug-likeness parameters calculated using molinspiration for all **16** compounds are presented in Table 4, which includes their potential as G protein-coupled receptor (GPCR) ligands, ion channel modulators (ICM), kinase inhibitors (KI), nuclear receptor ligands (NRL), protease inhibitors (PI), and enzyme inhibitors (EI). Among these, compound **4** showed a GPCR value of − 0.66, and compared to 0.15 for the reference drug D (dapagliflozin).

**Table 4:** Drug-likeness assessment of 3-formyl chromone derivatives (**1**–**16**) by molinspiration

Ligand	GPCR	ICM	KI	NRL	PI	EI
**1**	− 1.03	− 0.88	− 1.06	− 0.91	− 1.64	− 0.36
**2**	− 0.97	− 0.94	− 1.01	− 0.81	− 1.56	− 0.39
**3**	− 0.75	− 0.70	− 0.90	− 0.57	− 1.28	− 0.21
**4**	− 0.66	− 0.66	− 0.74	− 0.43	− 1.17	− 0.16
**5**	− 0.81	− 0.70	− 0.79	− 0.48	− 1.46	− 0.17
**6**	− 0.84	− 0.82	− 0.80	− 0.62	− 1.42	− 0.28
**7**	− 0.78	− 0.77	− 0.77	− 0.48	− 1.30	− 0.27
**8**	− 0.74	− 0.72	− 0.64	− 0.47	− 1.26	− 0.15
**9**	− 0.85	− 0.80	− 0.86	− 0.70	− 1.52	− 0.29
**10**	− 0.91	− 0.79	− 0.94	− 0.80	− 1.51	− 0.34
**11**	− 1.13	− 0.98	− 1.00	− 1.04	− 1.71	− 0.45
**12**	− 0.81	− 0.67	− 0.68	− 0.86	− 1.31	− 0.12
**13**	− 0.89	− 0.70	− 0.84	− 0.68	− 1.38	− 0.33
**14**	− 1.21	− 0.92	− 1.12	− 1.10	− 1.82	− 0.46
**15**	− 0.91	− 0.89	− 0.87	− 0.67	− 1.33	− 0.25
**16**	− 0.74	− 0.61	− 0.89	− 0.59	− 1.33	− 0.22
**Vitexin**	0.13	− 0.14	0.19	0.23	0.03	0.46
**Myricetin**	− 0.06	− 0.18	0.28	0.32	− 0.20	0.30
D	0.15	− 0.07	− 0.05	0.09	0.06	0.25

D (Reference drug: Dapagliflozin).

In drug development, it is crucial to assess the pharmacokinetic properties, such as absorption, distribution, metabolism, excretion, and toxicity (ADMET), to ensure innovative drugs' efficient and economical creation. This study utilized SwissADME (http://www.swissadme.ch/index.php) and admetSAR (http://lmmd.ecust.edu.cn/admetsar2/) software to evaluate the ADMET properties of all **16** compounds (**1**–**16**). The evaluation involved seven essential ADMET characteristics, as listed in Table 5, including cytochrome P450 enzymes (CYP3A4 and CYP2C19) inhibition, hERG inhibition, plasma protein binding (PPB), blood–brain barrier (BBB) penetration, human intestinal absorption (HIA), and synthetic accessibility (SA) score. It should be noted that drugs that affect the central nervous system (CNS) should have good blood–brain barrier penetration, whereas those that do should not penetrate the BBB. Low absorption is defined as < 0.1, medium absorption as 0.1–2, and high BBB penetration as > 2^67^. The results indicate that all compounds (**1**–**16**) meet the ADMET standards for drug-likeness (bioavailability), promising for developing novel drugs.

**Table 5:** In silico prediction of selected ADMET parameters for the 3-formyl chromone derivatives (**1**–**16**).

Ligand	^b^HIA	^b^BBB	^b^PPB	^b^CYP3A4 inhibition	^b^CYP2C19 inhibition	^b^hERG_pIC50	^c^Synthetic Accessibility score
1	0.9939	− 0.6250	0.751	− 0.7657	0.5874	− 0.7838	2.43
2	0.9952	− 0.5750	0.846	− 0.7427	− 0.6462	− 0.7722	2.51
3	0.9950	− 0.5750	0.723	− 0.8213	0.6574	− 0.5211	2.52
4	0.9948	− 0.5	0.921	0.8012	0.6092	− 0.4744	2.62
5	0.9815	− 0.8500	0.769	− 0.7920	− 0.6630	− 0.8597	2.34
6	0.9909	− 0.6500	0.791	− 0.6403	0.6478	− 0.6015	2.43
7	0.9930	− 0.6750	0.716	− 0.8523	0.9446	− 0.5680	2.5
8	0.9944	0.5500	0.754	0.5078	0.5218	− 0.7564	2.3
9	0.9947	− 0.5750	0.787	− 0.7213	0.5464	− 0.7399	2.34
10	0.9944	− 0.5500	0.874	− 0.6170	0.6182	− 0.8247	2.34
11	0.9932	− 0.5750	0.879	− 0.5316	0.5415	− 0.7425	2.46
12	0.9938	− 0.6000	0.839	0.5140	− 0.5565	− 0.6906	2.66
13	0.9691	− 0.5250	0.72	− 0.6990	− 0.6065	− 0.9129	2.51
14	0.9932	− 0.5750	0.815	− 0.5316	0.5415	− 0.7636	2.55
15	0.9944	− 0.5500	0.839	− 0.6170	0.6182	− 0.7854	2.46
16	0.9944	− 0.5500	0.806	− 0.6170	0.6182	− 0.8011	2.60
Vitexin	0.6665	− 0.7000	0.845	− 0.8310	− 0.9240	− 0.4762	5.12
Myricetin	0.9071	− 0.7750	0.991	0.6951	− 0.9025	− 0.7812	3.27
D	0.6268	0.6250	0.736	− 0.8763	0.5166	0.7719	4.52

^a^HIA: Human Intestinal Absorption (%); BBB: Blood–Brain Barrier penetration; PPB: plasma protein binding; CYP3A4: Cytochrome P4503A4; CYP2C19: Cytochrome P4502C19; hERG: human ether-a-go-go-related gene, hERG inhibition potential (pIC_50_), the potential risk for inhibitors ranges 5.5–6.

^b^The values are using admetSAR.^c^The values are using swissADME. D (Reference drug: Dapagliflozin).

Based on our study, most compounds showed BBB penetration values between − 0.5 and − 0.7, similar to vitexin (− 0.70) and myricetin (− 0.77). The range of pIC50 predictions for potential risk from hERG activity inhibitors is between − 0.4 and − 0.82^68^. Our research showed that compound **4** had the lowest hERG pIC50 value of − 0.4744, while all other compounds had values below the reference range. To assess the synthetic accessibility (SA) score of the drug-like compounds (**1**–**16**), which ranges from 1 (very simple) to 10 (very difficult), we employed a unique method^64^. The SA scores of all the compounds (**1**–**16**) ranged from 2.4 to 2.6, which was lower than the reference drug, with a high SA score of 4.52. Vitexin and myricetin have higher SA scores than compounds (**1–16**). Our findings suggest that all compounds (**1**–**16**) meet the ADMET standards, indicating drug-likeness (bioavailability) and potential for developing novel medications.

### Analysis of pharmacological activities

For a comprehensive investigation of the potential pharmacological effects of the 3-formyl chromone derivatives (**1**–**16**), Multilevel Neighborhoods of Atoms (MNA) descriptors were applied using the Prediction of Activity Spectra for Substances (PASS) method. The use of MNA descriptors allowed for a unique and detailed characterization of the chemical structures, which aided in clarifying the compounds' potential biological functions. The PASS method can simultaneously predict various biological activities, such as mutagenicity, carcinogenicity, teratogenicity, embryotoxicity, and primary and side pharmacological effects. A compound's biological activity is influenced by its structural and physicochemical characteristics, the biological entity (such as species, gender, age, etc.), and the treatment approach (such as dose, route of administration, etc.). The MNA descriptors are used by PASS to determine the probable activity (Pa) and probable inactivity (Pi) for the anticipated activity spectrum of a drug. These probabilities range from 0.000 to 1.000, where Pa + Pi ≠ 1. PASS predictions can be interpreted in several ways. For example, if Pa > 0.7, there is a high likelihood of finding the activity experimentally. If Pa is between 0.5 and 0.7, the chemical is likely to demonstrate the activity in an experimental setting, but it is likely different from recognized pharmaceutical drugs. The likelihood of finding the activity experimentally is lower if Pa < 0.5.

3-formyl chromone derivatives (**1**–**16**), except some of them exhibited Pa values greater than 0.5, as presented in Table 6. The histidine kinase inhibitor values ranged from Pa = 0.614 to Pa = 0.830, nearly identical to those of the vitexin (Pa = 0.819) and myricetin (Pa = 0.892). But the reference drug, dapagliflozin, has a lower Pa (0.439) value as a histidine kinase inhibitor. Compound **4** had a Pa value of 0.669 and a Pi value of 0.010 as a histidine kinase inhibitor, indicating that it was good in promoting antibacterial activity by targeting bacterial histidine kinase, although more potent than dapagliflozin. Dysregulated kinase activity is frequently associated with multiple disorders, including cancer. Kinases are enzymes that are essential for many biological functions, including cell proliferation, differentiation, and signaling. Moreover, all compounds, except for compounds **9** and **13**, showed insulysin inhibitor values between Pa = 0.515 to Pa = 0.729, indicating that these compounds could potentially be therapeutic agents for diabetes treatment. Using a clearance mechanism anchored on degradation, IDE controls the amount of circulating insulin in several organs^69,70^. The reference drug shows no activity as an insulysin inhibitor. **HIF1α** expression is frequently increased in cancer cells and is linked to metastasis and tumor formation. Compound** 4** was found to have a Pa value of 0.738 and a Pi value of 0.016 as a **HIF1α** expression inhibitor, indicating moderate potency in inhibiting the expression of **HIF1α**. Additionally, some compounds showed stronger activity as alcohol dehydrogenase inhibitors than the reference drugs.

**Table 6:** Predicted biological activity of the 3-formyl chromone derivatives (**1** − **16**) using PASS.

Ligand	Alcohol Dehydrogenase (NADP +) inhibitor (Anti-microbial)		Apoptosis Agonist (Anti-cancer)		HIF1α expression inhibitor (Anti-tumor)		Insulysin inhibitor (Anti-diabetic)		Histidine kinase inhibitor (Anti-bacterial)	
	Pa	Pi	Pa	Pi	Pa	Pi	Pa	Pi	Pa	Pi
**1**	0.737	0.004	0.675	0.017	0.626	0.029	0.711	0.007	0.774	0.005
**2**	0.500	0.009	0.631	0.023	0.549	0.044	0.729	0.005	0.715	0.007
**3**	0.623	0.005	0.557	0.031	0.542	0.045	0.578	0.029	0.695	0.008
**4**	0.395	0.014	0.585	0.028	0.738	0.016	0.515	0.045	0.669	0.010
**5**	0.788	0.003	0.698	0.015	0.768	0.014	0.727	0.005	0.830	0.003
**6**	0.459	0.011	0.682	0.017	0.647	0.026	0.700	0.008	0.695	0.008
**7**	0.607	0.005	0.568	0.030	0.467	0.069	0.679	0.011	0.661	0.011
**8**	0.489	0.009	0.568	0.030	0.695	0.021	0.541	0.038	0.626	0.013
**9**	0.690	0.004	0.530	0.035	0.631	0.028	0.461	0.063	0.679	0.009
**10**	0.620	0.005	0.544	0.033	0.485	0.062	0.687	0.010	0.792	0.004
**11**	0.763	0.004	0.622	0.024	0.390	0.107	0.620	0.021	0.659	0.011
**12**	0.489	0.009	0.568	0.030	0.695	0.021	0.541	0.038	0.626	0.013
**13**	0.270	0.024	0.574	0.029	0.441	0.080	0.455	0.066	0.614	0.014
**14**	0.770	0.004	0.553	0.032	0.378	0.114	0.604	0.024	0.638	0.012
**15**	0.398	0.014	0.426	0.062	0.316	0.162	0.592	0.026	0.749	0.005
**16**	0.507	0.007	0.507	0.309	0.439	0.081	0.624	0.020	0.748	0.005
**Vitexin**	0.382	0.014	0.737	0.012	0.940	0.004	-	-	0.819	0.004
**Myricetin**	0.912	0.002	0.915	0.004	0.969	0.002	0.603	0.024	0.892	0.002
**D**	–	–	0.292	0.131	0.469	0.068	–	–	0.439	0.041

*D (Reference drug: dapagliflozin).

Our findings revealed that the Pa values of the studied compounds, acting as both insulin inhibitors (anti-diabetic) and alcohol dehydrogenase inhibitors, ranged from 0.5 to 0.7, with only a few exceptions. This indicates a strong potential for these compounds to inhibit both insulin and alcohol dehydrogenase enzymes effectively. Additionally, our compounds exhibited apoptosis agonist activity, which is well-known for inducing cancer cell death^71^.

### In silico molecular docking

Molecular docking investigation and in silico docking was performed on 3-formyl chromone compounds (**1**–**16**) and reference standards of vitexin to inhibit targeted proteins, including CAD (3TWO), BHK (3DGE), IDE (6BF8), HIF-α (2WA4), p53 (7EAX), COX (6Y3C), and Mpro of SARS-CoV2 (6LU7), as presented in Table 7. Results revealed that compound **4** had a strong binding affinity for IDE (− 8.5 kcal mol^−1^). Additionally, all docking values obtained were superior to the reference drug dapagliflozin (IDE: − 7.9 kcal mol^−1^), indicating their potential for diabetes treatment. Furthermore, compounds **1**–**13** showed good binding affinity, while compounds **14**, **15**, and **16**, which had additional changes in their chemical structure, exhibited weaker binding affinity. Although Vitexin has a lower binding affinity (− 8.3 kcal mol^−1^) than compound **4**, myricetin has a binding affinity (− 8.5 kcal mol^−1^) similar to compound **4**.

**Table 7:** Molecular docking simulation results for 3-formyl chromone derivatives (**1**–**16**) against seven targets.

Binding affinity (kcal mol^−1^)							
Ligand	CAD (3TWO)	IDE (6BF8)	P53 (7EAX)	BHK (3DGE)	HIF-α (2WA4)	Mpro (6LU7)	COX (6Y3C)
**1**	− 6.7	− 7.1	− 5.7	− 7.3	− 6.2	− 5.8	− 6.7
**2**	− 6.9	− 7.9	− 6.2	− 8.0	− 6.4	− 5.7	− 7.3
**3**	− 7.2	− 8.1	− 6.2	− 8.1	− 6.8	− 6.0	− 7.6
**4**	− 7.6	− 8.5	− 6.3	− 8.5	− 6.9	− 6.1	− 8.1
**5**	− 6.8	− 7.6	− 6.2	− 7.4	− 6.2	− 6.1	− 7.0
**6**	− 6.8	− 7.9	− 6.2	− 7.6	− 6.2	− 5.7	− 7.2
**7**	− 6.6	− 7.7	− 5.7	− 7.9	− 6.2	− 5.7	− 7.2
**8**	− 6.8	− 7.6	− 5.5	− 7.4	− 6.2	− 5.7	− 6.9
**9**	− 6.8	− 7.7	− 5.7	− 7.7	− 6.0	− 6.0	− 7.3
**10**	− 6.5	− 7.7	− 5.7	− 7.4	− 6.2	− 5.6	− 7.2
**11**	− 6.6	− 7.7	− 5.5	− 7.0	− 5.9	− 5.3	− 7.0
**12**	− 7.2	− 8.1	− 6.0	− 8.5	− 6.5	− 6.6	− 7.6
**13**	− 7.3	− 8.2	− 5.7	− 8.3	− 6.7	− 6.4	− 7.7
**14**	− 6.3	− 7.2	− 5.7	− 7.7	− 5.9	− 5.9	− 6.1
**15**	− 6.4	− 6.7	− 5.6	− 8.0	− 6.4	− 5.8	− 6.1
**16**	− 6.5	− 7.6	− 5.9	− 7.5	− 6.0	− 6.2	− 7.6
**Vitexin**	− 7.7	− 8.3	− 8.1	− 9.8	− 7.7	− 7.9	− 8.9
**Myricetin**	− 7.9	− 8.5	− 7.4	− 8.2	− 7.9	− 7.4	− 8.6
**D**	− 8.0	− 7.9	− 7.2	− 8.9	− 7.4	− 7.1	− 7.1

The findings of the molecular docking study indicated that compounds **1**–**16** exhibited a higher binding affinity for IDE, BHK, and COX, while their binding affinity for human CAD, p53, Mpro, and HIF-α was relatively lower. These results suggest that these compounds have the potential to be used as therapeutic agents for diseases related to the dysregulation of IDE, BHK, and COX. While molecular docking is a valuable tool for drug discovery, it is crucial to consider a compound's pharmacokinetics, toxicity, and metabolism, in addition to its binding affinity. In vitro and in vivo research is necessary to comprehend how compounds behave in living organisms and evaluate their safety and efficacy. By employing a combination of computational and experimental techniques, researchers can optimize the compounds and create dependable and effective therapeutic drugs.

D (Reference drug: dapagliflozin).

**Figure 3:**
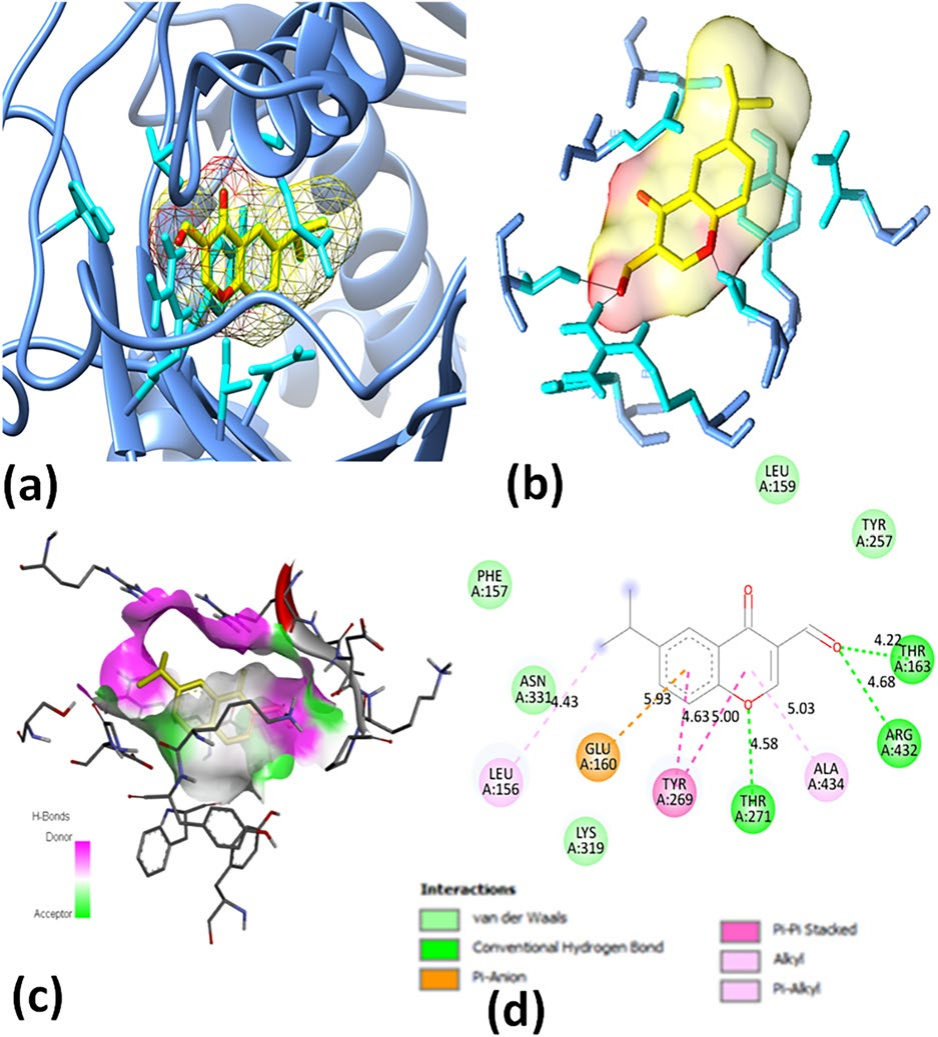
Molecular docking poses: (**a**) Ligand in protein pocket; (**b**) Active site; (**c**) Hydrogen bonding in solid; (**d**) Ligand–protein interaction for 2D diagram of compound **4** and modeled protein (IDE).

**Figure 4:**
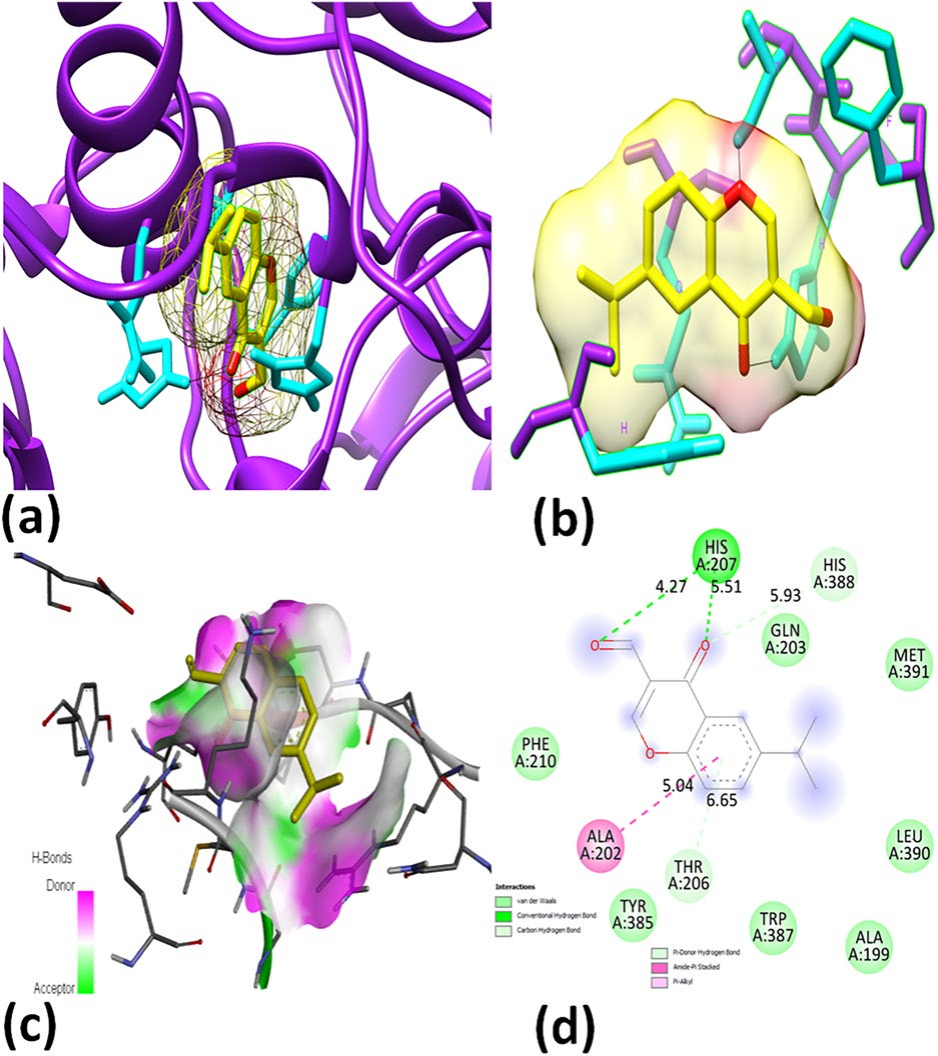
Molecular docking poses: (**a**) Ligand in protein pocket; (**b**) Active site; (**c**) Hydrogen bonding in solid; (**d**) Ligand–protein interaction for 2D diagram of compound **4** and modeled protein (COX).

Furthermore, Figs. 3 and 4 present 2D diagrams for compound **4** in IDE and COX, depicting the ligand's interactions with the protein and how it affects the pathogens' active components. The bond lengths and residue numbers are indicated in Fig. 3d and Fig. 4d; the ligand–protein interaction of compound **4** through the amino acid residues of IDE is demonstrated, revealing around eight different bonds. Four of these bonds are hydrophobic, including Pi–Pi stacking on residue TYR A: 269, Pi-alkyl on residue LEU A: 156, and alkyl on residue ALA A: 434. Three conventional hydrogen bonds are at residues THR A: 271, ARG A: 432, and THR A: 163. The last bond is Pi-anion on residue GLU A: 160. The protein–ligand interaction of compound **4** in COX, shown in Fig. 4, exhibits five different interactions. Four of them are hydrogen bonds, including a conventional hydrogen bond at residue HIS A: 207, a carbon-hydrogen bond with THR A: 206, and a Pi-donor hydrogen bond with HIS A: 388. One bond is hydrophobic, including amide-Pi stacking with ALA A: 202.

Based on the results of molecular docking and interaction analysis, it has been concluded that compound **4** exhibits a stronger binding affinity towards the IDE protein. Additionally, all compounds also show good interactions with the IDE protein. The 2D diagram and bond interactions of the IDE protein with all compounds are shown in SI (Table S2). Thus, the in silico study suggests that compound** 4** has promising potential as an insulin-degrading enzyme protein inhibitor. The selection of Insulin Degrading Enzyme (IDE) as the target protein for our in-silico study of potential anti-diabetic agents is well-supported by existing research. IDE plays a critical role in insulin degradation, and its inhibition has been demonstrated as a promising strategy for improving blood sugar control in diabetic patients. This is further evidenced by the existence of previous in vivo and in vitro studies exploring the therapeutic potential of IDE inhibitors^17^. Our study aimed to leverage computational methods to identify novel candidate molecules that could target IDE and potentially offer new avenues for developing anti-diabetic drugs.

### In silico molecular dynamics

The goal of the MD simulations was to examine the stability and interactions of compound **4** with the IDE and COX proteins for a duration of 20 ns. The molecular docking analysis had previously shown that compound **4** had a strong binding affinity of − 8.5 kcal mol^−1^ with the IDE protein and a favorable binding affinity of − 8.1 kcal mol^−1^ with COX, indicating its potential as a drug candidate. The MD simulation results were evaluated using RMSD (Root Mean Square Deviation), RMSF (Root Mean Square Fluctuation), and Rg values, potential energies, temperature, and hydrogen bonding to gain a comprehensive understanding of the system's behavior over time.

**Figure 5:**
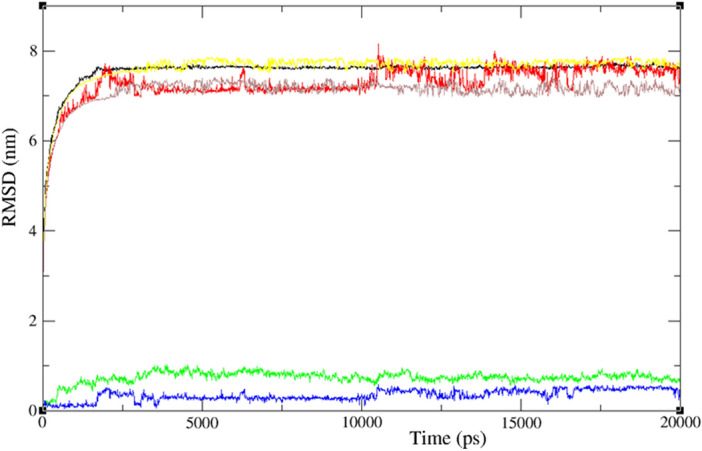
RMSD evolution for merge docked protein–ligand complex between two proteins and modeled ligand **4**, (IDE (6BF8), green line and COX (6Y3C), (**4**, red and black), water and ions in the ligand-IDE protein complex (yellow) and water and ions in the ligand-COX protein complex (brown) during 20 ns MD simulation.

The RMSD values derived from the MD simulations offer insights into ligand–protein complexes' stability and conformational changes, as presented in Fig. 5. For the ligand-IDE protein complex (green curve), the RMSD values were in the range of 0.2 to 0.5 nm, while for the ligand-COX protein complex (blue curve), the values were between 0.1 and 0.2 nm, indicating that both complexes are stable and exhibit minimal deviation from their initial positions during the simulation. However, the RMSD values for water and ions in the ligand-IDE protein complex (yellow curve) were approximately 7.7 nm, while those in the ligand-COX protein complex (brown curve) were about 7.3 nm. These observations suggest potential differences in the stability and dynamics of the solvent molecules in the two complexes, which could be due to the size and shape of the binding sites, as well as the specificity and strength of ligand–protein interactions. It is important to note that the RMSD values for water and ions can be affected by various simulation parameters, including force field, simulation time, and binding site definition. Therefore, further analyses, such as solvent density profiles, hydrogen bonding patterns, and residence times, are recommended to elucidate the underlying factors contributing to the differences in RMSD values.

**Figure 6:**
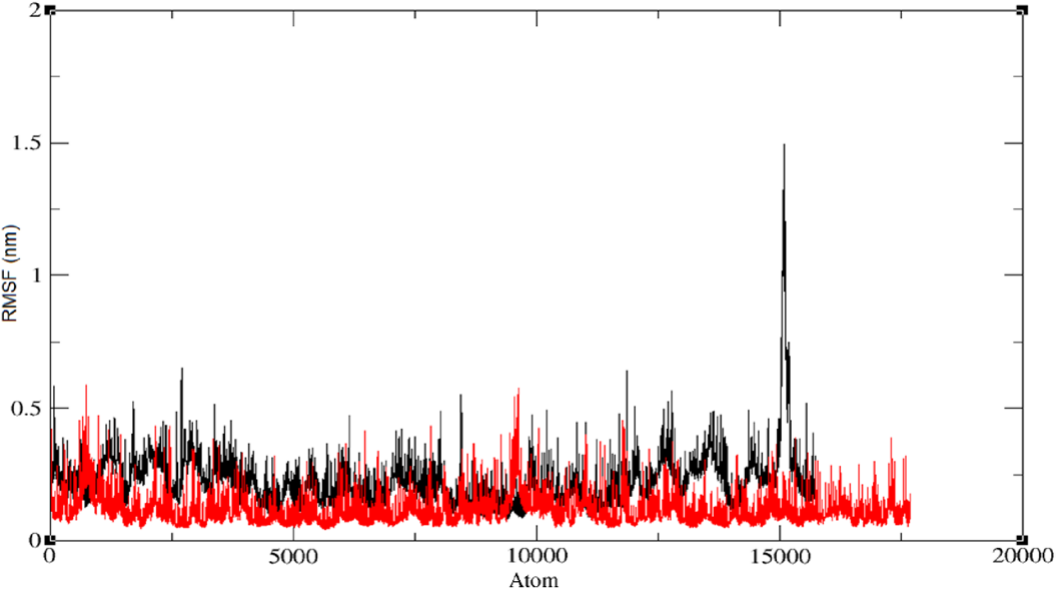
RMSF evolution for the IDE protein–ligand complex (black) and the COX protein–ligand complex (red) and modeled ligand **4**, during 20 ns MD simulation.

The RMSF is an important measure of the flexibility and mobility of protein–ligand complexes in molecular dynamics simulations. In this study, separate RMSF values were calculated for the protein and ligand in the IDE (6BF8) and COX (6Y3C) protein–ligand complexes with compound **4**, and the results are shown in Fig. 6. A lower RMSF value indicates greater rigidity of a particular residue or atom, whereas a higher RMSF value indicates greater flexibility or mobility. The COX protein–ligand complex had an RMSF value of 0.1 nm (red line), indicating that it is relatively rigidly held in place, while the IDE protein–ligand complex had an RMSF value of 0.3 nm (black line), indicating that it is more flexible. A fluctuation observed in the IDE protein–ligand complex at the 15,000 range amino acid atom with a value of around 1.5 nm could be due to a number of factors, such as the inherent flexibility of the amino acid or its interaction with the ligand or solvent molecules. Additional analysis, such as examining the specific interactions of this residue with other parts of the protein or ligand, may provide further insights into the cause of this fluctuation. The differences in RMSF values between the COX and IDE protein–ligand complexes suggest that they have varying degrees of flexibility or mobility, which may have implications for their biological function. The fluctuation observed in the IDE protein–ligand complex at atom 15,000 highlights the importance of careful analysis of simulation results and emphasizes the potential complexity of protein–ligand dynamics.

**Figure 7:**
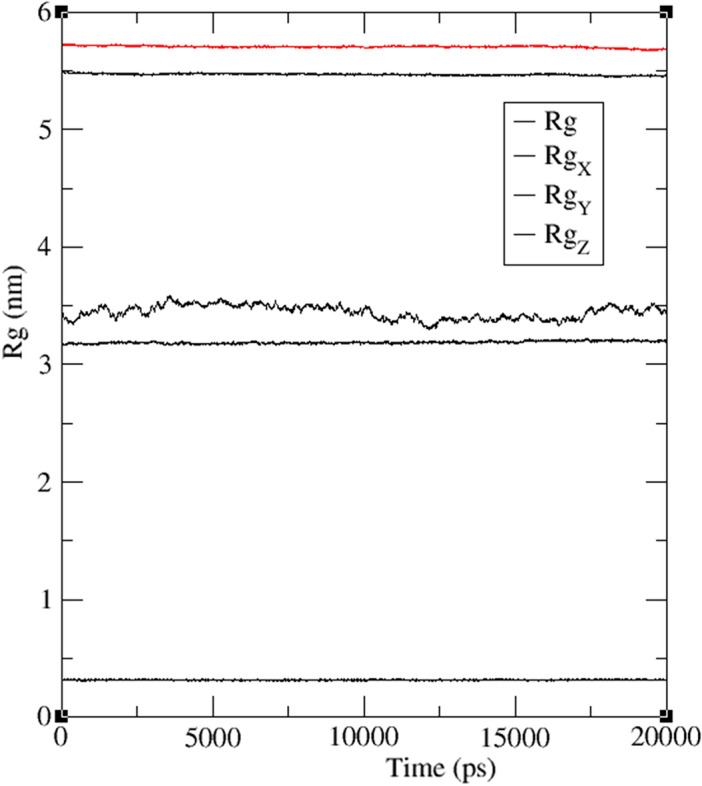
Radius of gyration, Rg (nm) versus time (ps) plots of the ligand (R_g_), IDE protein–ligand complex (Rg_X_), COX protein–ligand complex (Rg_Y_) and water-ions (Rg_Z,_) with modeled ligand **4**, during 20 ns MD simulation.

The radius of gyration (Rg) is a measure of molecular compactness and is often used to monitor conformational changes in MD simulations. In this study, the Rg values for compound **4** bound to the IDE and COX proteins, were compared over a 20 ns simulation period, as shown in Fig. 7. It was observed that the Rg values for the IDE (protein–ligand) complex exhibited slight fluctuations within the range of 3.4–3.5 nm, while the Rg value for the COX complex remained consistently at 3.1 nm throughout the simulation period. This suggests that the COX complex is more stable and less prone to conformational changes compared to the IDE complex. Additionally, the Rg value for the ligand alone was found to be 0.3 nm in both cases, indicating that the ligand assumes a more compact conformation when bound to the proteins. Furthermore, the Rg value for the water-ion was larger than that of the protein–ligand complex, indicating a more loosely packed structure for water-ions compared to the ligand–protein complex. Specifically, the Rg values for the water-ion were 5.72 nm (red line) and 5.485 nm (black line) in the COX and IDE protein–ligand complexes, respectively, over the simulation period. Understanding the behavior of protein–ligand complexes can be valuable for the rational design of drugs with improved binding affinity and specificity.

**Figure 8:**
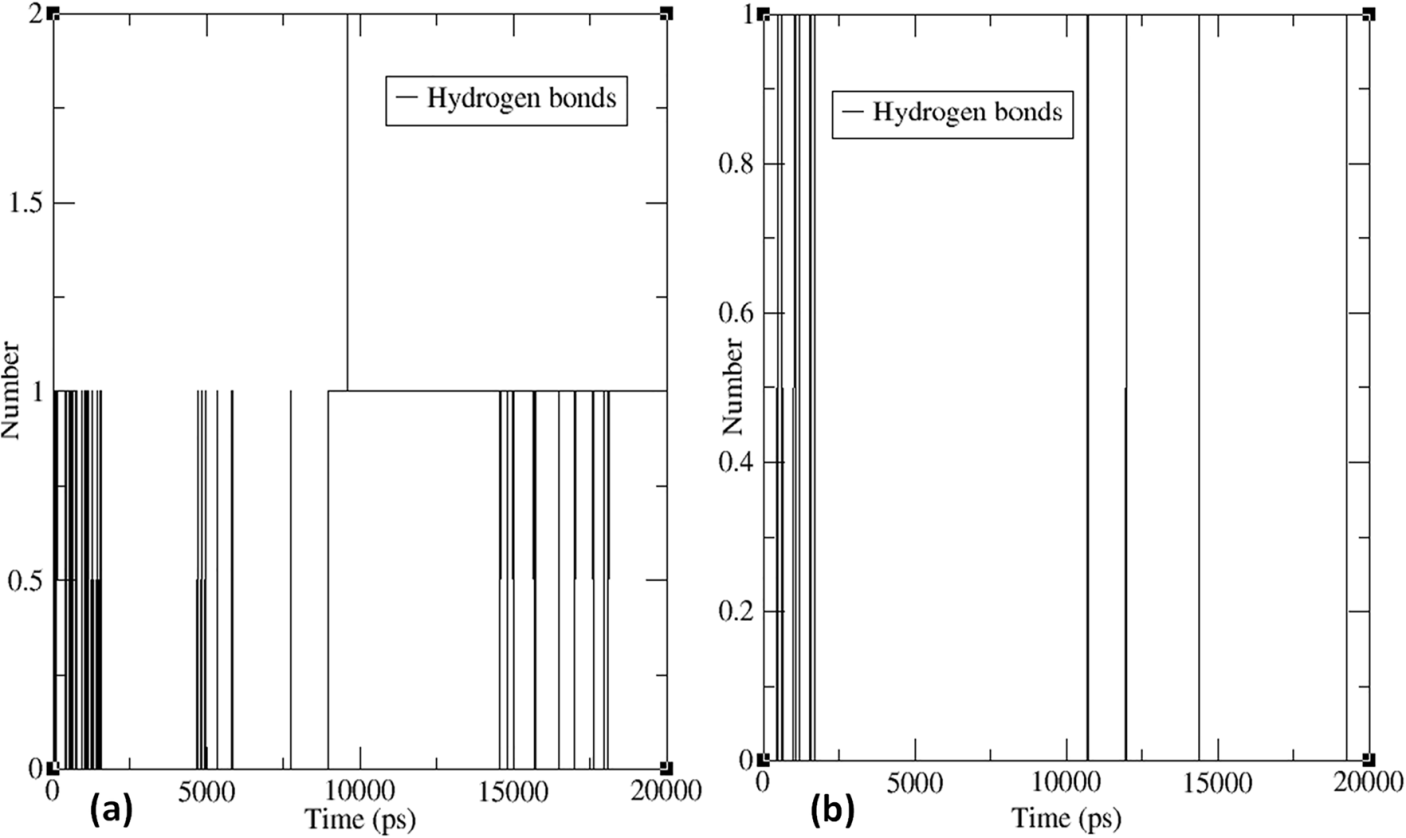
Plots of the number of hydrogen bond versus time (ps) for (**a**) between protein complex of PDB: 6BF8 and compound **4** and (**b**) between protein complex of PDB: 6Y3C and compound **4** during 20 ns MD simulation.

Hydrogen bonds play a crucial role in protein–ligand interactions, providing valuable information about binding strength and specificity. In this study, MD simulations were used to investigate the number of hydrogen bonds formed between compound **4** and the active site of two proteins, IDE and COX, as shown in Fig. 8.

The number of hydrogen bonds between the ligand and the active site of the IDE protein fluctuated between 0 and 2 during the 20 ns simulation period (Fig. 8a). This suggests that hydrogen bond formation between the ligand and the IDE protein needs to be consistently maintained and is influenced by many factors. Consequently, the IDE protein and ligand interaction is dynamic rather than static. On the other hand, the number of hydrogen bonds formed between the ligand and the active site of the COX protein ranged from 0 to 1 throughout the 20 ns simulation (Fig. 8b). This indicates that the hydrogen bond formation between the COX protein and the ligand is relatively stable and consistent, implying a more static and less dynamic interaction compared to the IDE protein–ligand interaction.

These findings demonstrate that multiple factors, including conformational changes, ligand movement, and the specific characteristics of the protein, influence the number of hydrogen bonds formed between a protein and a ligand. The observed dynamic nature of hydrogen bond formation in the IDE protein–ligand interaction holds significant implications for drug design and optimization. It emphasizes the importance of considering the dynamic aspects of protein–ligand interactions in rational drug design processes.

**Figure 9:**
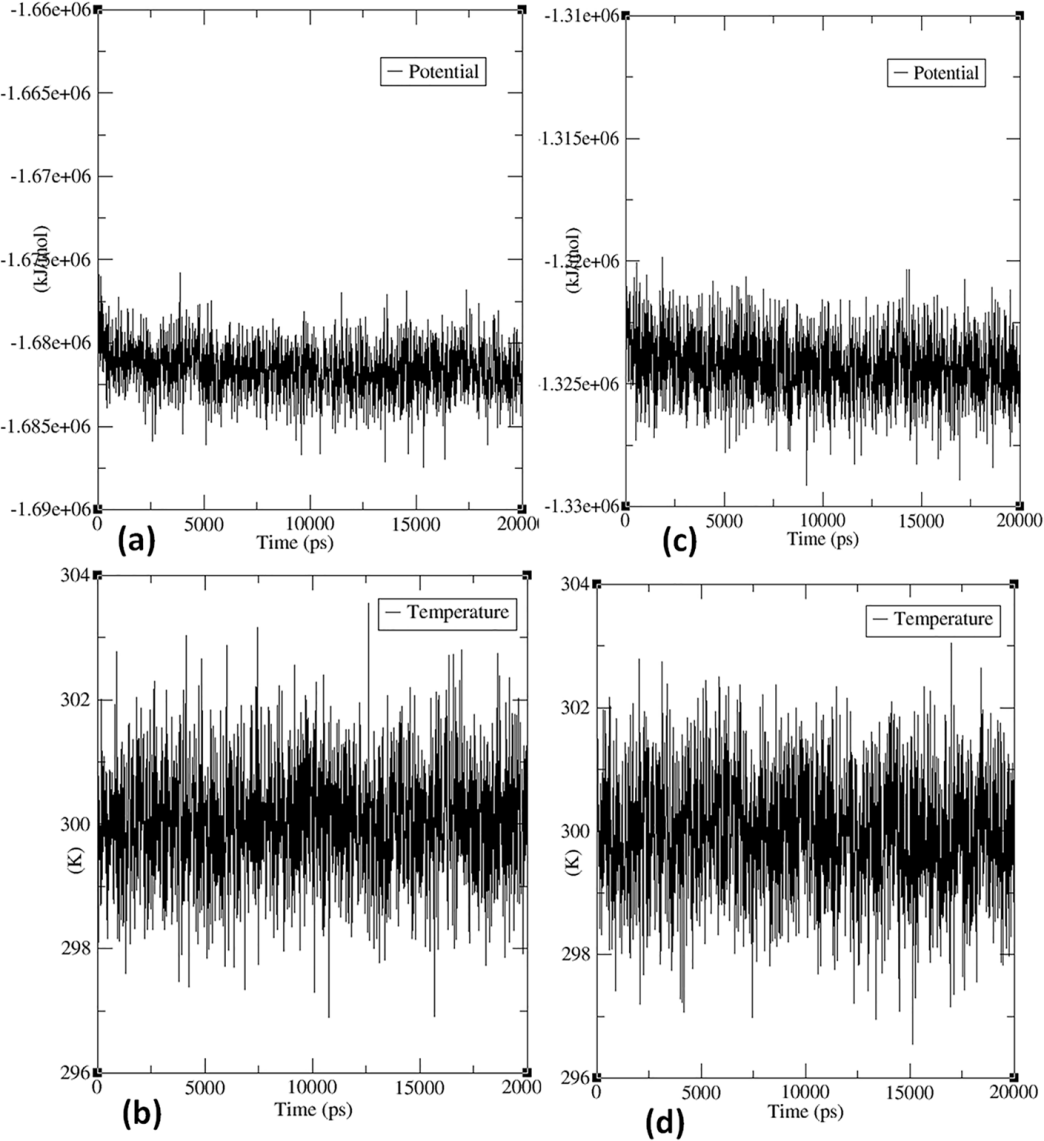
The temperature and potential energy curves over the course of the 20 ns MD simulation for the potential energy graph for the protein–ligand complex of IDE (**a**) and COX (**c**); the temperature graph of the protein–ligand complex of IDE (**b**) and COX (**d**) with compound **4.**

In MD simulations, monitoring the temperature of the system is crucial to ensure its stability. In this study, the IDE and COX proteins' temperature was relatively stable, fluctuating between 298 and 302 Kelvin during the 20,000 ps simulation period (Fig. 9a,c). This indicates that the simulation was well-controlled and the system remained within an appropriate temperature range. However, the system's potential energy, which reflects the interactions between atoms, showed fluctuations throughout the simulation period. For the IDE protein, the potential energy fluctuated between − 1.680e^+06^ and − 1.677e^+06^ kJ mol^−1^ (Fig. 9b), while for the COX protein, it varied between − 1.325e^+06^ and − 1.323e^+06^ kJ mol^−1^ (Fig. 9d). These fluctuations imply that the interactions between the atoms in the system are dynamic and continuously changing. In summary, the stable temperature observed in the study indicates a well-controlled simulation, but the fluctuation in potential energy highlights the dynamic nature of protein–ligand interactions.

### PCA analysis

**Figure 10:**
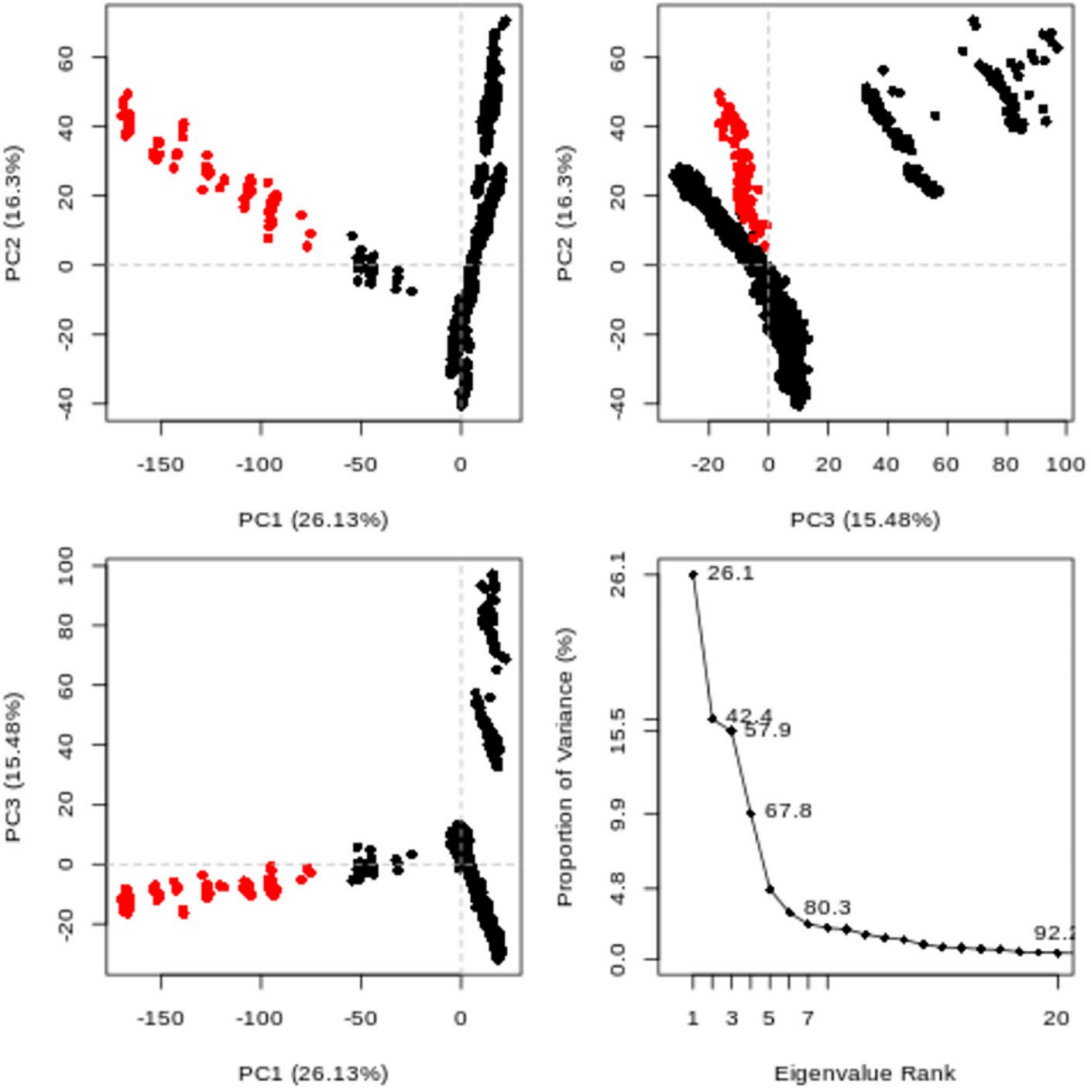
PCA of the IDE complex with compound **4** at 300 K MD trajectory, where red dots represent stable conformations and black dots indicate energetically unstable conformations.

Conformational principal component analysis (PCA) was performed on the simulated molecular dynamics (MD) trajectories of the IDE protein–ligand complex with compound **4** at 300 K. The aim was to identify the variability, collective motions, and changes in protein conformations observed in subsets of the primary components throughout the MD simulations. The PCA analysis was conducted using the Bio3D program^55–57^. The resulting eigenvalues versus eigenvector plots are displayed in Fig. 10. The first three eigenvectors, namely PC1, PC2, and PC3, were utilized to compare the dominant motions within the smaller trajectory subgroup. Colored dots represent the captured variance by the eigenvectors. Regarding the internal motions observed in the MD trajectory, the protein–ligand complex simulation at 300 K exhibited the most extensive variability in PC1, accounting for 26.13% of the total variance. PC2 showed a lower percentage of variation (16.3%), and PC3 showed 15.48% of the conflict. These three components accounted for 57.9% of the total variance (Fig. 10).

Furthermore, the simulation appeared to have converged, as evidenced by the cosine content value of the eigenvectors, which was computed to be 0.32 based on the MD trajectory. The conformational PCA analysis provided insights into the dominant motions and variability within the IDE protein–ligand complex during the MD simulation at 300 K. These findings contribute to a better understanding of the dynamic behavior and conformational changes occurring in the protein–ligand complex.

## Conclusion

This study employed computational methods to virtually screen a library of 6-substituted 3-formyl chromone derivatives (compounds **1–16**) for potential therapeutic applications. The results revealed promising anti-diabetic activity, particularly for compound **4**, demonstrating a strong binding affinity to a crucial enzyme involved in diabetes management (**IDE**). Notably, compound **4**'s docking score to **IDE** (-8.5 kcal mol^-1^) was comparable to the established medication dapagliflozin (-7.9 kcal mol^-1^). Additionally, all compounds adhered to Lipinski and Veber's rules, indicating good oral bioavailability and potentially lower toxicity. These findings suggest that compound **4** represents a significant step forward in the discovery of novel anti-diabetic therapies. The selection of Insulin Degrading Enzyme (IDE) as the target protein for our in-silico study of potential anti-diabetic agents is well-supported by existing research. IDE plays a critical role in insulin degradation, and its inhibition has been demonstrated as a promising strategy for improving blood sugar control in diabetic patients. This is further evidenced by the existence of previous in vivo and in vitro studies exploring the therapeutic potential of IDE inhibitors. Future investigations will involve in vitro and in vivo experiments to validate the computational predictions and elucidate the compound's mechanism of action. This research opens new avenues for developing more effective and potentially less toxic treatments for diabetes.

### Supplementary Information

Supplementary Information.

## Data Availability

Here is a list of software and websites, including AdmetSAR, available at http://lmmd.ecust.edu.cn/admetsar2/, SwissADME accessible at http://www.swissadme.ch/, Pass prediction found at http://www.way2drug.com/passonline/, as well as coordinates of stable local minimum structures included in the supporting information. All data generated or analyzed during this study are included in supplementary information SI.
