# Supplementary material for: Investigating the potential of 6-substituted 3-formyl chromone derivatives as anti-diabetic agents using in silico methods
Source: Sci Rep. 2024 Jun 8;14:13221. doi: 10.1038/s41598-024-63237-y (PMC11162442; doi:10.1038/s41598-024-63237-y)
Supplement: Supplementary file 1 — Supplementary Information. [file 41598_2024_63237_MOESM1_ESM.docx]

**Investigating the Potential of 6-Substituted 3-Formyl Chromone Derivatives as Anti-Diabetic Agents using *In Silico* Methods**

**Minhaz Zabin Saif^1^, Nusrat Jahan Ikbal Esha^1^, Syeda Tasnim Quayum^1^, Shofiur Rahman^2,^*, Mahmoud A. Al-Gawati^2^, Ghadah Alsowygh^2^, Hamad Albrithen^2,3^, Abdullah Alodhayb^2,3^ Raymond A. Poirier^4,^* & Kabir M. Uddin^1,^***

^1^Department of Biochemistry and Microbiology, North South University, Bashundhara

Dhaka-1217, Bangladesh

^2^Biological and Environmental Sensing Research Unit, King Abdullah Institute for

Nanotechnology, King Saud University,

Riyadh 11451, Saudi Arabia

^3^Research Chair for Tribology, Surface, and Interface Sciences,

Department of Physics and Astronomy, College of Science,

King Saud University, Riyadh 11451, Saudi Arabia

^4^Department of Chemistry, Memorial University,

St. John’s, Newfoundland, Canada A1B 3X7

Tel.: +8801796585904

Fax: +8802-55668202

E-mail: [mohammed.uddin11@northsouth.edu](mailto:mohammed.uddin11@northsouth.edu);

[kabirmuddin@gmail.com](mailto:kabirmuddin@gmail.com)

[rpoirier@mun.ca](mailto:rpoirier@mun.ca)

[mrahman1@ksu.edu.sa](mailto:mrahman1@ksu.edu.sa)

[aalodhayb@ksu.edu.sa](mailto:aalodhayb@ksu.edu.sa)

_____________________

**Supplementary Table S1** Optimized structure for 3-Formylchromone (**1**) and Cartesian Z-matrix.

| **3-Formylchromone (1)** | | | **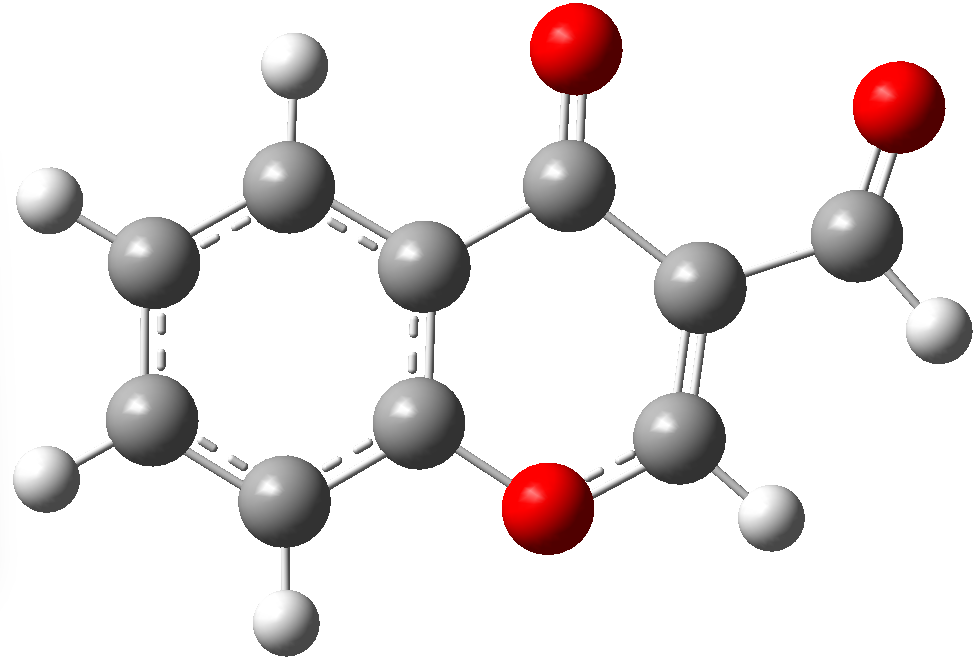** | | |
| --- | --- | --- | --- | --- | --- |
| Center  Number | Atom | Standard orientation: Coordinates (Angstroms) | | | |
|  |  | X | | Y | Z |
| 1 | O | -0.19739 | | 1.904419 | 0.000019 |
| 2 | O | 1.218799 | | -1.953924 | 0.000093 |
| 3 | O | 3.84733 | | -0.63763 | -0.000157 |
| 4 | C | -0.642916 | | -0.479051 | -0.000117 |
| 5 | C | -1.088953 | | 0.847354 | -0.000013 |
| 6 | C | 0.810509 | | -0.802065 | -0.00034 |
| 7 | C | -1.605098 | | -1.501316 | -0.000082 |
| 8 | C | 1.677006 | | 0.399723 | -0.000014 |
| 9 | C | -2.445002 | | 1.175719 | 0.000109 |
| 10 | C | 1.113641 | | 1.636082 | 0.000035 |
| 11 | C | -2.958471 | | -1.196048 | 0.00004 |
| 12 | C | -3.37627 | | 0.144164 | 0.000131 |
| 13 | C | 3.156663 | | 0.356981 | 0.000234 |
| 14 | H | -1.245362 | | -2.524793 | -0.000177 |
| 15 | H | -2.74099 | | 2.218937 | 0.00019 |
| 16 | H | 1.700228 | | 2.550644 | 0.000169 |
| 17 | H | -3.696264 | | -1.99191 | 0.000061 |
| 18 | H | -4.435384 | | 0.382275 | 0.000228 |
| 19 | H | 3.621218 | | 1.372673 | -0.000003 |

**Supplementary Table S2.** Optimized structure for 3-Formyl-6-methylchromone (**2**) and cartesian Z-matrix.

| **3-Formyl-6-methylchromone (2)** | | | 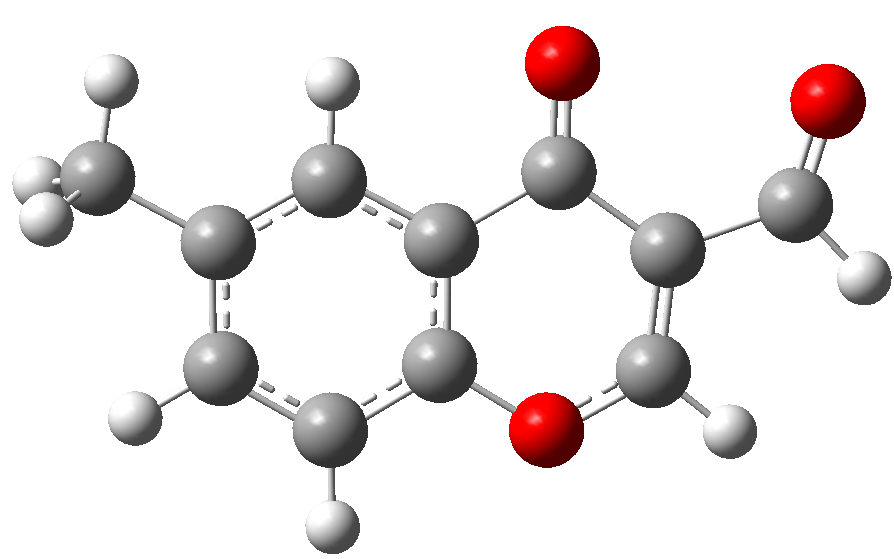 | | |
| --- | --- | --- | --- | --- | --- |
| Center  Number | Atom | Standard orientation: Coordinates (Angstroms) | | | |
|  |  | X | | Y | Z |
| 1 | O | -0.520774 | | 2.049986 | -0.000005 |
| 2 | O | -1.214968 | | -2.00198 | -0.000102 |
| 3 | O | -4.037496 | | -1.182659 | 0.00012 |
| 4 | C | 0.349702 | | -0.21411 | -0.000026 |
| 5 | C | 0.545718 | | 1.169239 | -0.000006 |
| 6 | C | 2.769933 | | -0.523687 | 0.000002 |
| 7 | C | 1.480645 | | -1.045972 | -0.000022 |
| 8 | C | -1.020795 | | -0.795004 | -0.00005 |
| 9 | C | 1.823959 | | 1.729098 | 0.000006 |
| 10 | C | 2.920563 | | 0.879225 | 0.000009 |
| 11 | C | -2.090444 | | 0.230202 | -0.000015 |
| 12 | C | 3.984163 | | -1.421036 | 0.000056 |
| 13 | C | -1.760918 | | 1.548759 | -0.000008 |
| 14 | C | -3.537474 | | -0.079551 | 0.000014 |
| 15 | H | 1.304745 | | -2.117221 | -0.000049 |
| 16 | H | 1.93532 | | 2.807923 | 0.000007 |
| 17 | H | 3.919501 | | 1.307288 | 0.000005 |
| 18 | H | 3.698085 | | -2.475362 | -0.000168 |
| 19 | H | 4.6104 | | -1.242342 | -0.88129 |
| 20 | H | 4.610064 | | -1.242646 | 0.881706 |
| 21 | H | -2.504283 | | 2.341212 | -0.000004 |
| 22 | H | -4.178238 | | 0.835392 | -0.000067 |

**Supplementary Table S3.** Optimized structure for 6-Ethyl-3-formylchromone (**3**) and cartesian Z-matrix.

| **6-Ethyl-3-formylchromone** (**3**) | | | 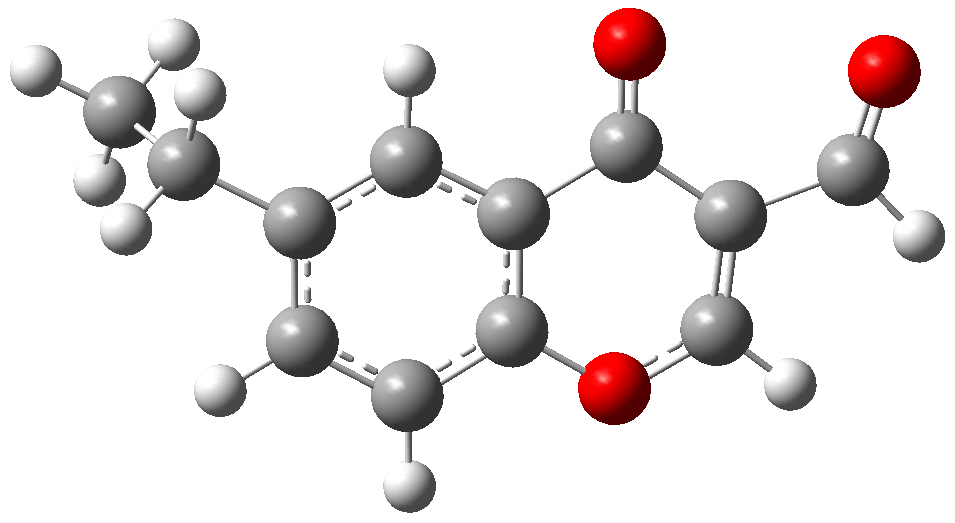 | | |
| --- | --- | --- | --- | --- | --- |
| Center  Number | Atom | Standard orientation: Coordinates (Angstroms) | | | |
|  |  | X | | Y | Z |
| 1 | O | 1.093826 | | 2.073066 | 0.133441 |
| 2 | O | 1.362957 | | -2.016312 | -0.193139 |
| 3 | O | 4.246438 | | -1.515298 | 0.078322 |
| 4 | C | -2.43604 | | -0.112783 | -0.329358 |
| 5 | C | -0.004112 | | -0.074122 | -0.13217 |
| 6 | C | -1.210757 | | -0.772751 | -0.287501 |
| 7 | C | -3.735819 | | -0.878011 | -0.458749 |
| 8 | C | -0.055775 | | 1.318488 | -0.018594 |
| 9 | C | -2.43988 | | 1.292291 | -0.212421 |
| 10 | C | -1.263908 | | 2.014077 | -0.057916 |
| 11 | C | 1.295263 | | -0.799725 | -0.093961 |
| 12 | C | 2.461937 | | 0.097904 | 0.073797 |
| 13 | C | -4.390426 | | -1.178185 | 0.901422 |
| 14 | C | 2.271433 | | 1.440057 | 0.172038 |
| 15 | C | 3.864632 | | -0.367749 | 0.144439 |
| 16 | H | -1.147461 | | -1.853046 | -0.378059 |
| 17 | H | -4.435841 | | -0.305014 | -1.078688 |
| 18 | H | -3.549885 | | -1.819403 | -0.987148 |
| 19 | H | -3.385801 | | 1.826223 | -0.249053 |
| 20 | H | -1.261819 | | 3.095268 | 0.027329 |
| 21 | H | -3.727927 | | -1.781414 | 1.529879 |
| 22 | H | -5.327474 | | -1.728043 | 0.768792 |
| 23 | H | -4.61408 | | -0.254371 | 1.444959 |
| 24 | H | 3.090602 | | 2.143187 | 0.295295 |
| 25 | H | 4.594638 | | 0.468025 | 0.271535 |

**Supplementary Table  S4.** Optimized structure for 6-Isopropyl-3-Formylchromone (**4**) and cartesian Z-matrix.

| **6-Isopropyl-3-Formylchromone (4)** | | | 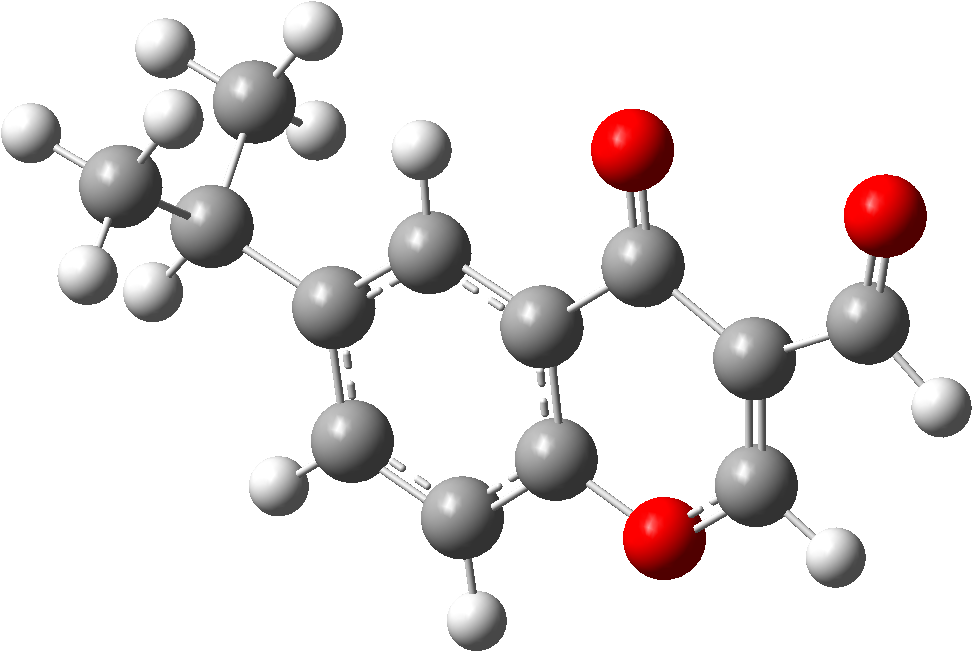 | | |
| --- | --- | --- | --- | --- | --- |
| Center  Number | Atom | Standard orientation: Coordinates (Angstroms) | | | |
|  |  | X | | Y | Z |
| 1 | O | 1.590579 | | 2.092416 | -0.000263 |
| 2 | O | 1.378789 | | -2.013624 | 0.000063 |
| 3 | O | 4.312217 | | -1.833442 | 0.000121 |
| 4 | C | -3.5797 | | -0.316688 | 0.000195 |
| 5 | C | -2.187422 | | 0.300914 | 0.000082 |
| 6 | C | -1.039424 | | -0.490639 | 0.000077 |
| 7 | C | 0.243474 | | 0.073541 | -0.000039 |
| 8 | C | -3.832842 | | -1.149874 | 1.270434 |
| 9 | C | -3.833016 | | -1.149904 | -1.26999 |
| 10 | C | -2.024762 | | 1.699837 | -0.000034 |
| 11 | C | 0.355833 | | 1.468268 | -0.000151 |
| 12 | C | -0.767369 | | 2.292209 | -0.000151 |
| 13 | C | 1.454024 | | -0.793369 | -0.000051 |
| 14 | C | 2.722416 | | -0.027978 | -0.00012 |
| 15 | C | 2.689922 | | 1.330829 | -0.000237 |
| 16 | C | 4.066171 | | -0.647518 | -0.000073 |
| 17 | H | -4.298733 | | 0.513056 | 0.000236 |
| 18 | H | -1.093138 | | -1.574837 | 0.000158 |
| 19 | H | -3.688525 | | -0.550977 | 2.174858 |
| 20 | H | -4.85716 | | -1.53694 | 1.277505 |
| 21 | H | -3.153034 | | -2.006726 | 1.324684 |
| 22 | H | -3.153202 | | -2.006747 | -1.324316 |
| 23 | H | -4.85733 | | -1.536983 | -1.276907 |
| 24 | H | -3.688835 | | -0.551025 | -2.174447 |
| 25 | H | -2.905814 | | 2.335913 | -0.000031 |
| 26 | H | -0.642052 | | 3.36951 | -0.000238 |
| 27 | H | 3.589341 | | 1.940489 | -0.000316 |
| 28 | H | 4.891972 | | 0.104691 | -0.000214 |

**Supplementary Table  S5.** Optimized structure for 6-hydroxy-3-formylchromone (**5**) and cartesian Z-matrix.

| **6-hydroxy-3-formylchromone (5)** | | | **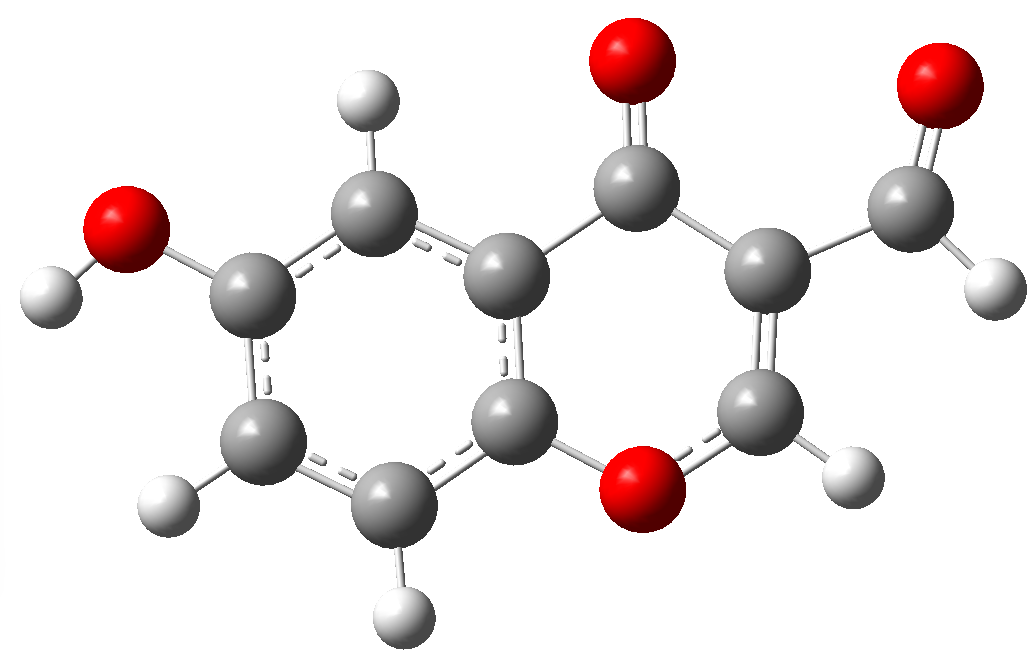** | | |
| --- | --- | --- | --- | --- | --- |
| Center  Number | Atom | Standard orientation: Coordinates (Angstroms) | | | |
|  |  | X | | Y | Z |
| 1 | O | -0.459815 | | 2.04509 | 0.000187 |
| 2 | O | 3.795126 | | -1.475112 | 0.000003 |
| 3 | O | -1.239494 | | -1.992413 | -0.000341 |
| 4 | O | -4.042381 | | -1.113433 | 0.000065 |
| 5 | C | 0.36587 | | -0.240775 | -0.000073 |
| 6 | C | 0.589221 | | 1.141889 | 0.000127 |
| 7 | C | 1.46637 | | -1.103302 | -0.000109 |
| 8 | C | -1.02072 | | -0.790283 | -0.000256 |
| 9 | C | 2.759874 | | -0.58854 | 0.000049 |
| 10 | C | 1.877317 | | 1.672768 | 0.000286 |
| 11 | C | -2.065783 | | 0.257878 | -0.000149 |
| 12 | C | 2.96096 | | 0.804281 | 0.000246 |
| 13 | C | -1.708433 | | 1.570139 | 0.000058 |
| 14 | C | -3.518737 | | -0.021282 | -0.000272 |
| 15 | H | 1.29221 | | -2.173231 | -0.000265 |
| 16 | H | 2.01503 | | 2.748355 | 0.000441 |
| 17 | H | 3.971224 | | 1.206536 | 0.00037 |
| 18 | H | -2.435891 | | 2.377214 | 0.000136 |
| 19 | H | -4.139816 | | 0.907173 | 0.00043 |
| 20 | H | 4.634121 | | -0.995737 | 0.000125 |

**Supplementary Table S6.** Optimized structure for 3-Formyl-6-methoxychromone (**6**) and cartesian Z-matrix.

| **3-Formyl-6-methoxychromone (6)** | | | 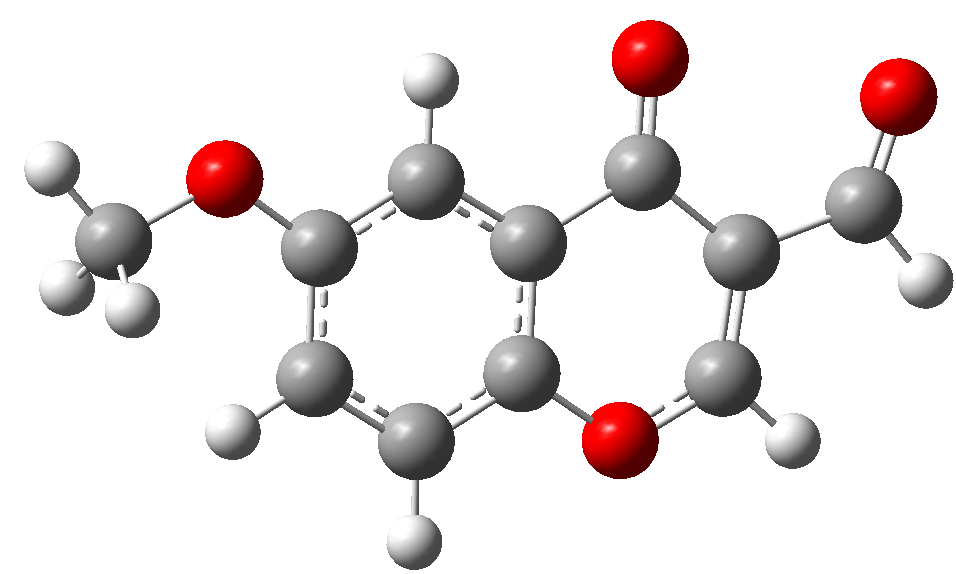 | | |
| --- | --- | --- | --- | --- | --- |
| Center  Number | Atom | Standard orientation: Coordinates (Angstroms) | | | |
|  |  | X | | Y | Z |
| 1 | O | -0.998161 | | 2.062848 | 0.000249 |
| 2 | O | 3.457263 | | -1.20202 | -0.000081 |
| 3 | O | -1.508695 | | -2.01855 | -0.000208 |
| 4 | O | -4.364346 | | -1.325893 | -0.000377 |
| 5 | C | -0.022729 | | -0.163947 | 0.000003 |
| 6 | C | 0.109223 | | 1.231492 | 0.000164 |
| 7 | C | 1.132862 | | -0.94705 | -0.000074 |
| 8 | C | -1.369508 | | -0.804558 | -0.000084 |
| 9 | C | 2.394957 | | -0.35113 | 0.000007 |
| 10 | C | 1.358567 | | 1.843213 | 0.000247 |
| 11 | C | -2.481859 | | 0.172316 | -0.000015 |
| 12 | C | 2.503877 | | 1.052947 | 0.000168 |
| 13 | C | -2.211996 | | 1.505507 | 0.000156 |
| 14 | C | -3.913026 | | -0.20181 | -0.000133 |
| 15 | C | 4.768273 | | -0.65505 | -0.000004 |
| 16 | H | 1.032979 | | -2.026503 | -0.000197 |
| 17 | H | 1.424277 | | 2.925766 | 0.000372 |
| 18 | H | 3.473689 | | 1.534784 | 0.000232 |
| 19 | H | -2.991816 | | 2.26219 | 0.000232 |
| 20 | H | -4.593752 | | 0.683983 | 0.000048 |
| 21 | H | 4.953491 | | -0.047256 | -0.89468 |
| 22 | H | 5.447337 | | -1.50817 | -0.000094 |
| 23 | H | 4.95347 | | -0.047461 | 0.894816 |

**Supplementary Table S7.** Optimized structure for 6-Ethoxy-3-Formylchromone (**7**) and cartesian Z-matrix.

| **6-Ethoxy-3-Formylchromone (7)** | | | 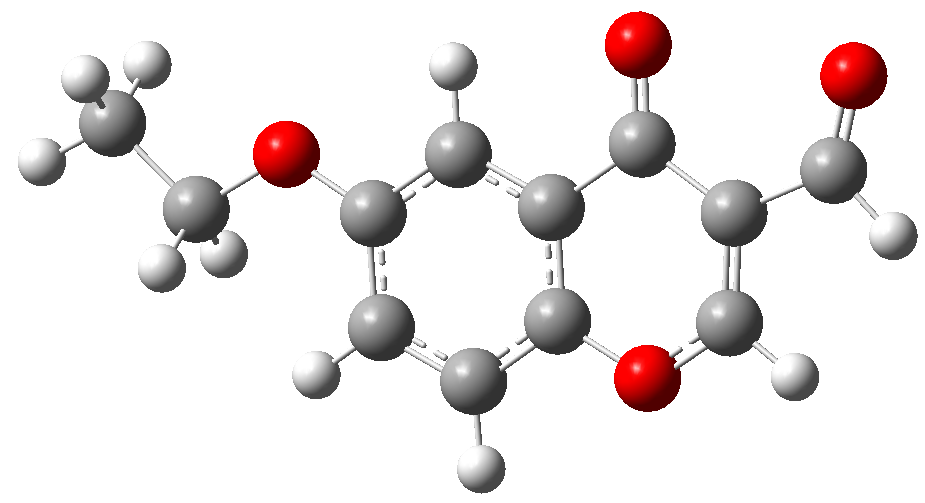 | | |
| --- | --- | --- | --- | --- | --- |
| Center  Number | Atom | Standard orientation: Coordinates (Angstroms) | | | |
|  |  | X | | Y | Z |
| 1 | O | 1.625409 | | 2.049664 | -0.000199 |
| 2 | O | -3.135951 | | -0.751319 | 0.000079 |
| 3 | O | 1.723141 | | -2.062512 | 0.000179 |
| 4 | O | 4.634137 | | -1.659977 | 0.000108 |
| 5 | C | 0.430941 | | -0.067916 | 0.000122 |
| 6 | C | 0.439868 | | 1.333628 | -0.000092 |
| 7 | C | -0.797655 | | -0.730682 | 0.000165 |
| 8 | C | 1.70639 | | -0.840609 | 0.000351 |
| 9 | C | -1.994506 | | -0.011966 | 0.000014 |
| 10 | C | -0.741808 | | 2.06752 | -0.000241 |
| 11 | C | 2.911338 | | 0.019507 | 0.000041 |
| 12 | C | -1.960801 | | 1.396529 | -0.000188 |
| 13 | C | 2.77688 | | 1.373187 | -0.000149 |
| 14 | C | 4.29756 | | -0.496299 | -0.000051 |
| 15 | C | -4.399995 | | -0.083218 | -0.000066 |
| 16 | H | -0.806397 | | -1.814697 | 0.000337 |
| 17 | H | -0.698563 | | 3.151219 | -0.000408 |
| 18 | H | -2.87696 | | 1.973592 | -0.000309 |
| 19 | H | 3.628908 | | 2.04755 | -0.000325 |
| 20 | H | 5.063703 | | 0.316788 | -0.000255 |
| 21 | H | -4.482767 | | 0.558109 | 0.888684 |
| 22 | H | -4.4827 | | 0.557852 | -0.889007 |
| 23 | C | -5.478868 | | -1.150566 | 0.000049 |
| 24 | H | -6.468123 | | -0.683308 | -0.000056 |
| 25 | H | -5.393491 | | -1.784448 | -0.886467 |
| 26 | H | -5.393558 | | -1.784191 | 0.886754 |

**Supplementary Table S8.** Optimized structure for 6-Amino-3-Formylchromone (**8**) and cartesian Z-matrix.

| **6-Amino-3-Formylchromone (8)** | | | 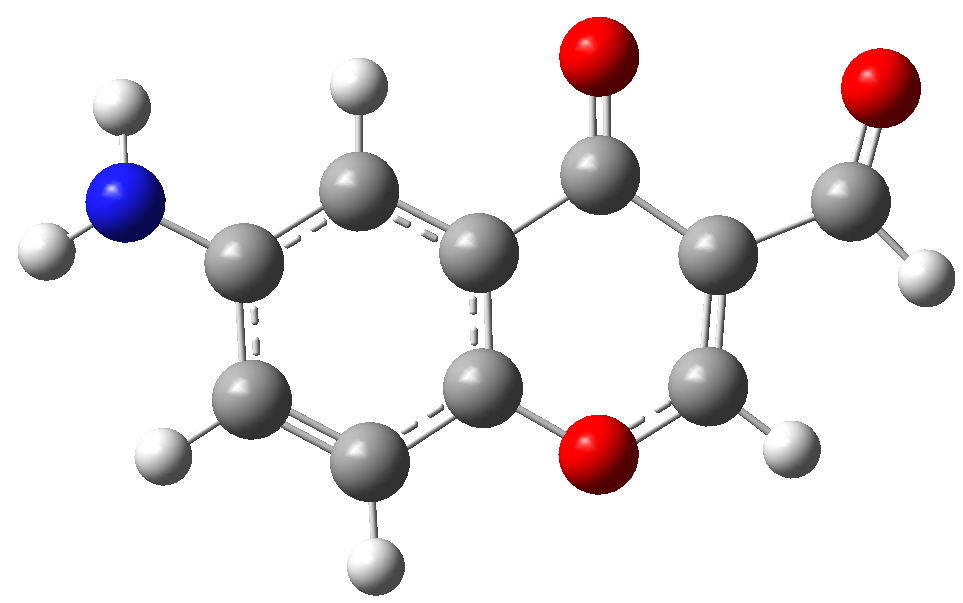 | | |
| --- | --- | --- | --- | --- | --- |
| Center  Number | Atom | Standard orientation: Coordinates (Angstroms) | | | |
|  |  | X | | Y | Z |
| 1 | O | 0.490182 | | 2.051161 | 0.003163 |
| 2 | O | 1.221772 | | -1.995751 | 0.002446 |
| 3 | O | 4.036141 | | -1.147361 | 0.002214 |
| 4 | N | -3.878335 | | -1.402945 | -0.067542 |
| 5 | C | -0.361843 | | -0.22492 | -0.005898 |
| 6 | C | -0.568757 | | 1.158264 | -0.001129 |
| 7 | C | -1.472137 | | -1.075076 | -0.007664 |
| 8 | C | 1.016788 | | -0.789822 | -0.000913 |
| 9 | C | -2.772905 | | -0.562508 | -0.006358 |
| 10 | C | -1.854998 | | 1.697593 | 0.002395 |
| 11 | C | 2.073936 | | 0.245328 | 0.000661 |
| 12 | C | -2.945005 | | 0.841828 | -0.00128 |
| 13 | C | 1.731924 | | 1.563031 | 0.002294 |
| 14 | C | 3.522566 | | -0.049905 | 0.001288 |
| 15 | H | -1.281805 | | -2.143996 | -0.011768 |
| 16 | H | -1.984103 | | 2.774473 | 0.00785 |
| 17 | H | -3.94953 | | 1.255889 | -0.005835 |
| 18 | H | 2.469461 | | 2.360976 | 0.003668 |
| 19 | H | -4.742735 | | -1.027713 | 0.295821 |
| 20 | H | -3.71887 | | -2.358312 | 0.219002 |
| 21 | H | 4.153753 | | 0.872024 | 0.0011 |

**Supplementary Table S9.** Optimized structure for 6-Fluoro-3-formylchromone (**9**) and cartesian Z-matrix.

| **6-Fluoro-3-formylchromone (9)** | | | 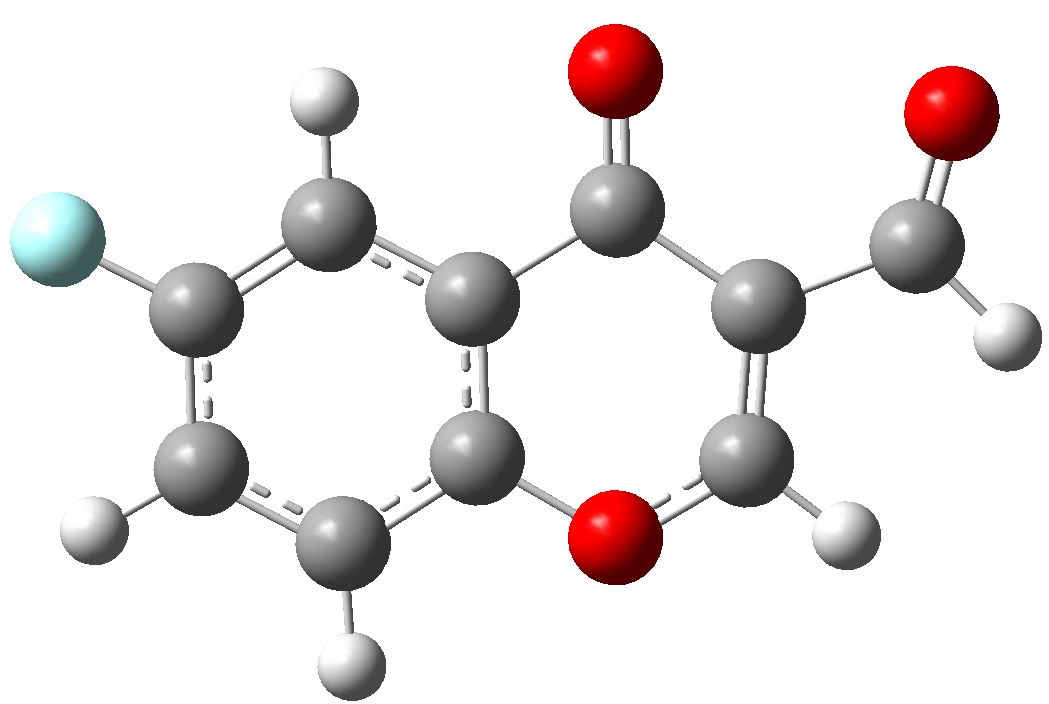 | | |
| --- | --- | --- | --- | --- | --- |
| Center  Number | Atom | Standard orientation: Coordinates (Angstroms) | | | |
|  |  | X | | Y | Z |
| 1 | F | -3.805255 | | -1.417001 | 0.000109 |
| 2 | O | 0.435615 | | 2.04086 | -0.000091 |
| 3 | O | 1.24256 | | -1.989355 | 0.000039 |
| 4 | O | 4.039494 | | -1.094404 | 0.00022 |
| 5 | C | -0.369547 | | -0.246778 | -0.000153 |
| 6 | C | -0.606042 | | 1.132596 | -0.000096 |
| 7 | C | 1.019419 | | -0.788325 | -0.000379 |
| 8 | C | -1.467662 | | -1.118722 | -0.000071 |
| 9 | C | 2.057205 | | 0.266689 | -0.000059 |
| 10 | C | -1.898186 | | 1.661753 | 0.000007 |
| 11 | C | -2.743966 | | -0.589215 | 0.000042 |
| 12 | C | -2.978528 | | 0.790165 | 0.000079 |
| 13 | C | 1.689863 | | 1.575132 | -0.000028 |
| 14 | C | 3.513094 | | -0.004102 | 0.000194 |
| 15 | H | -1.292205 | | -2.188336 | -0.000127 |
| 16 | H | -2.036244 | | 2.737151 | 0.00005 |
| 17 | H | -3.998878 | | 1.1566 | 0.000168 |
| 18 | H | 2.409803 | | 2.388718 | 0.000106 |
| 19 | H | 4.129568 | | 0.926906 | 0.000253 |

**Supplementary Table S10.** Optimized structure for 6-Chloro-3-formylchromone (**10**) and cartesian Z-matrix.

| **6-Chloro-3-formylchromone (10)** | | | 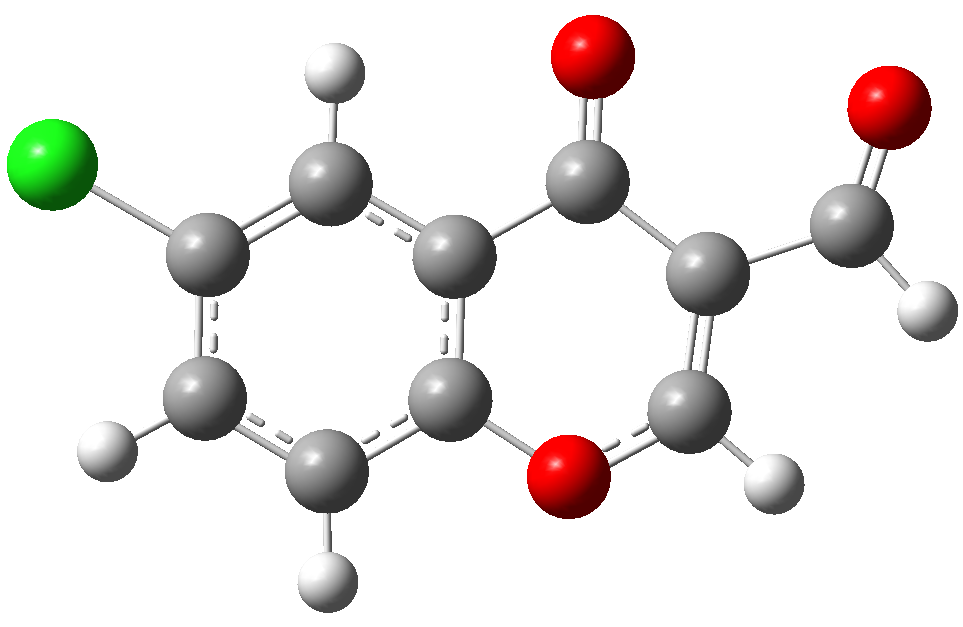 | | |
| --- | --- | --- | --- | --- | --- |
| Center  Number | Atom | Standard orientation: Coordinates (Angstroms) | | | |
|  |  | X | | Y | Z |
| 1 | Cl | 3.947685 | | -1.069461 | -0.00079 |
| 2 | O | -1.02972 | | 2.082733 | 0.000369 |
| 3 | O | -1.352027 | | -2.014607 | -0.000002 |
| 4 | O | -4.235192 | | -1.459876 | 0.000321 |
| 5 | C | 0.040585 | | -0.091187 | 0.00017 |
| 6 | C | 0.111028 | | 1.305541 | 0.000162 |
| 7 | C | 2.507105 | | 1.244722 | -0.000406 |
| 8 | C | 1.33147 | | 1.982559 | -0.000115 |
| 9 | C | 1.235303 | | -0.825777 | -0.00014 |
| 10 | C | -1.273464 | | -0.795955 | 0.000586 |
| 11 | C | 2.449261 | | -0.157604 | -0.000422 |
| 12 | C | -2.429962 | | 0.128355 | 0.000508 |
| 13 | C | -2.221016 | | 1.47064 | 0.000496 |
| 14 | C | -3.843661 | | -0.314428 | 0.000503 |
| 15 | H | 1.343432 | | 3.066721 | -0.000117 |
| 16 | H | 1.171585 | | -1.907821 | -0.000121 |
| 17 | H | -3.031767 | | 2.193638 | 0.000525 |
| 18 | H | -4.56691 | | 0.536038 | 0.000232 |
| 19 | H | 3.468641 | | 1.745074 | -0.000632 |

**Supplementary Table S11.** Optimized structure for 6-Bromo-3-formylchromone (**11**) and cartesian Z-matrix.

| **6-Bromo-3-formylchromone (11)** | | | 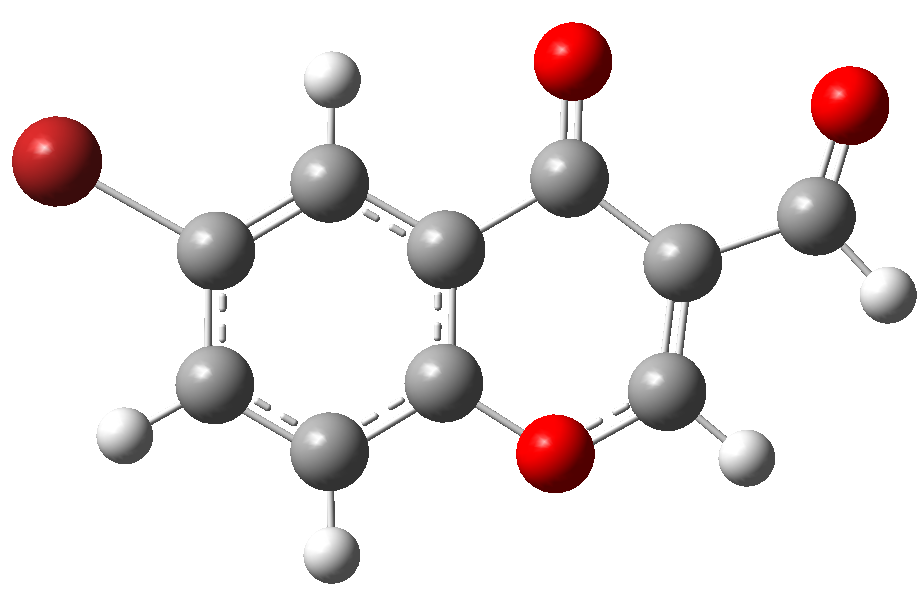 | | |
| --- | --- | --- | --- | --- | --- |
| Center  Number | Atom | Standard orientation: Coordinates (Angstroms) | | | |
|  |  | X | | Y | Z |
| 1 | Br | -3.547729 | | -0.626457 | 0.000537 |
| 2 | O | 1.853057 | | 2.088648 | -0.000391 |
| 3 | O | 1.773665 | | -2.020727 | 0.000062 |
| 4 | O | 4.697098 | | -1.75018 | -0.000746 |
| 5 | C | 0.575614 | | 0.029793 | -0.000032 |
| 6 | C | 0.641912 | | 1.426797 | -0.000174 |
| 7 | C | 1.814255 | | -0.800245 | -0.000096 |
| 8 | C | -0.686141 | | -0.58335 | 0.000184 |
| 9 | C | 3.055603 | | 0.006749 | -0.000428 |
| 10 | C | -0.50646 | | 2.220118 | -0.000103 |
| 11 | C | -1.828161 | | 0.200052 | 0.000251 |
| 12 | C | -1.748657 | | 1.60058 | 0.00011 |
| 13 | C | 2.978872 | | 1.362978 | -0.000524 |
| 14 | C | 4.419309 | | -0.57198 | -0.000675 |
| 15 | H | -0.727812 | | -1.666302 | 0.000299 |
| 16 | H | -0.412307 | | 3.300362 | -0.000216 |
| 17 | H | -2.655403 | | 2.194043 | 0.000165 |
| 18 | H | 3.85646 | | 2.003199 | -0.000726 |
| 19 | H | 5.222161 | | 0.203825 | -0.000798 |

**Supplementary Table S12.** Optimized structure 6-Cyano-3-formylchromone (**12**) and cartesian Z-matrix.

| **6-Cyano-3-formylchromone**  **(12)** | | | 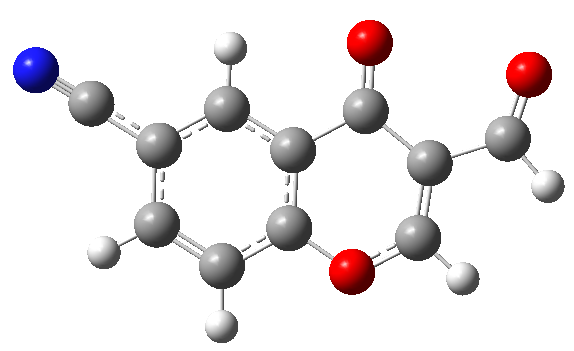 | | |
| --- | --- | --- | --- | --- | --- |
| Center  Number | Atom | Standard orientation: Coordinates (Angstroms) | | | |
|  |  | X | | Y | Z |
| 1 | O | 0.894274 | | 2.0632 | 0.000029 |
| 2 | O | 1.246759 | | -2.029489 | -0.000054 |
| 3 | O | 4.124706 | | -1.458412 | -0.000166 |
| 4 | C | -0.157805 | | -0.11567 | 0 |
| 5 | C | -0.238654 | | 1.281534 | 0.00004 |
| 6 | C | 1.161959 | | -0.812079 | -0.000057 |
| 7 | C | -1.347602 | | -0.8526 | 0.000017 |
| 8 | C | 2.312475 | | 0.120132 | -0.000071 |
| 9 | C | -1.46262 | | 1.955602 | 0.000095 |
| 10 | C | 2.093424 | | 1.459478 | -0.00003 |
| 11 | C | -2.580893 | | -0.198699 | 0.00007 |
| 12 | C | -2.631854 | | 1.213689 | 0.00011 |
| 13 | C | 3.730301 | | -0.314532 | -0.000132 |
| 14 | H | -1.277057 | | -1.934731 | -0.000012 |
| 15 | H | -1.47563 | | 3.039576 | 0.000126 |
| 16 | H | 2.896789 | | 2.190384 | -0.000035 |
| 17 | H | -3.59425 | | 1.713166 | 0.000152 |
| 18 | H | 4.449161 | | 0.539011 | -0.000119 |
| 19 | C | -3.801814 | | -0.95169 | 0.000084 |
| 20 | N | -4.798061 | | -1.551542 | 0.000095 |

**Supplementary Table S13.** Optimized structure for 6-Nitro-3-formylchromone (**13**) and cartesian Z-matrix.

| **6-Nitro-3-formylchromone (13)** | | | 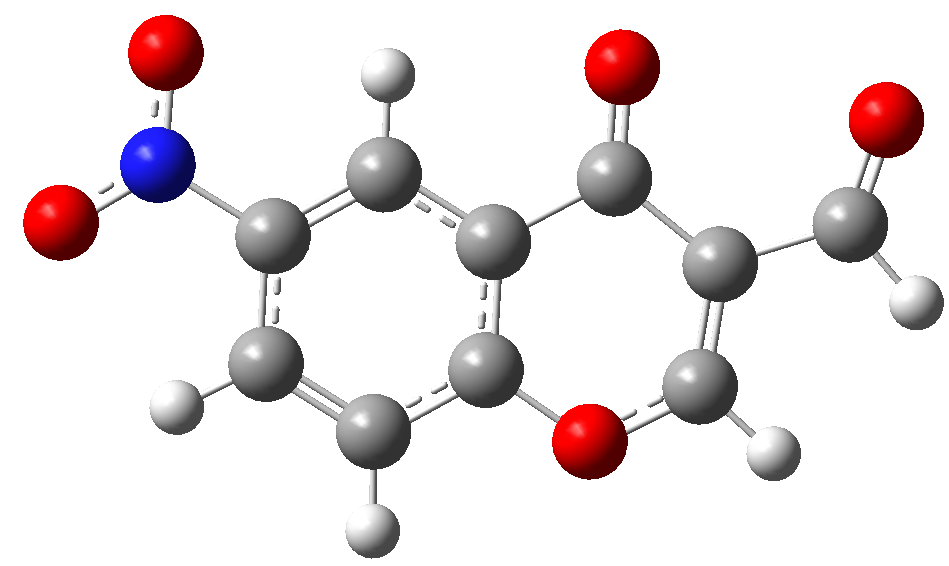 | | |
| --- | --- | --- | --- | --- | --- |
| Center  Number | Atom | Standard orientation: Coordinates (Angstroms) | | | |
|  |  | X | | Y | Z |
| 1 | O | -1.324616 | | 2.100286 | 0.000089 |
| 2 | O | -1.516922 | | -2.002568 | 0.000033 |
| 3 | O | 4.524788 | | -0.051728 | -0.000073 |
| 4 | O | 3.456254 | | -1.954146 | -0.000113 |
| 5 | O | -4.414106 | | -1.546064 | 0.000071 |
| 6 | N | 3.49568 | | -0.72667 | -0.000085 |
| 7 | C | -0.188395 | | -0.035103 | -0.000089 |
| 8 | C | -0.1627 | | 1.365633 | 0.000007 |
| 9 | C | 1.03035 | | -0.724508 | -0.000107 |
| 10 | C | -1.479673 | | -0.783379 | -0.000218 |
| 11 | C | 2.211788 | | -0.002432 | -0.000052 |
| 12 | C | 1.03306 | | 2.090977 | 0.000062 |
| 13 | C | -2.666424 | | 0.103427 | 0.000037 |
| 14 | C | 2.232799 | | 1.398755 | 0.000029 |
| 15 | C | -2.500504 | | 1.449634 | 0.000126 |
| 16 | C | -4.066453 | | -0.387359 | 0.000174 |
| 17 | H | 1.028268 | | -1.807648 | -0.000185 |
| 18 | H | 1.000488 | | 3.174448 | 0.000139 |
| 19 | H | 3.184901 | | 1.913632 | 0.00007 |
| 20 | H | -3.331025 | | 2.149392 | 0.000276 |
| 21 | H | -4.818663 | | 0.436756 | 0.000427 |

**Supplementary Table S14.** Optimized structure for 7-Bromo-3-formylchromone (**14**) and cartesian Z-matrix.

| **7-Bromo-3-formylchromone (14)** | | | 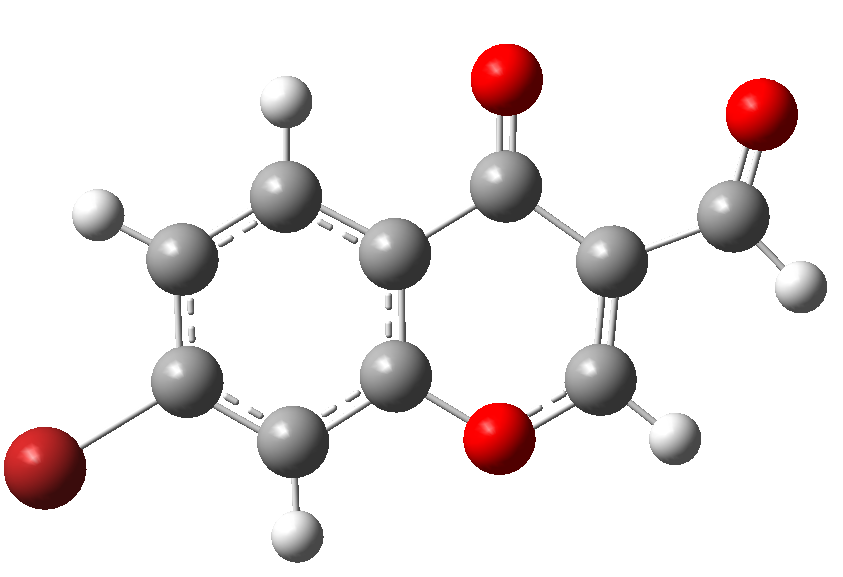 | | |
| --- | --- | --- | --- | --- | --- |
| Center  Number | Atom | Standard orientation: Coordinates (Angstroms) | | | |
|  |  | X | | Y | Z |
| 1 | Br | 3.803965 | | -0.123959 | 0.000437 |
| 2 | O | -1.107537 | | -1.836646 | -0.000336 |
| 3 | O | -2.801851 | | 1.908127 | -0.000099 |
| 4 | O | -5.326032 | | 0.407435 | -0.000332 |
| 5 | C | -0.840532 | | 0.571509 | -0.000123 |
| 6 | C | -0.299199 | | -0.718427 | -0.00016 |
| 7 | C | -2.312722 | | 0.788524 | -0.000339 |
| 8 | C | 0.046605 | | 1.659003 | 0.000091 |
| 9 | C | -3.088877 | | -0.473538 | -0.000421 |
| 10 | C | 1.076507 | | -0.95191 | -0.000001 |
| 11 | C | 1.418751 | | 1.461635 | 0.000258 |
| 12 | C | 1.918821 | | 0.151402 | 0.000209 |
| 13 | C | -2.437146 | | -1.664605 | -0.000445 |
| 14 | C | -4.568949 | | -0.537166 | -0.000503 |
| 15 | H | -0.382027 | | 2.655684 | 0.000113 |
| 16 | H | 1.457868 | | -1.965359 | -0.000033 |
| 17 | H | 2.103492 | | 2.301265 | 0.000419 |
| 18 | H | -2.953699 | | -2.62022 | -0.000538 |
| 19 | H | -4.960617 | | -1.582682 | -0.000511 |

**Supplementary Table S15.** Optimized structure for 6,8-dichloro-3-formylchromone (**15**) and cartesian Z-matrix.

| **6,8-dichloro-3-formylchromone (15)** | | | 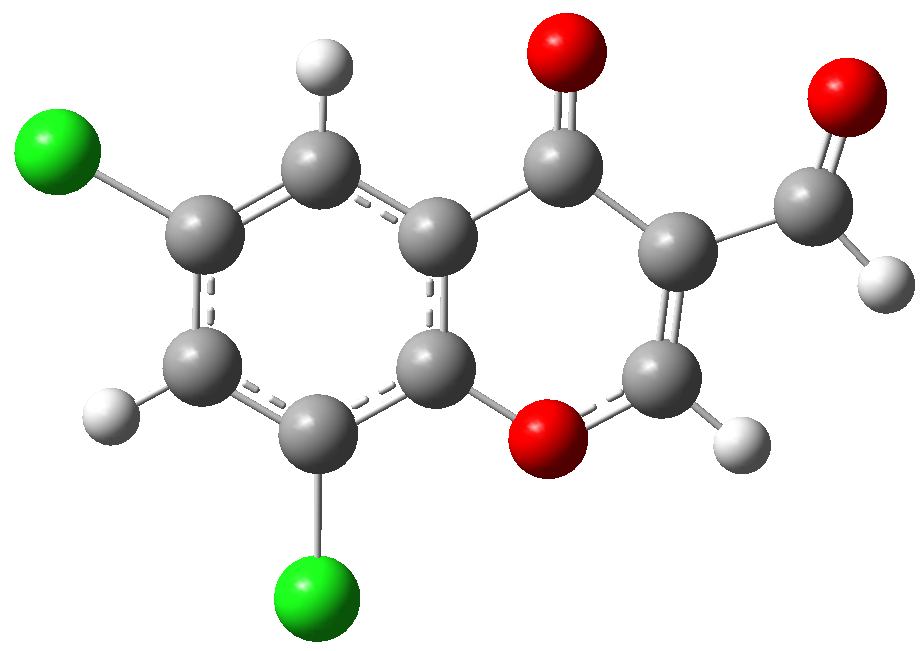 | | |
| --- | --- | --- | --- | --- | --- |
| Center  Number | Atom | Standard orientation: Coordinates (Angstroms) | | | |
|  |  | X | | Y | Z |
| 1 | Cl | 3.404141 | | -2.23525 | -0.000302 |
| 2 | O | -0.902131 | | 1.789089 | -0.000026 |
| 3 | O | -1.965333 | | -2.174486 | 0.000282 |
| 4 | O | -4.698537 | | -1.110206 | 0.000877 |
| 5 | C | -0.244322 | | -0.540036 | -0.000304 |
| 6 | C | 0.072251 | | 0.822798 | -0.000183 |
| 7 | C | 2.428043 | | 0.301103 | -0.000168 |
| 8 | C | 1.409995 | | 1.245594 | -0.000131 |
| 9 | C | 0.785415 | | -1.489612 | -0.000319 |
| 10 | C | -1.66943 | | -0.990478 | -0.000555 |
| 11 | C | 2.103361 | | -1.062501 | -0.000251 |
| 12 | C | -2.638054 | | 0.127117 | 0.000122 |
| 13 | C | -2.187225 | | 1.407491 | 0.000173 |
| 14 | C | -4.109572 | | -0.053038 | 0.000676 |
| 15 | H | 0.516079 | | -2.539191 | -0.000432 |
| 16 | H | -2.847654 | | 2.269559 | 0.000501 |
| 17 | H | -4.66782 | | 0.913166 | 0.001195 |
| 18 | Cl | 1.793644 | | 2.946349 | 0.000033 |
| 19 | H | 3.462302 | | 0.62197 | -0.000118 |

**Supplementary Table S16.** Optimized structure for 6,7-dichloro-3-formylchromone (**16**) and cartesian Z-matrix.

| **6,7-dichloro-3-formylchromone (16)** | | | 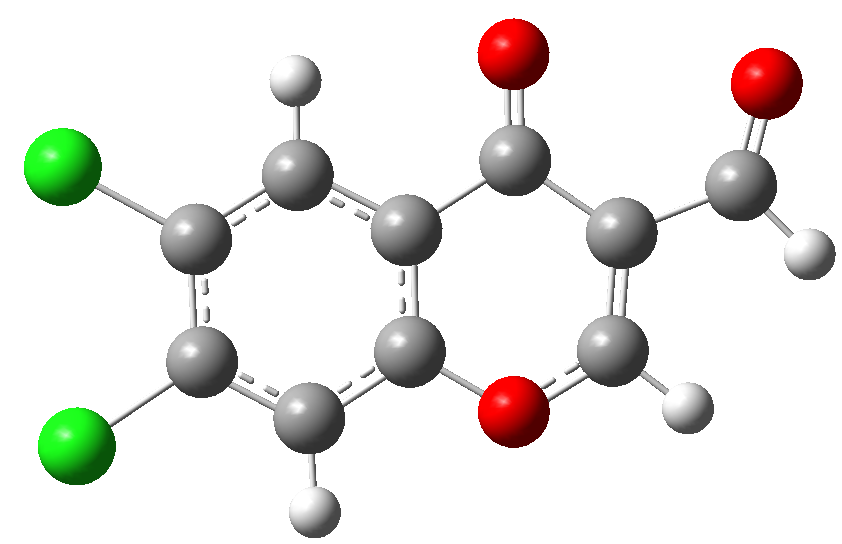 | | |
| --- | --- | --- | --- | --- | --- |
| Center  Number | Atom | Standard orientation: Coordinates (Angstroms) | | | |
|  |  | X | | Y | Z |
| 1 | Cl | -3.130891 | | 1.911815 | -0.000267 |
| 2 | Cl | -3.738386 | | -1.248 | -0.000112 |
| 3 | O | 1.247083 | | -2.036988 | 0.000177 |
| 4 | O | 2.221852 | | 1.957401 | -0.000024 |
| 5 | O | 4.978049 | | 0.948987 | 0.000218 |
| 6 | C | 0.542519 | | 0.279885 | 0.00001 |
| 7 | C | 0.249064 | | -1.086577 | 0.000075 |
| 8 | C | 1.950639 | | 0.767011 | 0.000049 |
| 9 | C | -0.523285 | | 1.187569 | -0.000097 |
| 10 | C | -1.060854 | | -1.560218 | 0.000039 |
| 11 | C | 2.943837 | | -0.331554 | 0.000162 |
| 12 | C | -1.837463 | | 0.743119 | -0.000133 |
| 13 | C | -2.106176 | | -0.643234 | -0.000064 |
| 14 | C | 2.522818 | | -1.622502 | 0.000217 |
| 15 | C | 4.4109 | | -0.120119 | 0.000223 |
| 16 | H | -0.290766 | | 2.246509 | -0.00015 |
| 17 | H | -1.255177 | | -2.625654 | 0.000091 |
| 18 | H | 3.206154 | | -2.466744 | 0.000301 |
| 19 | H | 4.989644 | | -1.074456 | 0.000345 |

**Supplementary Table S17.** Optimized structure for Myricetin (**17**) and cartesian Z-matrix.

| **Myricetin (17)** | | | 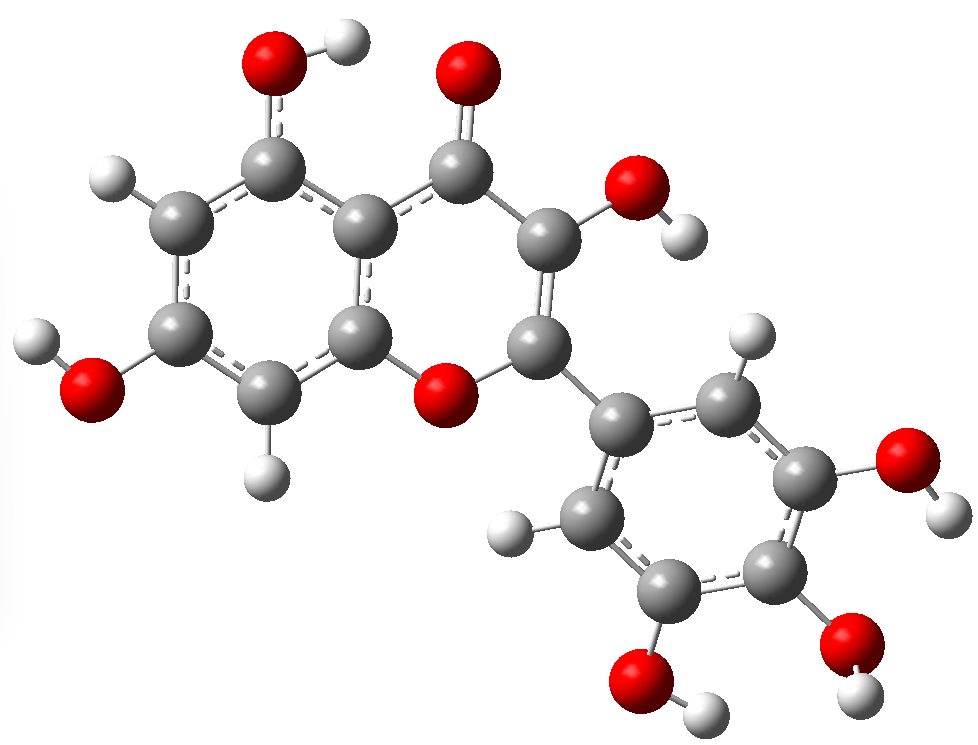 | | |
| --- | --- | --- | --- | --- | --- |
| Center  Number | Atom | Standard orientation: Coordinates (Angstroms) | | | |
|  |  | X | | Y | Z |
| 1 | O | 0.565043 | | -0.7672 | -0.139044 |
| 2 | O | -0.298822 | | 2.733007 | 0.498232 |
| 3 | O | 4.688201 | | 1.658209 | 0.210537 |
| 4 | O | 2.398239 | | 2.841966 | 0.428194 |
| 5 | O | 4.645751 | | -3.060268 | -0.502598 |
| 6 | O | -3.934535 | | -2.562958 | 1.0467 |
| 7 | O | -4.772698 | | 1.627687 | -1.092902 |
| 8 | O | -5.732818 | | -0.752731 | -0.041782 |
| 9 | C | 2.590414 | | 0.511032 | 0.044875 |
| 10 | C | -0.187123 | | 0.367876 | 0.027887 |
| 11 | C | 1.927801 | | -0.71655 | -0.132996 |
| 12 | C | -1.63421 | | 0.083413 | -0.001479 |
| 13 | C | 0.389068 | | 1.586649 | 0.252351 |
| 14 | C | 1.847609 | | 1.735081 | 0.252212 |
| 15 | C | 4.016813 | | 0.515161 | 0.03912 |
| 16 | C | 2.606223 | | -1.915114 | -0.317373 |
| 17 | C | -2.533119 | | 1.017866 | -0.550529 |
| 18 | C | -2.103678 | | -1.126347 | 0.534131 |
| 19 | C | 4.003628 | | -1.875692 | -0.320163 |
| 20 | C | 4.711747 | | -0.677768 | -0.144736 |
| 21 | C | -3.470118 | | -1.401615 | 0.518146 |
| 22 | C | -3.900469 | | 0.733274 | -0.560975 |
| 23 | C | -4.360418 | | -0.481257 | -0.046542 |
| 24 | H | 2.074459 | | -2.847175 | -0.456754 |
| 25 | H | -2.191174 | | 1.92736 | -1.030855 |
| 26 | H | -1.422226 | | -1.8471 | 0.967277 |
| 27 | H | 5.797293 | | -0.657856 | -0.147516 |
| 28 | H | -1.223435 | | 2.515073 | 0.689429 |
| 29 | H | 3.998448 | | 2.371409 | 0.3299 |
| 30 | H | 5.600434 | | -2.910127 | -0.487291 |
| 31 | H | -4.902795 | | -2.500389 | 1.06861 |
| 32 | H | -5.666948 | | 1.30331 | -0.900274 |
| 33 | H | -5.955956 | | -1.232261 | -0.854808 |

**Supplementary Table S18.** Optimized structure for Vitexin (**18**) and cartesian Z-matrix.

| **Vitexin (18)** | | | 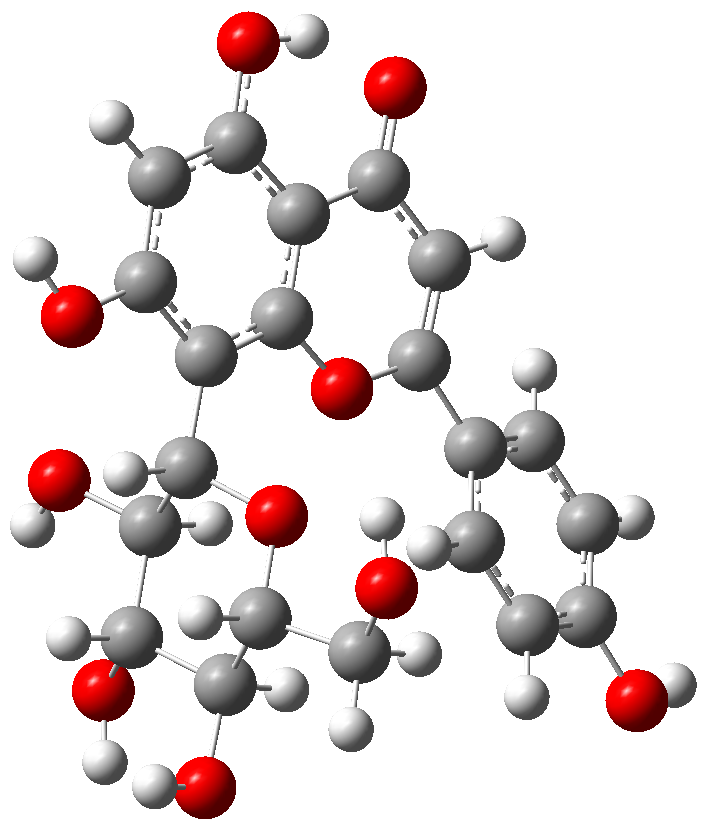 | | |
| --- | --- | --- | --- | --- | --- |
| Center  Number | Atom | Standard orientation: Coordinates (Angstroms) | | | |
|  |  | X | | Y | Z |
| 1 | O | -0.042847 | | -1.730131 | -0.977243 |
| 2 | O | 1.015898 | | -2.003735 | 2.510458 |
| 3 | O | -1.672172 | | -2.911813 | 2.619615 |
| 4 | O | -2.725548 | | -4.015378 | 0.168269 |
| 5 | O | 0.351794 | | 0.958776 | 0.053026 |
| 6 | O | -0.877544 | | -2.56247 | -3.489225 |
| 7 | O | 3.608121 | | -2.459198 | -0.06818 |
| 8 | O | 5.00003 | | 2.087191 | -0.212543 |
| 9 | O | 3.173672 | | 3.895555 | -0.160414 |
| 10 | O | -5.639782 | | 2.774965 | 0.025015 |
| 11 | C | 0.099575 | | -1.839028 | 1.441954 |
| 12 | C | -0.916045 | | -2.980308 | 1.419658 |
| 13 | C | 0.88976 | | -1.849704 | 0.118095 |
| 14 | C | -1.810766 | | -2.916085 | 0.182766 |
| 15 | C | -0.94564 | | -2.835206 | -1.081004 |
| 16 | C | 1.947414 | | -0.780209 | 0.005834 |
| 17 | C | -1.734313 | | -2.615208 | -2.366509 |
| 18 | C | 1.671056 | | 0.591757 | -0.010357 |
| 19 | C | 3.311035 | | -1.130691 | -0.066073 |
| 20 | C | 2.664032 | | 1.582297 | -0.081734 |
| 21 | C | 4.334709 | | -0.179955 | -0.138629 |
| 22 | C | 4.02593 | | 1.17742 | -0.144006 |
| 23 | C | 2.306038 | | 2.992448 | -0.098994 |
| 24 | C | -0.025299 | | 2.267682 | 0.016011 |
| 25 | C | 0.888316 | | 3.272068 | -0.061854 |
| 26 | C | -1.485855 | | 2.418005 | 0.04295 |
| 27 | C | -2.085738 | | 3.61612 | 0.461429 |
| 28 | C | -2.317321 | | 1.357718 | -0.365416 |
| 29 | C | -3.467984 | | 3.758912 | 0.462894 |
| 30 | C | -3.698032 | | 1.493544 | -0.36825 |
| 31 | C | -4.281733 | | 2.697669 | 0.045721 |
| 32 | H | -0.447343 | | -0.890551 | 1.530507 |
| 33 | H | -0.346092 | | -3.928606 | 1.383251 |
| 34 | H | 1.395208 | | -2.820031 | 0.046141 |
| 35 | H | -2.440833 | | -2.022163 | 0.249601 |
| 36 | H | -0.362885 | | -3.769407 | -1.184059 |
| 37 | H | -2.331951 | | -1.693882 | -2.263744 |
| 38 | H | -2.429066 | | -3.446033 | -2.515932 |
| 39 | H | 0.479992 | | -2.145053 | 3.304182 |
| 40 | H | -2.414903 | | -3.524196 | 2.511757 |
| 41 | H | -2.210389 | | -4.825507 | 0.037543 |
| 42 | H | 5.376353 | | -0.482117 | -0.190398 |
| 43 | H | -0.184703 | | -1.925556 | -3.259605 |
| 44 | H | 0.57064 | | 4.304068 | -0.130367 |
| 45 | H | 4.567409 | | -2.568811 | -0.101645 |
| 46 | H | 4.542167 | | 2.979651 | -0.206221 |
| 47 | H | -1.47076 | | 4.438442 | 0.81151 |
| 48 | H | -1.861878 | | 0.430063 | -0.69348 |
| 49 | H | -3.917487 | | 4.690344 | 0.799089 |
| 50 | H | -4.342975 | | 0.683668 | -0.69169 |
| 51 | H | -5.918287 | | 3.650107 | 0.326425 |

**Supplementary Table S19.** Optimized structure for Dapagliflozin (ref. drug) and cartesian Z-matrix.

| **Vitexin (18)** | | | 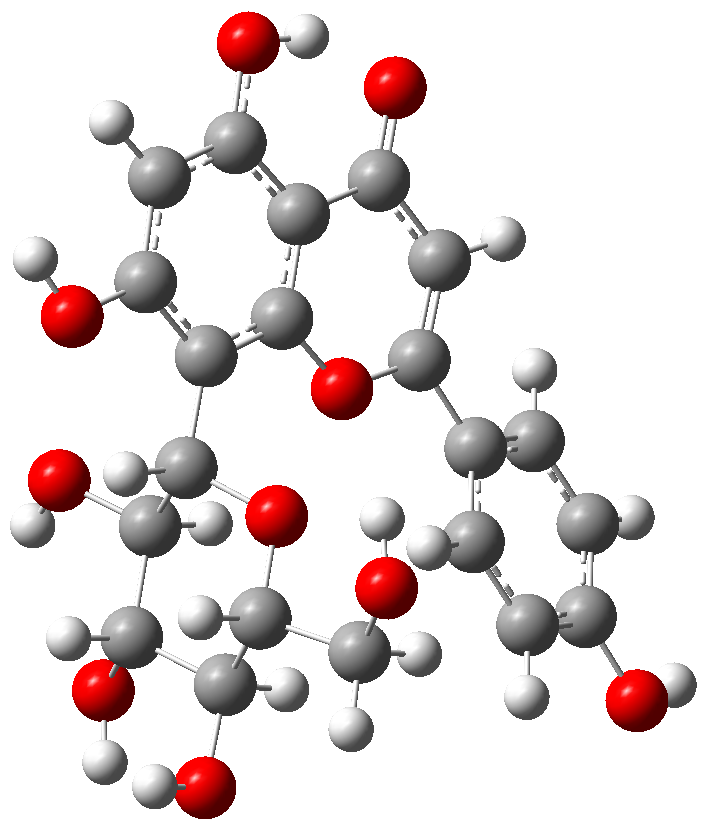 | | |
| --- | --- | --- | --- | --- | --- |
| Center  Number | Atom | Standard orientation: Coordinates (Angstroms) | | | |
|  |  | X | | Y | Z |
| 1 | O | -0.042847 | | -1.730131 | -0.977243 |
| 2 | O | 1.015898 | | -2.003735 | 2.510458 |
| 3 | O | -1.672172 | | -2.911813 | 2.619615 |
| 4 | O | -2.725548 | | -4.015378 | 0.168269 |
| 5 | O | 0.351794 | | 0.958776 | 0.053026 |
| 6 | O | -0.877544 | | -2.56247 | -3.489225 |
| 7 | O | 3.608121 | | -2.459198 | -0.06818 |
| 8 | O | 5.00003 | | 2.087191 | -0.212543 |
| 9 | O | 3.173672 | | 3.895555 | -0.160414 |
| 10 | O | -5.639782 | | 2.774965 | 0.025015 |
| 11 | C | 0.099575 | | -1.839028 | 1.441954 |
| 12 | C | -0.916045 | | -2.980308 | 1.419658 |
| 13 | C | 0.88976 | | -1.849704 | 0.118095 |
| 14 | C | -1.810766 | | -2.916085 | 0.182766 |
| 15 | C | -0.94564 | | -2.835206 | -1.081004 |
| 16 | C | 1.947414 | | -0.780209 | 0.005834 |
| 17 | C | -1.734313 | | -2.615208 | -2.366509 |
| 18 | C | 1.671056 | | 0.591757 | -0.010357 |
| 19 | C | 3.311035 | | -1.130691 | -0.066073 |
| 20 | C | 2.664032 | | 1.582297 | -0.081734 |
| 21 | C | 4.334709 | | -0.179955 | -0.138629 |
| 22 | C | 4.02593 | | 1.17742 | -0.144006 |
| 23 | C | 2.306038 | | 2.992448 | -0.098994 |
| 24 | C | -0.025299 | | 2.267682 | 0.016011 |
| 25 | C | 0.888316 | | 3.272068 | -0.061854 |
| 26 | C | -1.485855 | | 2.418005 | 0.04295 |
| 27 | C | -2.085738 | | 3.61612 | 0.461429 |
| 28 | C | -2.317321 | | 1.357718 | -0.365416 |
| 29 | C | -3.467984 | | 3.758912 | 0.462894 |
| 30 | C | -3.698032 | | 1.493544 | -0.36825 |
| 31 | C | -4.281733 | | 2.697669 | 0.045721 |
| 32 | H | -0.447343 | | -0.890551 | 1.530507 |
| 33 | H | -0.346092 | | -3.928606 | 1.383251 |
| 34 | H | 1.395208 | | -2.820031 | 0.046141 |
| 35 | H | -2.440833 | | -2.022163 | 0.249601 |
| 36 | H | -0.362885 | | -3.769407 | -1.184059 |
| 37 | H | -2.331951 | | -1.693882 | -2.263744 |
| 38 | H | -2.429066 | | -3.446033 | -2.515932 |
| 39 | H | 0.479992 | | -2.145053 | 3.304182 |
| 40 | H | -2.414903 | | -3.524196 | 2.511757 |
| 41 | H | -2.210389 | | -4.825507 | 0.037543 |
| 42 | H | 5.376353 | | -0.482117 | -0.190398 |
| 43 | H | -0.184703 | | -1.925556 | -3.259605 |
| 44 | H | 0.57064 | | 4.304068 | -0.130367 |
| 45 | H | 4.567409 | | -2.568811 | -0.101645 |
| 46 | H | 4.542167 | | 2.979651 | -0.206221 |
| 47 | H | -1.47076 | | 4.438442 | 0.81151 |
| 48 | H | -1.861878 | | 0.430063 | -0.69348 |
| 49 | H | -3.917487 | | 4.690344 | 0.799089 |
| 50 | H | -4.342975 | | 0.683668 | -0.69169 |
| 51 | H | -5.918287 | | 3.650107 | 0.326425 |

**Supplementary Table S20.** Selected Bond Distances (Å) and Angles (deg) for 3-Formyl Chromone Derivatives*^a,b^*

| Bond type | B3LYP/  6-31G (d,p) | B3LYP/  6-311++ (d,p) | B3LYP/  6-311G (d,p) | Exptl^c^ |
| --- | --- | --- | --- | --- |
| **14** | Bond Distances (Å) | | |  |
| C_7_-Br  C_2_-O_1_ C_3_-CHO | 1.905(0.010) 1.338(0.007) 1.480(0.006) | 1.905(0.010) 1.337(0.008) 1.482(0.008) | 1.911(0.016) 1.338(0.007) 1.483(0.009) | 1.895  1.345  1.474 |
| **14** | Bond Angles (deg) | | |  |
| C_7_-C_6_-Br C_2_-O_1_-C_10_ C_4_-C_3_-CHO  **MD** | 119.2(0.0) 118.5(0.5) 124.0(4.0)  0.753 | 119.3(0.1) 118.6(0.6) 124.6(4.6)  0.887 | 119.2(0.0) 118.5(0.5) 124.1(4.1)  0.772 | 119.2 118.0  120.0 |
|  |  |  |  |  |
| **15** | Bond Distances (Å) | | |  |
| C_6_-Cl_1_ C_3_-CHO C_2_-O_1_ | 1.751(0.011)  1.482(0.008) 1.340(0.004) | 1.751(0.011) 1.483(0.007) 1.336(0.008) | 1.751(0.011) 1.484(0.006) 1.338(0.006) | 1.740  1.490  1.344 |
|  | Bond Angles (deg) | | |  |
| C_6_-C_5_-Cl_1_ C_6_-C_7_-Cl_1_ C_4_-C_3_-CHO | 120.0(0.3) 118.6(1.6) 123.9(3.8) | 120.0(0.3) 118.6(1.6) 124.5(3.8) | 120.0(0.3) 118.6(1.6) 124.0(3.3) | 120.3  117.0  120.7 |
| **MD** | 0.953 | 0.954 | 0.870 |  |
| **16** | Bond Distances (Å) | | |  |
| C_6_-Cl_1_ C_3_-CHO C_2_-O_1_ | 1.743(0.028) 1.482(0.004) 1.341(0.003) | 1.743(0.028) 1.483(0.005) 1.337(0.007) | 1.744(0.029) 1.483(0.005) 1.339(0.005) | 1.715  1.478  1.344 |
| **16** | Bond Angles (deg) | | |  |
| C_6_-C_5_-Cl_1_ Cl_1_-C_6_-C_7_ C_4_-C_3_-CHO | 119.2(0.3) 121.1(0.7) 123.9(4.8) | 119.2(0.3) 121.1(0.7) 124.5(4.8) | 119.2(0.3) 121.1(0.7) 123.9(4.2) | 119.5  120.4  119.7 |
| **MD** | 0.972 | 0.973 | 0.873 |  |

**Supplementary Table 21.** Selected IR frequencies data for 3-formyl chromone (**1**), calculated in the gas phase using the B3LYP/6-31G(d,p) method*^a^*

|  |  | **IR, cm^‒1^** | | | | | | | |
| --- | --- | --- | --- | --- | --- | --- | --- | --- | --- |
| **Ligand** |  | ***ν*(C‒H)** | **Calc.** | ***ν*(C‒H (O))** | **Calc.** | ***ν*(C=O)**  **for -CHO** | **Calc.** | ***ν*(C=O)**  **chromone carbonyl** | Calc. |
| **1** | | 3059 | 3009 | 2869 | 2879 | 1700 | 1715 | 1649 | 1656 |
|  |  | ***ν*(C=C)** | **Calc.** | ***ν*(C‒CHO)** | **Calc.** | ***ν*(CH)**  **δ(C‒O‒C)** | **Calc.** | ***ν*(CH)**  **δ(C‒C‒C)** | **Calc.** |
|  |  | 1560 | 1535 | 1360 | 1377 | 1192 | 1191 | 849 | 851 |

^a^Ref. 67

#### **Supplementary Table S22.** Ligand-protein 2D interaction for 6-Isopropyl-3-formylchromone with different proteins.

| **Protein** | Amino acid residue | Bond category | Bond type | Bond Distance | 2D diagram of interaction |
| --- | --- | --- | --- | --- | --- |
| **CAD** | PHE A:114 PHE A:114 | Hydrophobic | Pi-Pi Stacked Pi-Pi Stacked | 6.03  4.51 | 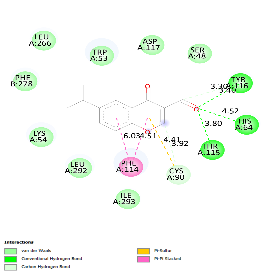 |
|  | CYS A:90 | Miscellaneous | Pi-Sulfur | 4.41 |  |
|  | THR A:115 HIS A: 64 TYR A: 116 TYR A: 116 CYS A:90 | Hydrogen bond | Conventional Hydrogen bond Conventional Hydrogen bond  Conventional Hydrogen bond  Carbon Hydrogen bond Carbon Hydrogen bond | 3.80 4.52 3.40 3.30 3.92 |  |
| **IDE** | TYR A:269 TYR A:269 LEU A:156 ALA A:434 | Hydrophobic | Pi-Pi Stacked Pi-Pi Stacked Pi-Alkyl Alkyl | 4.63 5.00 4.43 5.03 | 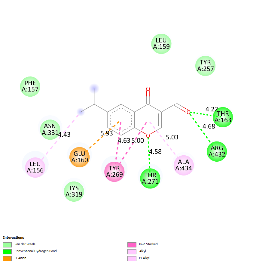 |
|  | THR A:271 ARG A:432 THR A:163 | Hydrogen bond | Conventional Hydrogen bond Conventional Hydrogen bond Conventional Hydrogen bond | 4.58 4.68 4.22 |  |
|  | GLU A:160 | Miscellaneous | Pi-Anion | 5.93 |  |
| **P53** | TRP B:146 TRP B:146 TRP B:146 PRO A:191 | Hydrophobic | Pi-Pi Stacked Pi-Pi Stacked Pi-Sigma Pi-Alkyl | 6.81 5.00 5.42 4.10 | 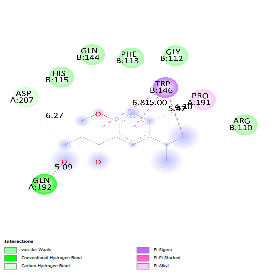 |
|  | GLN A:192 ASP A:207 | Hydrogen bond | Conventional Hydrogen bond Carbon Hydrogen bond | 5.09 6.27 |  |
| **BHK** | TYR A:384 TYR A:384 LEU A:446 ILE A:416 | Hydrophobic | Pi-Pi Stacked Pi-Pi Stacked Pi-Alkyl Pi-Alkyl | 5.48 4.19 4.89 4.98 | 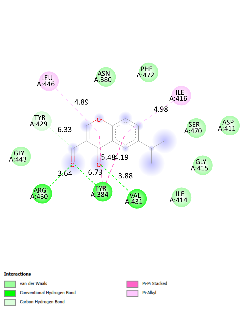 |
|  | ARG A:430 TYR A:384 VAL A:431 TYR A:429 | Hydrogen bond | Conventional Hydrogen bond Conventional Hydrogen bond Conventional Hydrogen bond Carbon Hydrogen bond | 3.64 6.73 3.88 6.33 |  |
| **HIF-α** | HIS A:199 TYR A:102 LEU A:188 ILE A:281 | Hydrophobic | Pi-Sigma Pi-Sigma Pi-Alkyl Pi-Alkyl | 4.90 4.26 5.95 6.03 | 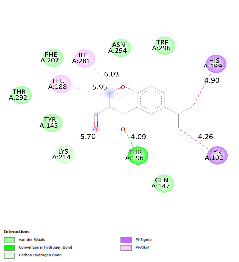 |
|  | THR A:196 LYS A:214 | Hydrogen bond | Conventional Hydrogen bond Carbon Hydrogen bond | 4.09 5.70 |  |
| **Mpro** | THR A:111 GLN A:110 THR A:292 | Hydrogen bond | Conventional Hydrogen bond  Conventional Hydrogen bond  Conventional Hydrogen bond | 4.15 3.31 4.40 | 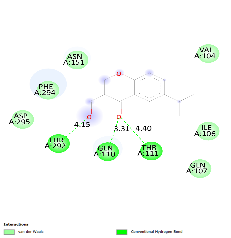 |
| **COX 1** | ALA A:202 | Hydrophobic | Amide-Pi Stacked | 5.04 | 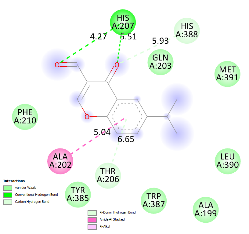 |
|  | HIS A:207 HIS A:207 THR A:206 HIS A:388 | Hydrogen bond | Conventional Hydrogen bond Conventional Hydrogen bond Carbon Hydrogen bond Pi-Donor Hydrogen bond | 4.27 5.51 6.65 5.93 |  |


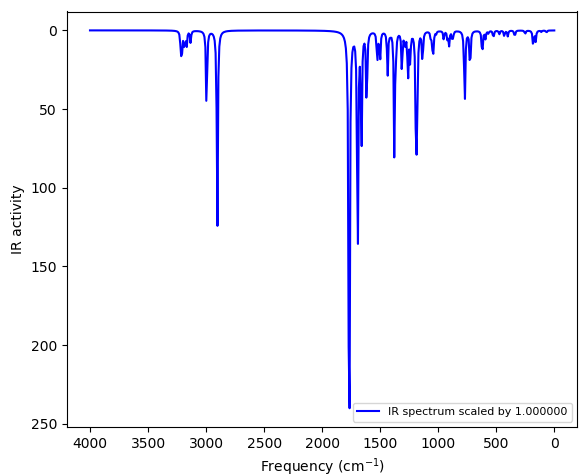


**Supplementary Fig. S1** IR frequencies data for 3-formyl chromone (**1**), calculated in the gas phase using the B3LYP/6-31G(d,p) method.


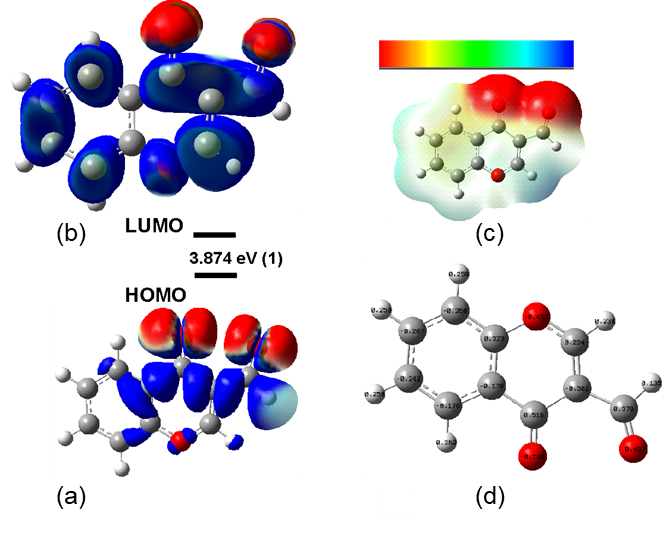


**Supplementary Fig. S2** (a-b) The molecular orbitals of the (a) HOMO and (b) LUMO with Egap; (c) Maps of electrostatic potential (0.02 electrons Bohr−3 surface) (red = electron-rich, blue = electron-deficient) for compound **1**. Regions of respective colours indicating electrophilic (blue) and nucleophilic (red) sites and partial nucleophilic sites (yellow regions); (d) NBO charges structures for compound **1**.


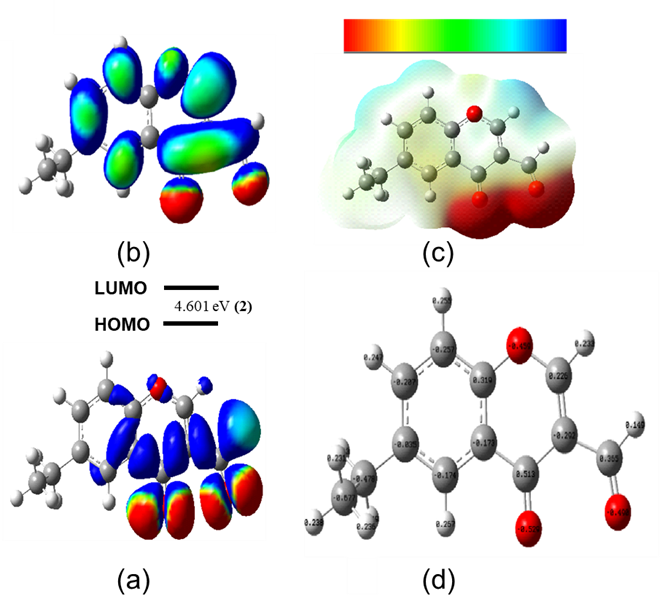


**Supplementary Fig.  S3** (a-b) The molecular orbitals of the (a) HOMO and (b) LUMO with Egap; (c) Maps of electrostatic potential (0.02 electrons Bohr−3 surface) (red = electron-rich, blue = electron-deficient) for compound **2**. Regions of respective colours indicating electrophilic (blue) and nucleophilic (red) sites and partial nucleophilic sites (yellow regions); (d) NBO charges structures for compound **2**.


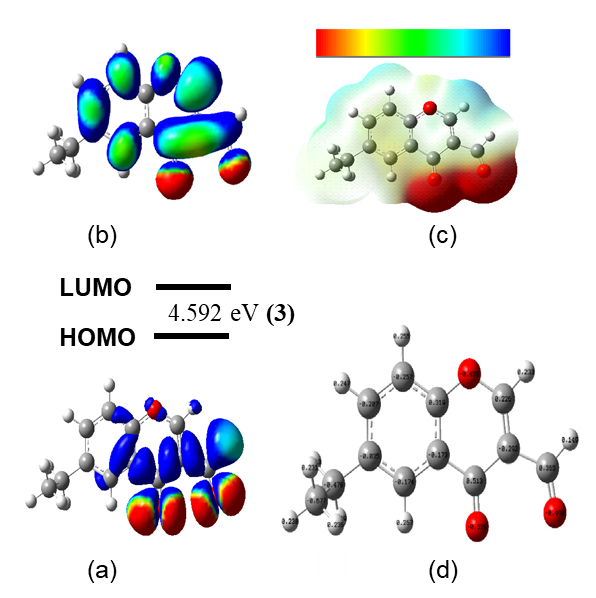


**Supplementary Fig.  S4** (a-b) The molecular orbitals of the (a) HOMO and (b) LUMO with Egap; (c) Maps of electrostatic potential (0.02 electrons Bohr−3 surface) (red = electron-rich, blue = electron-deficient) for compound **3**. Regions of respective colours indicating electrophilic (blue) and nucleophilic (red) sites and partial nucleophilic sites (yellow regions); (d) NBO charges structures for compound **3**.

^
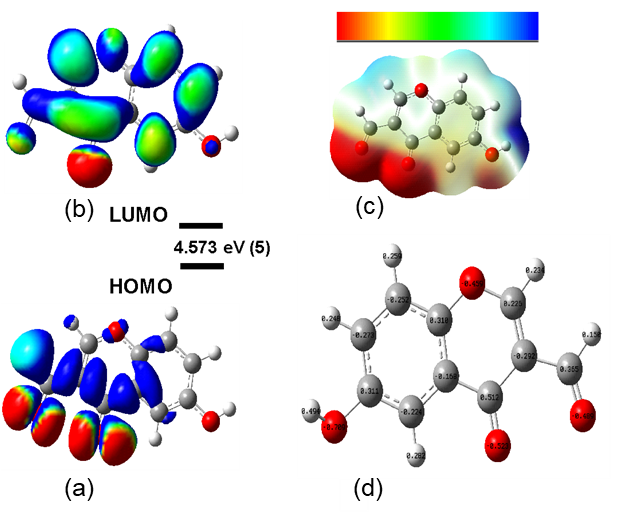
^

**Supplementary Fig.  S5** (a-b) The molecular orbitals of the (a) HOMO and (b) LUMO with Egap; (c) Maps of electrostatic potential (0.02 electrons Bohr−3 surface) (red = electron-rich, blue = electron-deficient) for compound **5**. Regions of respective colours indicating electrophilic (blue) and nucleophilic (red) sites and partial nucleophilic sites (yellow regions); (d) NBO charges structures for compound **5**.

^
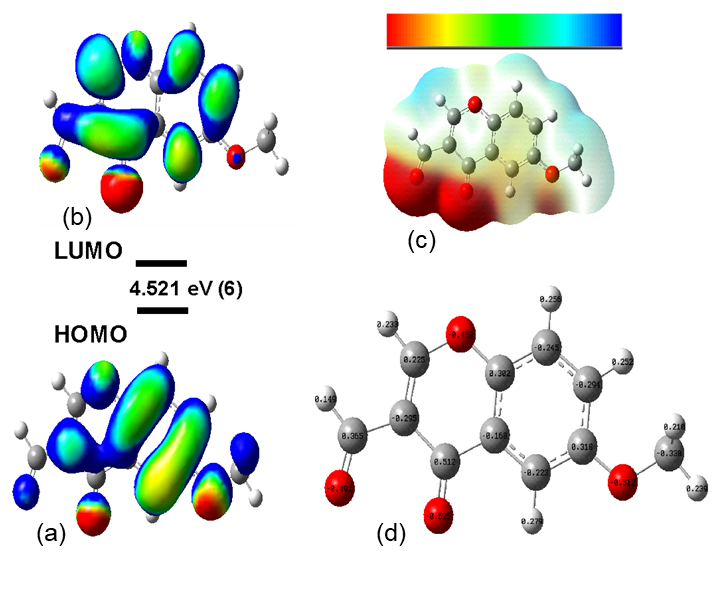
^

**Supplementary Fig.  S6** (a-b) The molecular orbitals of the (a) HOMO and (b) LUMO with Egap; (c) Maps of electrostatic potential (0.02 electrons Bohr−3 surface) (red = electron-rich, blue = electron-deficient) for compound **6**. Regions of respective colours indicating electrophilic (blue) and nucleophilic (red) sites and partial nucleophilic sites (yellow regions); (d) NBO charges structures for compound **6**.


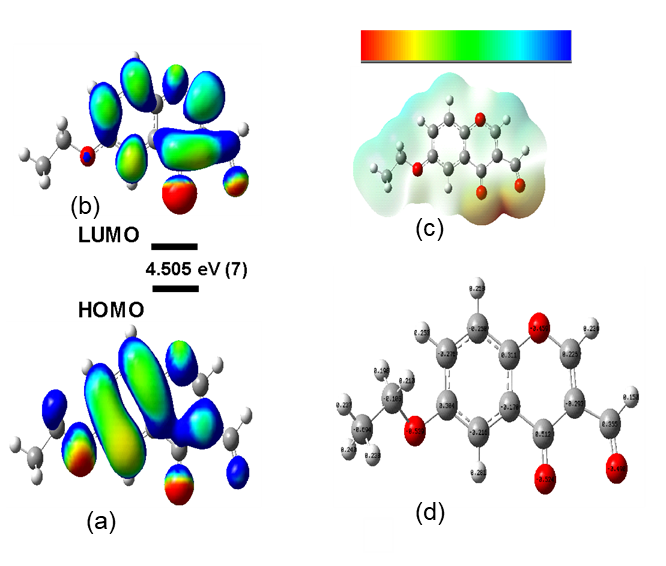


**Supplementary Fig.  S7** (a-b) The molecular orbitals of the (a) HOMO and (b) LUMO with Egap; (c) Maps of electrostatic potential (0.02 electrons Bohr−3 surface) (red = electron-rich, blue = electron-deficient) for compound **7**. Regions of respective colours indicating electrophilic (blue) and nucleophilic (red) sites and partial nucleophilic sites (yellow regions); (d) NBO charges structures for compound **7**.


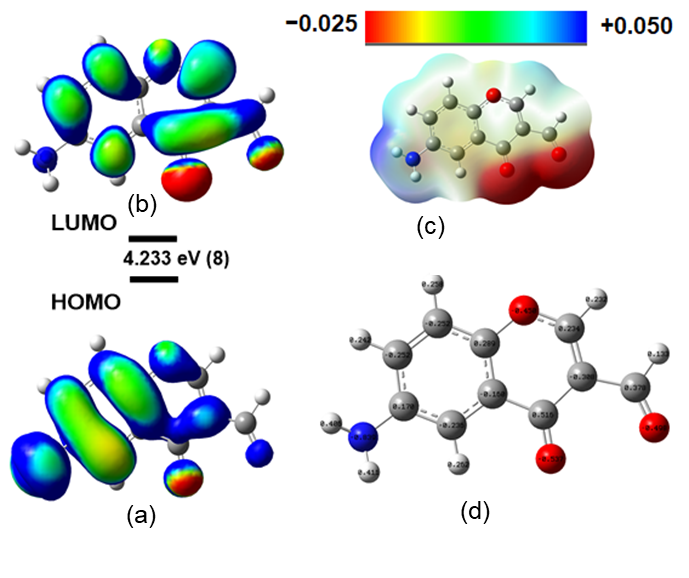


**Supplementary Fig.  S8** (a-b) The molecular orbitals of the (a) HOMO and (b) LUMO with Egap; (c) Maps of electrostatic potential (0.02 electrons Bohr−3 surface) (red = electron-rich, blue = electron-deficient) for compound **8**. Regions of respective colours indicating electrophilic (blue) and nucleophilic (red) sites and partial nucleophilic sites (yellow regions); (d) NBO charges structures for compound **8**.


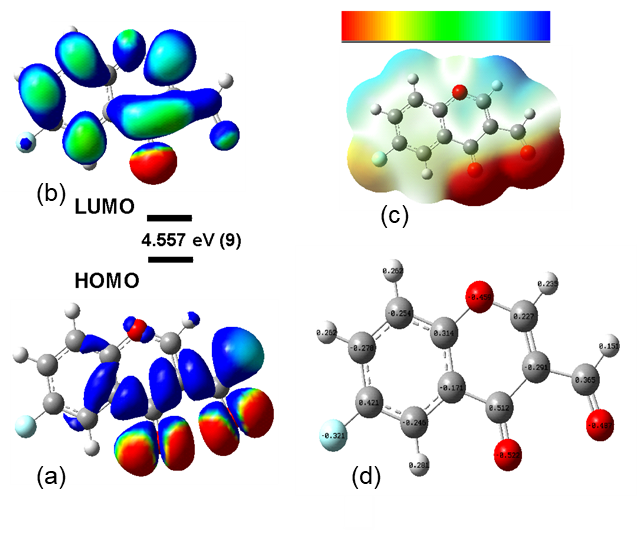


**Supplementary Fig.  S9** (a-b) The molecular orbitals of the (a) HOMO and (b) LUMO with Egap; (c) Maps of electrostatic potential (0.02 electrons Bohr−3 surface) (red = electron-rich, blue = electron-deficient) for compound **9**. Regions of respective colours indicating electrophilic (blue) and nucleophilic (red) sites and partial nucleophilic sites (yellow regions); (d) NBO charges structures for compound **9**.


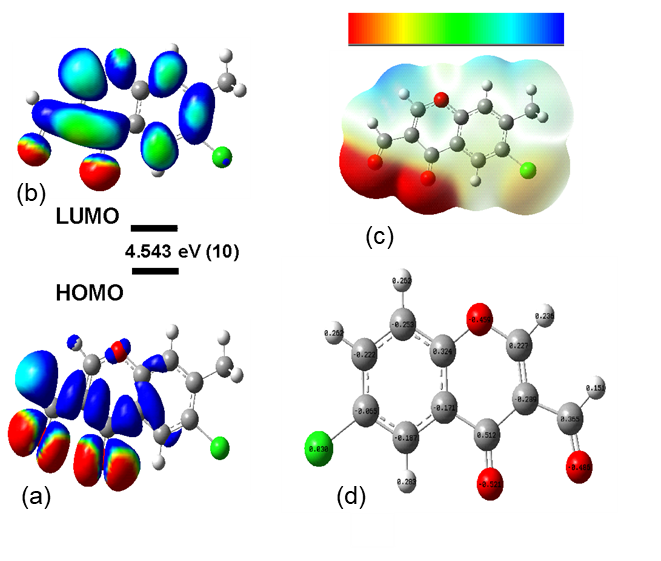


**Supplementary Fig.  S10** (a-b) The molecular orbitals of the HOMO and (b) LUMO with Egap; (c) Maps of electrostatic potential (0.02 electrons Bohr−3 surface) (red = electron-rich, blue = electron-deficient) for compound **10**. Regions of respective colours indicating electrophilic (blue) and nucleophilic (red) sites and partial nucleophilic sites (yellow regions); (d) NBO charges structures for compound **10**.


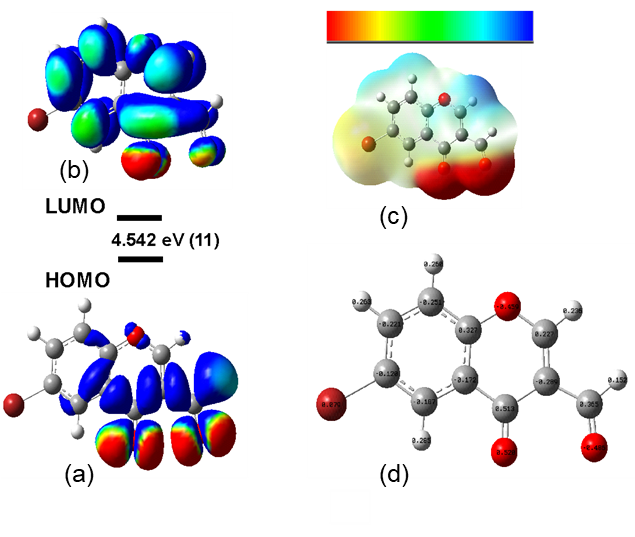


**Supplementary Fig.  S11** (a-b) The molecular orbitals of the (a) HOMO and (b) LUMO with Egap; (c) Maps of electrostatic potential (0.02 electrons Bohr−3 surface) (red = electron-rich, blue = electron-deficient) for compound **11**. Regions of respective colours indicating electrophilic (blue) and nucleophilic (red) sites and partial nucleophilic sites (yellow regions); (d) NBO charges structures for compound **11**.

^
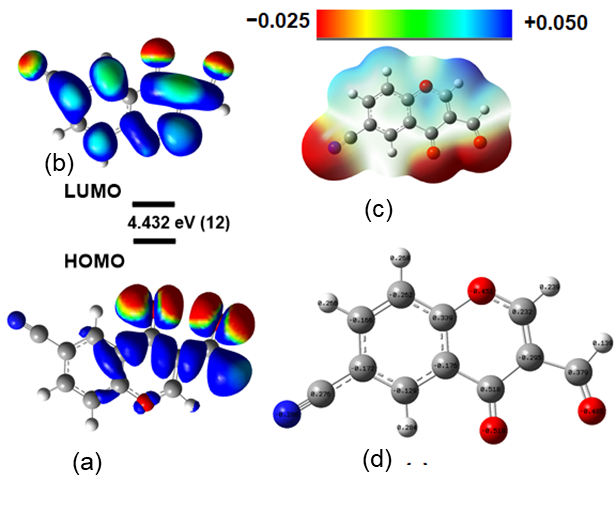
^

**Supplementary Fig.  S12** (a-b) The molecular orbitals of the (a) HOMO and (b) LUMO with Egap; (c) Maps of electrostatic potential (0.02 electrons Bohr−3 surface) (red = electron-rich, blue = electron-deficient) for compound **12**. Regions of respective colours indicating electrophilic (blue) and nucleophilic (red) sites and partial nucleophilic sites (yellow regions); (d) NBO charges structures for compound **12**.

^
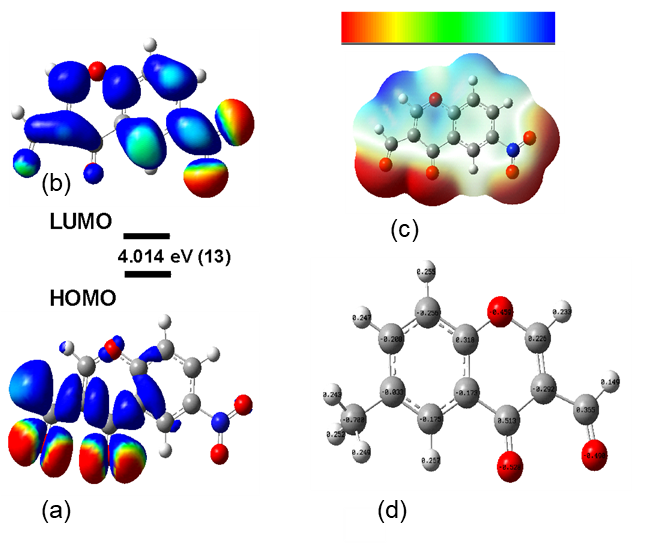
^

**Supplementary Fig.  S13** (a-b) The molecular orbitals of the (a) HOMO and (b) LUMO with Egap; (c) Maps of electrostatic potential (0.02 electrons Bohr−3 surface) (red = electron-rich, blue = electron-deficient) for compound **13**. Regions of respective colours indicating electrophilic (blue) and nucleophilic (red) sites and partial nucleophilic sites (yellow regions); (d) NBO charges structures for compound **13**.

^
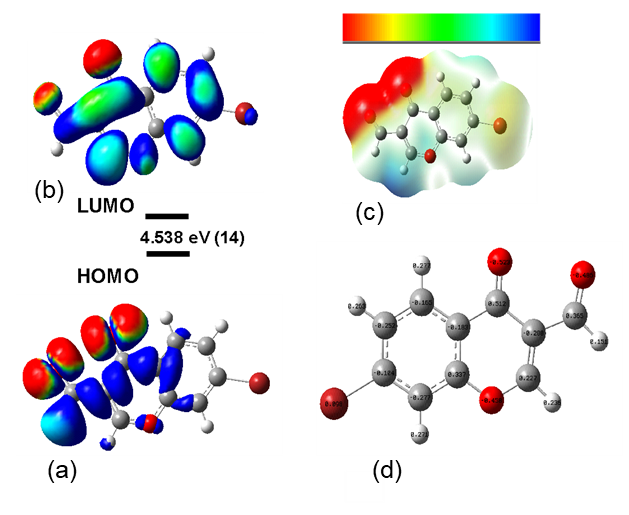
^

**Supplementary Fig.  S14** (a-b) The molecular orbitals of the (a) HOMO and (b) LUMO with Egap; (c) Maps of electrostatic potential (0.02 electrons Bohr−3 surface) (red = electron-rich, blue = electron-deficient) for compound **14**. Regions of respective colours indicating electrophilic (blue) and nucleophilic (red) sites and partial nucleophilic sites (yellow regions); (d) NBO charges structures for compound **14**.

^
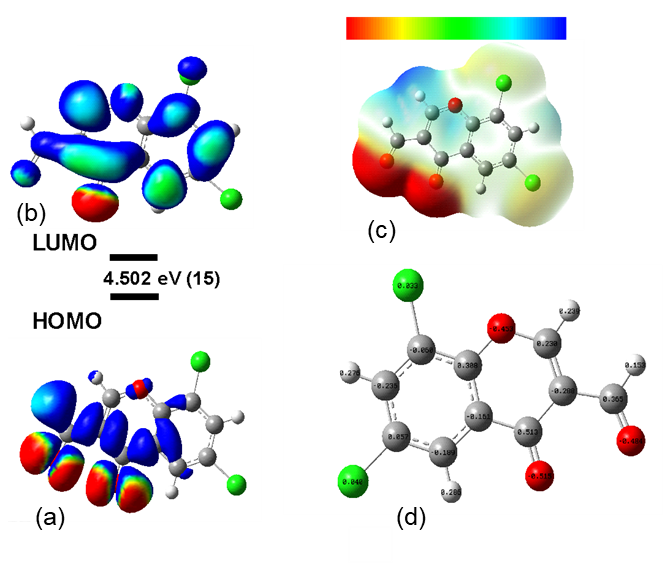
^

**Supplementary Fig.  S15** (a-b) The molecular orbitals of the (a) HOMO and (b) LUMO with Egap; (c) Maps of electrostatic potential (0.02 electrons Bohr−3 surface) (red = electron-rich, blue = electron-deficient) for compound **15**. Regions of respective colours indicating electrophilic (blue) and nucleophilic (red) sites and partial nucleophilic sites (yellow regions); (d) NBO charges structures for compound **15**.

^
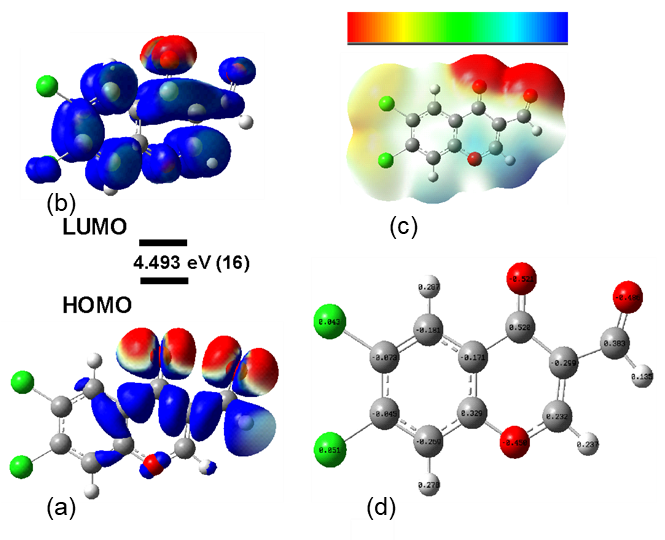
^

**Supplementary Fig.  S16** (a-b) The molecular orbitals of the (a) HOMO and (b) LUMO with Egap; (b) Maps of electrostatic potential (0.02 electrons Bohr−3 surface) (red = electron-rich, blue = electron-deficient) for compound **16**. Regions of respective colours indicating electrophilic (blue) and nucleophilic (red) sites and partial nucleophilic sites (yellow regions); (c) NBO charges structures for compound **16**.


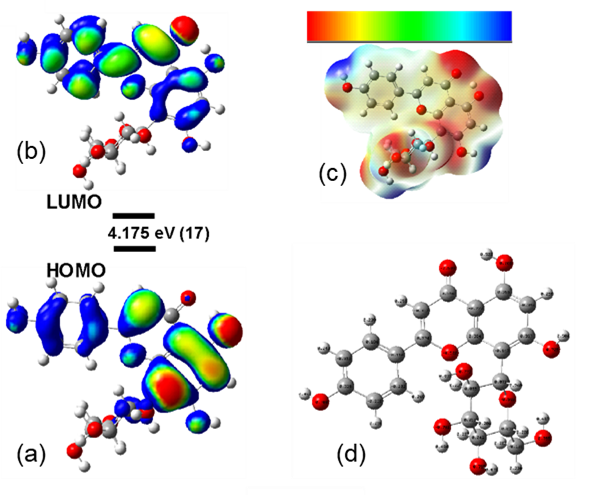


**Supplementary Fig.  S17** (a-b) The molecular orbitals of the (a) HOMO and (b) LUMO with Egap; (c) Maps of electrostatic potential (0.02 electrons Bohr−3 surface) (red = electron-rich, blue = electron-deficient) for compound vitexin **(17)**. Regions of respective colours indicating electrophilic (blue) and nucleophilic (red) sites and partial nucleophilic sites (yellow regions); (d) NBO charges structures for compound vitexin **(17)**.

^
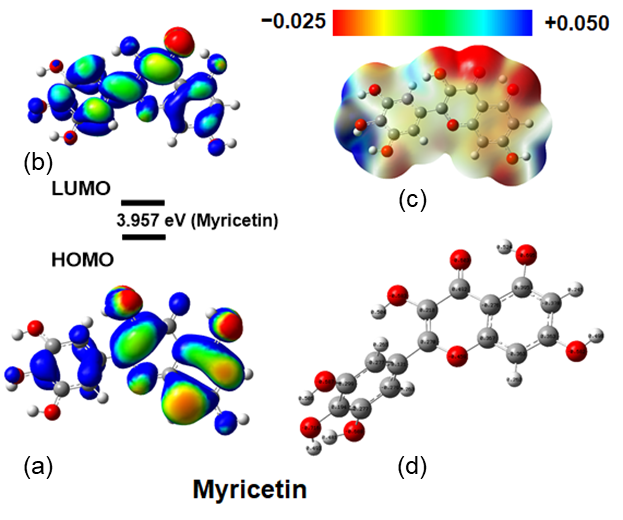
^

**Supplementary Fig.  S18** (a-b) The molecular orbitals of the (a) HOMO and (b) LUMO with Egap; (c) Maps of electrostatic potential (0.02 electrons Bohr−3 surface) (red = electron-rich, blue = electron-deficient) for compound myricetin **(17)**. Regions of respective colours indicating electrophilic (blue) and nucleophilic (red) sites and partial nucleophilic sites (yellow regions); (d) NBO charges structures for compound myricetin **(17)**.

^
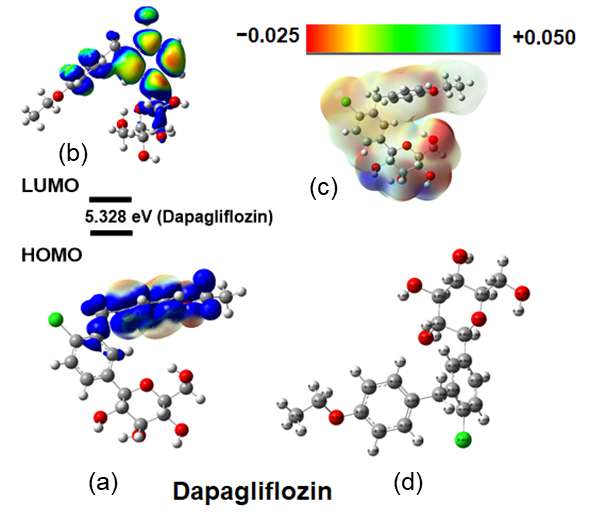
^

**Supplementary Fig.  S19** (a-b) The molecular orbitals of the (a) HOMO and (b) LUMO with Egap; (c) Maps of electrostatic potential (0.02 electrons Bohr^−3^ surface) (red = electron-rich, blue = electron-deficient) for compound dapagliflozin **(18)**. Regions of respective colours indicating electrophilic (blue) and nucleophilic (red) sites and partial nucleophilic sites (yellow regions); (d) NBO charges structures for compound dapagliflozin **(18)**.

^
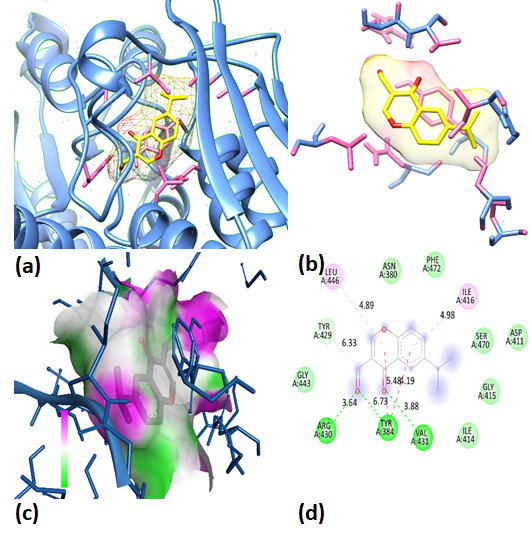
^

**Supplementary Fig.  S20** Molecular docking poses: (a) Ligand in protein pocket; (b) Active site; (c) Hydrogen bonding in solid; (d) Ligand-protein interaction for 2D diagram of compound **4** with PDB (3DGE).


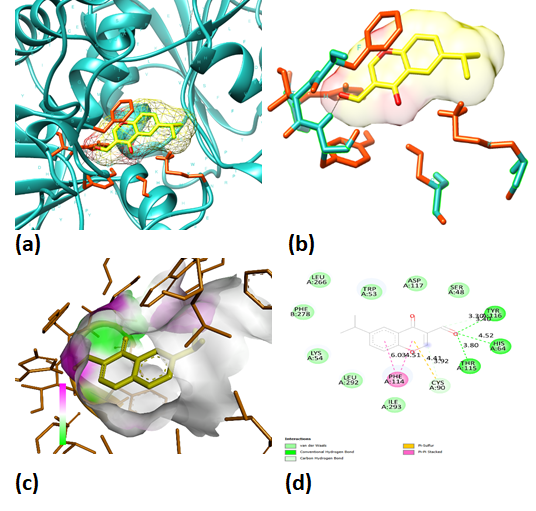


**Supplementary Fig.  S21** Molecular docking poses: (a) Ligand in protein pocket; (b) Active site; (c) Hydrogen bonding in solid; (d) Ligand-protein interaction for 2D diagram of compound **4** with PDB (3TWO).


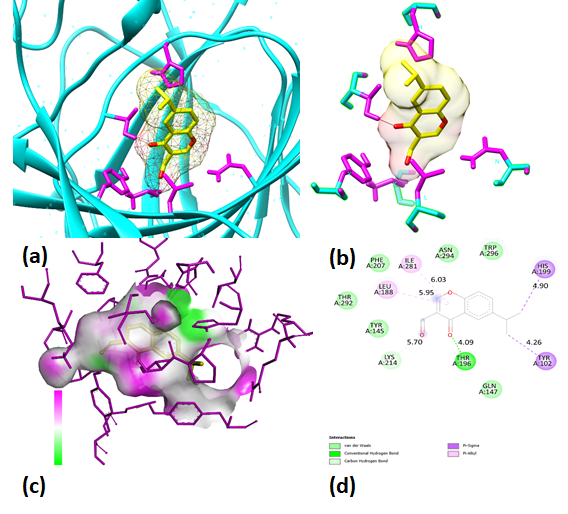


**Supplementary Fig.  S22** Molecular docking poses: (a) Ligand in protein pocket; (b) Active site; (c) Hydrogen bonding in solid; (d) Ligand-protein interaction for 2D diagram of compound **4** with PDB (2WA4).


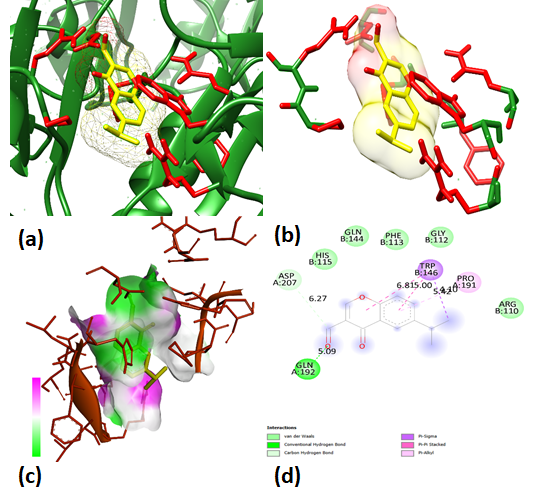


**Supplementary Fig.  S23** Molecular docking poses: (a) Ligand in protein pocket; (b) Active site; (c) Hydrogen bonding in solid; (d) Ligand-protein interaction for 2D diagram of compound **4** with PDB (7EAX).


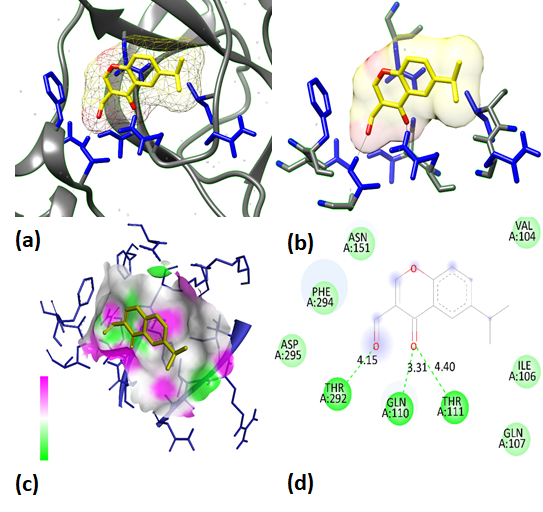


**Supplementary Fig.  S24** Molecular docking poses: (a) Ligand in protein pocket; (b) Active site; (c) Hydrogen bonding in solid; (d) Ligand-protein interaction for 2D diagram of compound **4** with PDB (6LU7).


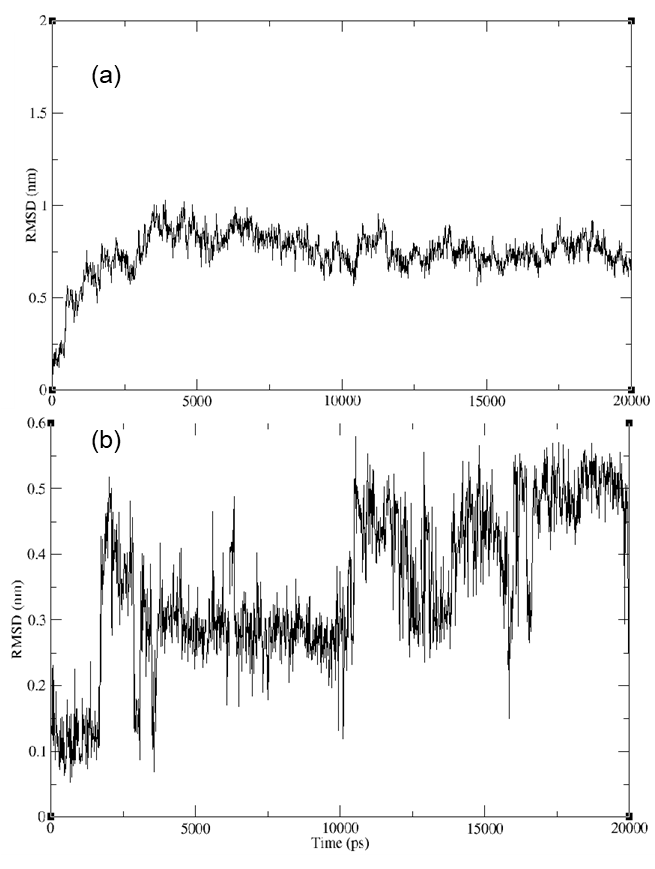


**Supplementary Fig.  S25** RMSD evolution (a) for docked IDE protein-ligand complex; (b) for docked complex COX protein-ligand complex (compound **4**) during 100 ns MD simulation.
